# General, Quantified Structure-Performance Correlations for Synergistic Heteronuclear Electro‑, Polymerization, and Asymmetric Catalysts

**DOI:** 10.1021/acscatal.5c02224

**Published:** 2025-07-16

**Authors:** Katharina H. S. Eisenhardt, Francesca Fiorentini, Frederica Butler, Rosie Thorogood, Charlotte K. Williams

**Affiliations:** Dept. Chemistry, 6396 University of Oxford, 12 Mansfield Road, Oxford OX1 3TA, U.K.

**Keywords:** synergy, homogenous catalysis, structure−activity correlations, electrocatalysis, polymerization catalysis, asymmetric organic transformations

## Abstract

Intermetallic synergy can be useful to both enhance catalytic performance and access diverse chemistries, but understanding and predicting the most effective metal combinations remains a challenge. This perspective focuses on a common class of homogeneous heteronuclear catalysts which combine transition metals (M­(II/III)) with Group III, f-, or s-block metals (M’(I–III)) in phenoxy-imine-ether donor ligands. Independent investigations into these heteronuclear complexes across different fields of catalysis, including electrocatalysis, polymerization catalysis, and small-molecule asymmetric catalysis, have all demonstrated the benefits of intermetallic cooperativity. In both electrocatalysis and polymerization catalysis, similar quantified structure-performance relationships, relating catalytic performance to metal Lewis acidity, have been discovered. This selective perspective article focuses on highlighting the most recent insights and quantified structure–activity relationships in electro- and polymerization catalysis. We then apply the insights and methods, developed separately in these two fields of catalysis, to reanalyze previously reported data for catalytic asymmetric organic transformations. The same quantified structure-performance (i.e., activity or selectivity) trends appear to apply in this third field of homogeneous catalysis. These generally applicable, quantified structure–activity relationships apply to related synergistic heterometallic catalysts across all three disparate fields of chemistry — this finding is both unexpected and very helpful in providing a clearer understanding of how to design catalysts for successful intermetallic synergy. This perspective highlights both the fundamental understanding of such catalytic synergy and the analytical methods used to investigate it. The objective is to provide a guide for both current and future researchers to measure synergistic interactions and to apply those principles to rational catalyst design.

## Graphical abstract

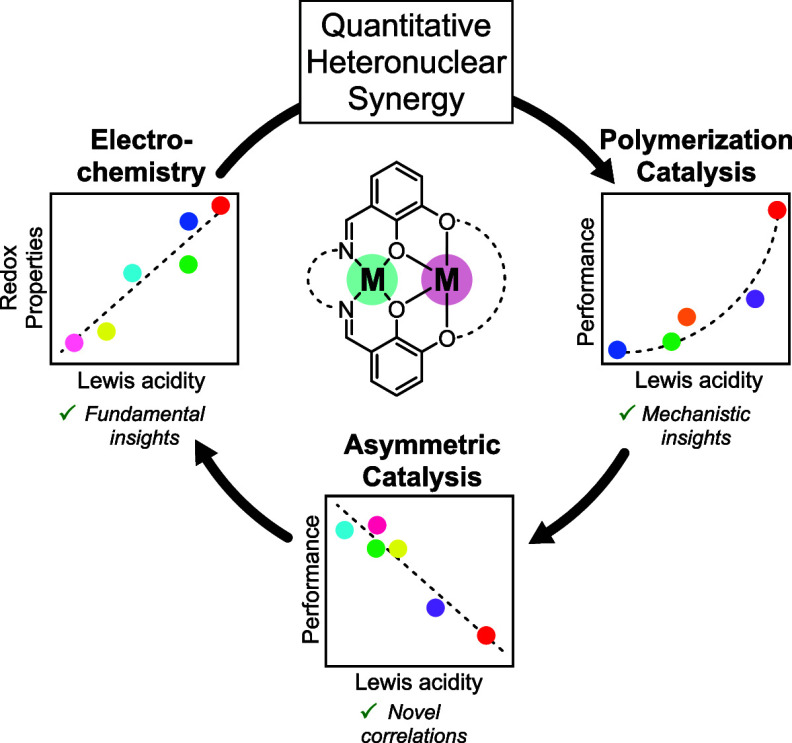

## Introduction

Heteronuclear complexes are attractive in catalysis since cooperative or synergistic interactions between different metals can enhance overall catalyst performance, and/or provide access to new reactivities (Figure 1).^1–10^ Where enhancements in activity are observed for heteronuclear catalysts compared to their monometallic or homonuclear analogs, intermetallic synergy is commonly invoked to rationalize this effect. In this perspective, intermetallic synergy is therefore defined as a type of cooperative behavior in which the performance of a catalyst is greater than that observed for the sum of its parts. Catalysts that exhibit synergy are often referred to as synergistic or synergic. Here, the term synergistic will be used.

**Figure 1:**
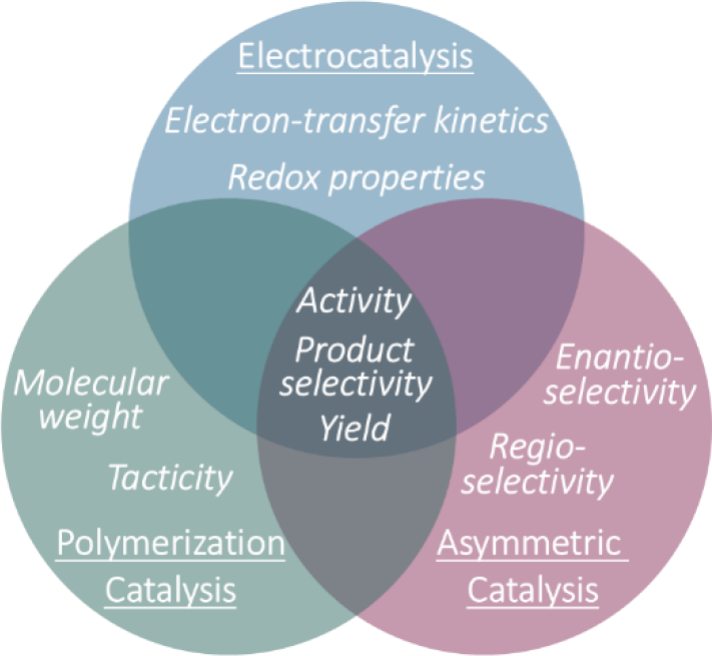
Overview of the effects of intermetallic synergy on catalyst performance across electrocatalysis, polymerization catalysis, and asymmetric catalysis.

Understanding how intermetallic synergy arises and predicting effective metal combinations remain very challenging. This perspective focuses on recent results which provide quantified measures to interpret and explain intermetallic synergy using a family of heterometallic catalysts across three distinct areas of homogeneous catalysis.

In electrocatalysis, intermetallic synergy has been exploited to fine-tune transition metal redox properties and electron-transfer kinetics.^11–14^ In polymerization catalysis, it has been highly effective in increasing both the activity and selectivity for polymerizations of oxygenated monomers, and may be implicated in architecture control in ethylene polymerization.^15^ In asymmetric catalysis, it has been shown to improve activity, enantio-, diastereo-, and regioselectivity.^16–21^ Across all three fields, there are examples in which intermetallic synergy helps to increase rates of catalysis, product selectivity, and yield compared to monometallic and homodinuclear analogues.^16–26^ While catalysts with similar structures have been reported in three fields, investigations into the factors underpinning successful intermetallic synergy within each field have been conducted largely independently. This perspective aims to bridge these three fields, and provide common insights into intermetallic synergy and the methods used to investigate it to aid current and future researchers in these fields, and others, in the rational design of future catalysts.

While the fields of electrocatalysis, polymerization catalysis, and asymmetric organic catalysis, are on the surface, quite disparate, a common ligand family has been employed to make high performance catalysts in all three of these fields (Figure 2).^1,3,9,21,27,28^ This ligand family comprises two coordination sites: one featuring phenoxy-imine donors (i.e., Schiff bases such as salens) and an additional coordination site featuring phenoxy-ether donors. Henceforth, these will be referred to as phenoxy-imine-ether ligands. This ligand family is well-suited to the development of heteronuclear catalysts as it features donors and coordination environments which are matched to the coordination of transition metals (M­(II/III)), or occasionally lanthanides, by the phenoxy-imine donors, and a second, proximal Group III, f-, or s-block metal, by the phenoxy-ether donors (Figure 2).

**Figure 2:**
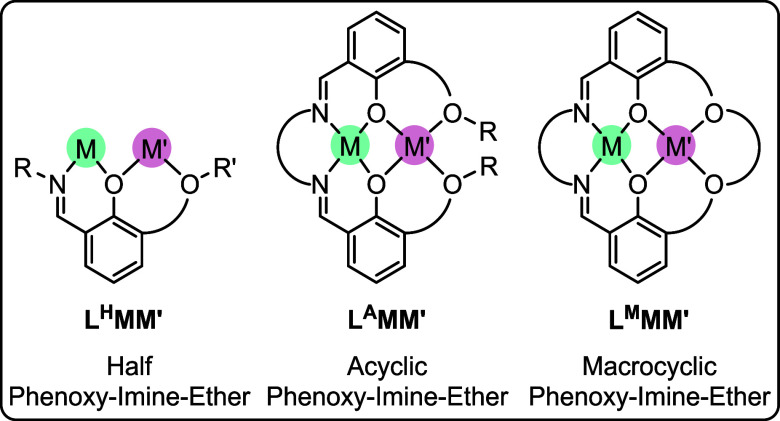
Overview of the ligand structures considered in this perspective, where M is a transition or lanthanide metal coordinated within the imine site, and M’ is a Group I, II, III or lanthanide metal coordinated within the ether site. All ligands are referred to according to the following naming scheme: L^XN^M­(II)­M­(III), where X is H, A, or M and defines the general ligand structure and N refers to the specific ligand structure (Figures S1–S3).

In all three fields of catalysis, many examples of heteronuclear catalysts featuring these phenoxy-imine-ether ligands have been reported, which utilize synergy to achieve outstanding catalytic performances. These complexes are also very well suited to structure-performance investigations since the ligands can coordinate a range of metal centers and oxidation states. There are also sites on the ancillary ligand suitable for structural variation, including the linker between the imine donors, the number of ether donors, the phenolate rings and whether the overall structure is macrocyclic or acyclic. Analysis of the literature reveals three general structures which are particularly effective in heteronuclear homogeneous catalysis: 1) “half” phenoxy-imine-ethers (L^H^); 2) acyclic phenoxy-imine-ether (L^A^); and 3) macrocyclic phenoxy-imine-ethers (L^M^; Figure 2 and Figures S1–S3). It is worth emphasizing that these multimetallic phenoxy-imine-ether catalysts are structurally similar to monometallic salen catalysts which are beyond the scope of this perspective, but have a very rich history of success in catalysis – we direct the reader to various excellent and comprehensive perspectives.^21,28–33^

As an additional benefit, the synthesis of these phenoxy-imine-ether ligands, and their derivatives, is often quite straightforward and high-yielding, with a range of commercial precursors accelerating the systematic investigation of one or both metal coordination environments.^34,35^ This strategy has allowed for quantified structure-performance investigations and resulted in common trends which may help identify the basis of intermetallic synergy.^24,25,36–39^ The most useful quantified structure–activity relationships relate a kinetic parameter – such as reaction rate, yield, or selectivity – to a thermodynamic parameter – such as metal ion pK_a_ or hydrolysis constants, ionic radii, complex spectroscopic signals or crystallographic bond lengths.^40,41^

Despite the interest in quantified structure-performance relationships, many attempts to identify them, including for phenoxy-imine-ether catalysts, fail due to difficulties in identifying the most appropriate or significant thermodynamic parameter. Attempts to correlate rates to measurable parameters in the catalyst ground state, e.g. crystallographic bond lengths, metallic radii, or spectroscopic absorption energies, are often inconclusive. This may be due to some major limitations to the most common catalyst characterization methods such as X-ray crystallography being used to estimate solution-state structures when it applies only to crystalline (solid-state) structures, and the lack of sensitivity of NMR spectroscopy. Optical and vibrational spectroscopies (i.e., IR and UV–vis spectroscopies), and where appropriate, electron paramagnetic resonance (EPR) spectroscopy, are considerably more sensitive techniques and are recommended, but are often more difficult to access and interpret.^12,37^ Further, uncovering quantified structure–activity correlations usually necessitates that the thermodynamic parameter is measured at, or very close to, the active catalytic site. Finally, structure-performance correlations are often complicated because ground-state measurements, even those measurable close to the active site, may not accurately inform upon the catalytic transition state, or intermediate, structures and reactivity.

To circumvent some of the challenges associated with experimentally measured thermodynamic parameters, useful structure–activity relationships, including those discussed in this perspective, have been uncovered using calculated or theoretical scales or measurements, such as metal Lewis acidity.^22,24,42–44^ In this perspective, the s-block metal aqua complex Brønsted acidity, or metal hydrolysis constant (p*K*_a_ or p*K*_H_ respectively), is a very useful thermodynamic parameter used as a measure of metal Lewis acidity. Indeed, as will be explained, it is essential to all the structure-performance relationships across the three fields of catalysis. It is useful since there are a wide range of known metal aqua complex p*K*_a_ values, and these values directly correlate to the much harder to measure and standardize Lewis acidity, at least under some conditions.^45,46^ One challenge is that metal aqua complex p*K*_a_ values may fail to account for specific solvent effects or structural changes associated with the Lewis acidic metal coordination environment.^47^ The measurement of metal complex Lewis acidity, pertinent to many catalysts and reactions, is much more challenging, but there are a range of titrations employed to estimate and compare values, such as the Gutmann-Beckett method. In this method the target s-block metal complex or ion is titrated with triphenyl phosphine oxide, and changes to the ^31^P­{^1^H} NMR chemical shifts are used to quantify its relative Lewis acidity.^45^ The use of different probes and titrations across, and even within, different studies, means there are no uniform or commonly used scales to quantify metal complex Lewis acidity (vs. metal aqua complex p*K*_a_ as a proxy measure for Lewis acidity).

When applying quantified structure–activity relationships to study intermetallic synergy, it is also very important to consider the origin and quality of the kinetic data used. Such data can be obtained either by point kinetic measurements, such as yield or turnover frequency (TOF) in which the product conversion is determined at a specific time-point. A preferable method to compare catalyst activities is to use a rate constant, which is derived from conversion data measured at multiple time-points. While point kinetic measurements may be easier and faster to obtain, rate constants are much more accurate and are highly recommended for these quantified structure-performance studies. This is because rate constants are independent of conversion (unlike point measurements for non-zero order reactions), are more precise due to the use of multiple time point measurements, and allow for proper consideration of factors such as initiation, termination or catalyst decomposition, all of which can affect the measured rate. In this perspective, we have, where feasible, chosen to plot rate constants as the kinetic parameter instead of point kinetic values.

While previous studies have investigated the effect of Lewis acidity, described by p*K*_a_, on the properties and catalytic performance of other s-and f-block containing structures, such as monometallic metal salts and cubane complexes, the first quantified correlations for this family of phenoxy-imine-ether heteronuclear complexes were reported for L^M^M­(II/III)­M­(I–III) complexes.^44,48^ Since 2017, it has been repeatedly found that transition metal (M­(II/III)) redox potentials and electron transfer parameters correlated directly with the s-block, lanthanide or Group III metal (M­(I–III)) Lewis acidity as measured by aqua complex p*K*_a_.^11^ In 2020, we reported related L^M^Co­(III)­M­(I/II) catalysts which showed very high activity and selectivity for carbon dioxide/epoxide ring-opening copolymerizations (ROCOP), epoxide/anhydride ROCOP and lactide (LA) ring-opening polymerizations (ROP).^24^ We showed that across all three polymerizations, there were quantifiable correlations between both catalytic activity and selectivity and the metal Lewis acidity (as measured by aqua complex p*K*_a_). These new correlations were combined with comprehensive investigations of the polymerization kinetics (rate laws and Eyring analyses), computational catalytic cycles and experimental catalyst characterization to support a dinuclear catalytic mechanism.

In the field of asymmetric organic transformations, Kobayashi and co-workers reported on the dependence of catalytic activity on cation Lewis acidity, as measured by aqua complex p*K*_a_, for the Mukaiyama aldol reaction catalyzed by simple metal salts in water in 1998.^48^ However, prior to this perspective, there did not seem to be any quantified structure–activity relationships explicitly reported for heterometallic catalysts of the family featured in this perspective used for asymmetric organic transformations, despite their long-standing precedent in that field.^16–21^ In conducting our literature perspective, we decided to reanalyze the originally-reported data from some of the leading asymmetric catalysis publications. Our analysis reveals a common series of quantified structure-performance relationships which are directly analogous to those already established in the electro- and oxygenated monomer polymerization catalysis. It is important to emphasize that these quantified structure-performance correlations always serve to substantiate the descriptive mechanistic hypotheses provided by the original authors – i.e. they may be useful in providing further support for mechanisms. One challenge which is especially relevant in analyzing data from the asymmetric catalysis field is that the field has generally taken a pragmatic approach to catalyst development, often combining ligands and metal salts *in situ* with reagents and solvents (rather than isolating complexes), using high loadings of ligand and metal salts, and reporting only point kinetic measurements (e.g., turnover frequency or yield rather than rate coefficients and laws). This means that some caution should be taken in interpreting and quantifying the results, and it is important to note that correlations do not necessarily imply causation and can only be interpreted in the context of other, mechanistic studies. Despite its potential limitations, this high-throughput screening approach has furnished a lot of experimental data which is certainly a benefit in establishing the generality of these structure-performance correlations.

## Electrocatalysis

Electrocatalysis is fundamentally dependent on both the thermodynamics, most commonly measured through the redox potential at which a reaction occurs, and the electron transfer kinetics (i.e., electron transfer rate) of the relevant reaction.^14,22,23^ Over the past four decades, many studies have suggested that intermetallic synergy can affect both the thermodynamics and kinetics of electrocatalysis.

Over the past decade, the effects of a non redox active s-block, Group III or lanthanide metal cation bound at a defined distance within a macrocyclic phenoxy-imine-ether ligand framework (L^M^), on the properties of different transition metals, including Co­(II), Fe­(II), V­(IV), Ni­(II), Pd­(II) and Zn­(II), have been systematically investigated.^1,11–14,22,23,36,37,42,47,49–55^ Other researchers have applied different ligands, such as nitrogen-containing pincer ligands, or used s-block metals to influence actinide or lanthanide active sites.^2,49,56^ Notably, in heterometallic cubane complexes, linear correlations between the p*K*_a_ of metal cations and the redox properties of manganese centers have been reported, but such studies are beyond the scope of this perspective.^44^

A 1990 report demonstrated that Ba­(II) coordinated close to a Ni­(II) center, by an L^M1^ ligand, leads to a shift toward less negative redox potentials for the Ni­(II/I) redox couple (shifted by 0.33 V, in DMF and DMSO).^57^ More recently, in 2017, Yang and co-workers reported the first systematic study of a series of L^M2^Co­(II)­M­(I/II) complexes to investigate how the s-block metals influenced the transition metal (Figure 3).^11^ Coordination of Na­(I), K­(I), Ca­(II), Sr­(II) or Ba­(II), by the phenoxy-ethers of L^M2^, caused a positive shift in the redox potential of the Co­(II) center in Co­(II)­M­(I/II) complexes compared to the monometallic Co­(II) complex (E_1/2, Monometallic_ = −1.71 V). A linear correlation between *E*_1/2_(Co­(II/I)) and Lewis acidity of the s-block metal (measured by the p*K*_a_ of the metal aqua complex) was observed (Figure 3; top LHS). Subsequently, Blakemore and co-workers investigated the effects of non redox active metals on a Ni­(II) center using a similar L^M3^ ligand; they observed that coordination of Nd­(III), Y­(III), Ca­(II), or Na­(I), resulted in significant shifting of the Ni­(II/I) reduction potential to less negative potentials compared to the monometallic Ni­(II) complex (*E*_p,c__Monometallic_ = −1.89 V).^12^ In-line with the report by Yang and co-workers, it was observed that this shift in redox potential linearly correlated with the p*K*_a_ of the corresponding M­(I–III) aqua complex (Figure 3; bottom LHS).

**Figure 3:**
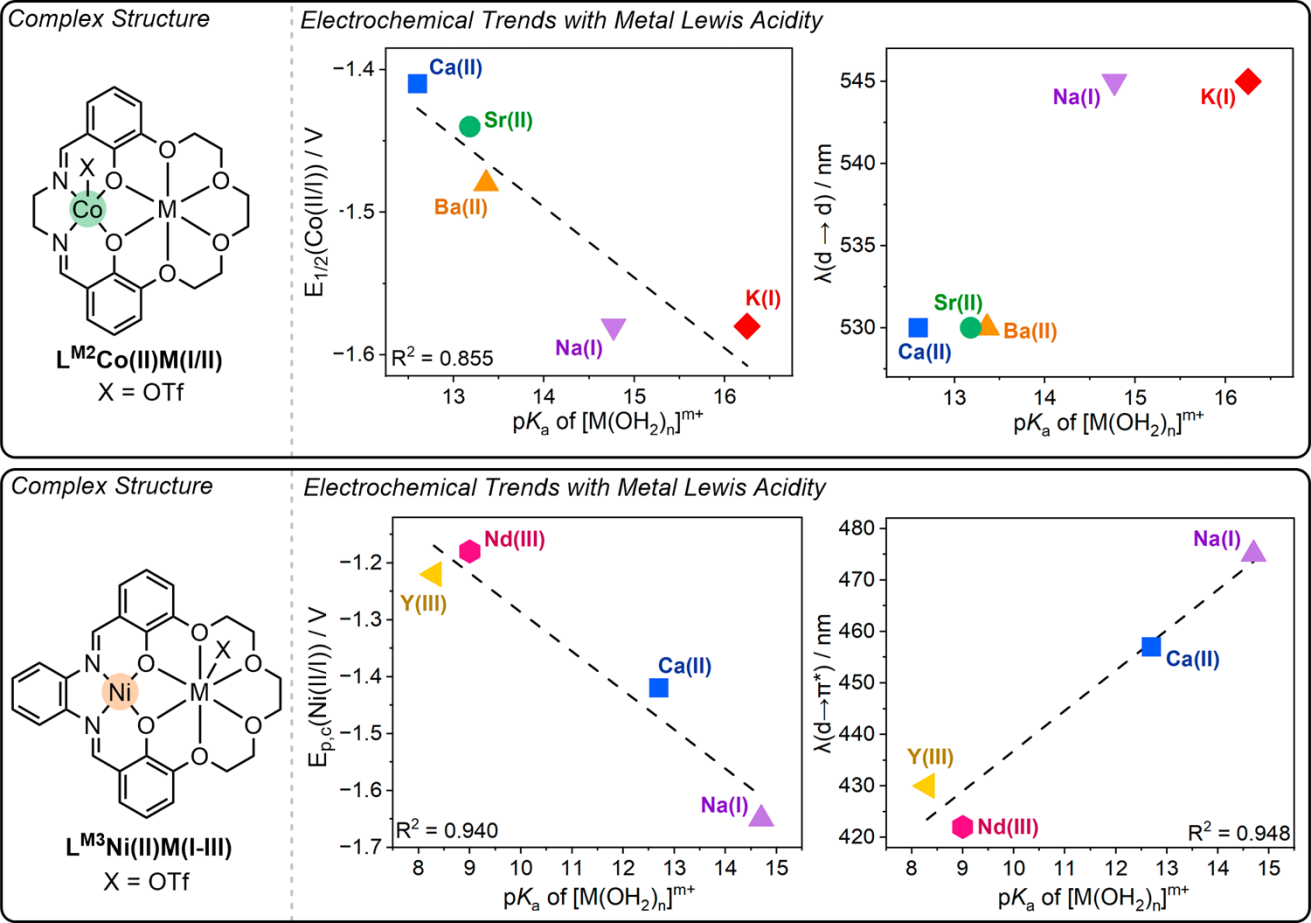
Effects of changing proximal cation Lewis acidity (as measured by aqua complex p*K*_a_) on transition metal redox and spectroscopic properties, as reported by Yang and co-workers (top), and Blakemore and co-workers (bottom), on L^M2^Co­(II)­M­(I/II) and L^M3^Ni­(II)­M­(I–III) complexes, respectively.^11,12^ *E*_1/2_ or *E*_p,c_ (vs Fc^I/0^) of both transition metals linearly depend on proximal cation Lewis acidity. For L^M2^Co­(II)­M­(I/II), no significant change in the d → d transition was observed with changing M­(I/II). A linear correlation between the position of the metal-to-ligand charge transfer band and M­(I–III) Lewis acidity for L^M3^Ni­(II)­M­(I–III) was observed.^11,12^ Plots were adapted from ref. 11. Copyright 2017 American Chemical Society (top) and with permission from ref. 12. Copyright 2018 John Wiley and Sons.

Following these early reports of linear correlations between electrochemical properties of transition metals and the Lewis acidity of the proximal, non redox active cations, several other reports have observed similar linear trends for different Ni­(II) complexes, as well as Cu­(II), Pd­(II) and V­(IV) complexes.^12,37,52–55^ Recently, Blakemore and co-workers demonstrated another linear correlation between the *E*_1/2_ of Ni­(II)­M­(I–III) complexes (where M­(I–III) = K­(I), Na­(I), Li­(I), Sr­(II), Ca­(II), Zn­(II), La­(III) and Lu­(III)) with the aqua complex p*K*_a_ of the M­(I–III) cation, using an acyclic L^A1^ ligand (Figure S4).^51^ This is a significant finding since the synthesis of L^A^H_2_ is significantly easier than that of analogous macrocyclic L^M^H_2_, as macrocycle synthesis requires the use of a templating agent to prevent formation of polymeric products.^51^

While linear correlations between the *E*_1/2_ of the transition metal center and the Lewis acidity of the second metal (measured by its metal aqua complex p*K*_a_) have been consistently reported over a wide range of p*K*_a_ units, the origin of this effect remains less clear. It is generally proposed that the physical origin of intermetallic synergy in L^M^M­(II)­M­(I–III) complexes could either be an electrostatic interaction between the two metal centers or an inductive effect facilitated by the phenoxy bridge in the ligand framework (Figure 4). Both effects are expected to lead to a stabilization of the transition metal-centered highest occupied molecular orbital (HOMO) upon M­(I–III) coordination, rationalizing the linear relationship between the transition metal *E*_1/2_ and the Lewis acidity of M­(I–III). Upon M­(I–III) cation coordination, an electrostatic effect would be expected to result in a shift to both the HOMO and LUMO in parallel, preserving a constant HOMO–LUMO gap (Figure 4, LHS).^36,54,55^ In contrast, an inductive effect has been proposed to only affect the transition metal ligand field (i.e., lowering the energy of the HOMO), leading to a change in HOMO–LUMO gap upon coordination of different M­(I–III) cations (Figure 4, RHS).^12,52,55^ Any significant variations in the frontier molecular orbital separation (HOMO–LUMO gap) should be detectable by optical spectroscopy, due to its high sensitivity.

**Figure 4:**
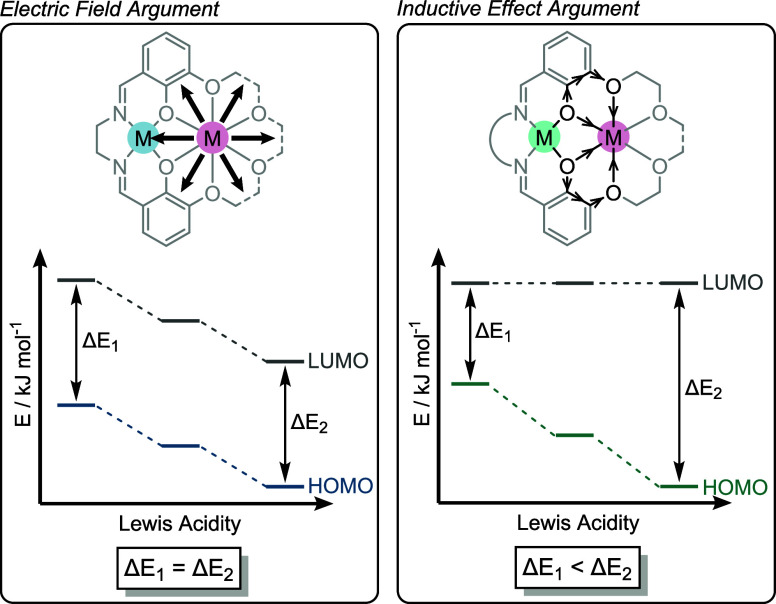
LHS: Yang and co-workers have proposed that intermetallic synergy is primarily an electrostatic effect, leading to a stabilization of the frontier molecular orbitals.^11,36^ Diagram adapted from ref. 36. Available under a CC-BY 3.0 licence. Copright 2019 Kang et al. RHS: Blakemore and co-workers have proposed that intermetallic synergy mainly originates from an inductive effect, facilitated by the phenoxide bridge, leading to an increase in the HOMO–LUMO gap.^12,37^

Yang and co-workers have largely attributed linear correlations between transition metal *E*_1/2_ and proximal metal Lewis acidity to electrostatic interactions, with inductive effects through the phenoxide bridge proposed to play a smaller role.^11^ In the initial investigation of L^M2^Co­(II)­M­(I/II) complexes_,_ only small changes in the UV–vis absorption spectrum (Δλ = 15 nm) of the L^M1^Co­(II)­M­(I/II) complexes were observed using different M­(I/II), and no correlation between absorption maxima with the s-block metal Lewis acidity was observed (Figure 3, top RHS). It was therefore proposed that the effect of M­(I/II) on the Co­(II) ligand field is minimal and, hence, the origin of the quantified structure–activity relationships was mainly electrostatic. In order to further differentiate between electrostatic effects and inductive effects, Yang and co-workers utilized the L^M2^ ligand to investigate the effect of s-block M­(I/II) coordination on a Ni­(II) center (Figure S5).^36^

Upon coordination of either Na­(I) or Ba­(II) in the L^M2^Ni­(II) complex, positive shifts in the *E*_1/2_ of the Ni­(II/I) redox event by 120 mV for Na­(I) and 340 mV for Ba­(II) were observed. For both complexes, the UV–vis spectra showed no significant change in the π → π*, d → d, or the metal-to-ligand charge transfer bands compared to the mono-Ni­(II) complex. Based on the negligible changes in the absorption spectra, and therefore the Ni­(II) ligand fields, upon s-block metal coordination, the major effect of the proximal cations on the adjacent transition metal was proposed to be an electrostatic interaction. The group further probed this hypothesis using density functional theory (DFT) calculations (ωB97X-D9, def2-SVP and def2-TZVP basis set), investigating the effect of M­(I/II) coordination of the Ni­(II) frontier molecular orbitals.^36^ The DFT calculations indicated that all frontier molecular orbitals are shifted in a parallel fashion upon coordination of the M­(I/II) ions, leading to an unchanged HOMO–LUMO gap, in agreement with the absorption spectra (Figure 4, LHS). It was proposed that the electric field generated by the cation stabilizes the transition metal orbitals without affecting their relative energies. Subsequent DFT studies, by Ghosh and co-workers, further implicated an electric field between non redox active metals (Li­(I), Na­(I), K­(I), Mg­(II), Ca­(II), Zn­(II), Cd­(II), Pr­(III), Nd­(III), Sm­(III)) and transition metal cations (Cu­(II), V­(IV), Ni­(II)), agreeing with the hypothesis initially proposed by Yang and co-workers.^53–55^ Similar to the first theoretical study by Yang and co-workers, Ghosh and co-workers also proposed that the electric field stabilizes the transition metal orbitals, rationalizing the shifts in electrochemical behavior.^53–55^ In contrast to Yang and co-workers, Blakemore and co-workers attribute the observed linear correlation of transition metal *E*_1/2_ with non redox active cation Lewis acidity principally to an inductive effect, facilitated by the phenoxide bridge in the ligand (Figure 4, RHS).^12,37^

The inductive effect is proposed to stabilize the transition metal HOMO, thus increasing the HOMO–LUMO gap. Using L^M3^Ni­(II)­M­(I–III) complexes, a linear correlation between the lowest energy of metal-to-ligand charge transfer band observed by UV–vis absorption spectroscopy (Δλ = 55 nm) and the Lewis acidity of the cation was observed (Figure 3, bottom RHS). It was proposed that the observed UV–vis absorption shift is indicative of a stabilization of the Ni­(II)-centered HOMO, caused by the proposed inductive effect, resulting from reduced donation by the phenoxy donor, after coordination of the M­(I–III) center. This stabilization effect is proposed to lead to an increased HOMO–LUMO gap (46 meV/p*K*_a_ unit), observable by UV–vis spectroscopy. Blakemore and co-workers highlighted that the M­(I–III) coordination is expected to have a larger effect on the metal-centered HOMO than the ligand-centered LUMO, consistent with the observed correlation between *E*_1/2_ and Lewis acidity. In line with the results for the L^M3^Ni­(II)­M­(I–III) complexes, a second linear correlation between the energy of the d→d transition and the Lewis acidity of the cation was also observed for the L^M1^Ni­(II)­M­(I–III) complexes (where M­(I–III) = Na­(I), K­(I), Sr­(II), Ca­(II), La­(III), Lu­(III), Y­(III), Figure S6).^51^ This correlation is proposed to be indicative of increased stabilization of the HOMO, similar to that observed for the analogous complexes using the L^M2^ framework. In this study of L^M1^Ni­(II)­M­(I–III) complexes, Blakemore and co-workers noted a slope close to unity for the linear correlation between *E*_1/2_(Ni­(II/I)) and the M­(I–III) aqua complex *K*_a_ (obtained from p*K*_a_; 2.303·RT·log­(*K*_a_) = 59.1 mV; Figure S6). It was proposed that this could indicate that the M­(I–III) center exhibits an electrostatic influence on the Ni­(II) center.^14^ However, the authors proposed this effect to be secondary to the dominant inductive effect through the phenoxide bridge. This study highlights the difficulties in separating the influences due to electrostatic and inductive effects, as these could also be acting in conjunction with each other.

In order to further probe the origin of intermetallic synergy, Blakemore and co-workers investigated a series of L^M1^[VO]­(II)­M­(I/II) complexes (where M­(I/II) = Cs­(I), Rb­(I), K­(I), Na­(I) and Ca­(II); Figure S7).^37^ Vanadyl ([VO]­(II)) was selected as the transition metal center as it allowed the use of electron paramagnetic resonance spectroscopy (EPR) to study the electron donating ability of the equatorial ligands surrounding the vanadyl center (V­(IV), 3d^1^). An exponential shift of the hyperfine coupling constant with Lewis acidity of M­(I/II) was observed. It was proposed that the hyperfine coupling constant correlates with the strength of interaction between the vanadyl center and the two axial oxo and four equatorial ligands. This correlation was proposed to be indicative of decreased donor strength of the phenoxide ligand to the vanadyl center after M­(I/II) coordination. Based on this result, the authors proposed that the synergistic interaction between the metals in L^M^ ligands is mainly facilitated by the bridging phenoxide donor which, upon M­(I/II) coordination, shows a decreased donor strength to the transition metal. Most electrochemical studies investigating intermetallic synergy in L^A^MM­(I–III) and L^M^MM­(I–III) complexes have focused on the effect on the thermodynamic parameters, i.e. redox potential, of the coordinated transition metal. Only recently, Blakemore and co-workers reported upon the effect of proximal M­(I–III) cations on the transition metal electron transfer kinetics.^13,14^ In 2022, they investigated the effect of M­(I–III) (where M­(I–III) = K­(I), Na­(I), Sr­(II), Ca­(II), La­(III), Y­(III), Lu­(III)) on the kinetics of Pd­(II/I) reduction in L^M1^Pd­(II)­M­(I–III) complexes (Figure 5, top).^13^ In addition to the linear trend of the Pd­(II/I) reduction potential with M­(I–III) Lewis acidity, it was also observed that coordination of more Lewis acidic metals such as Lu­(III) resulted in a broader Pd­(II/I) reduction wave compared to complexes with less Lewis acidic metals such as Na­(I).

**Figure 5:**
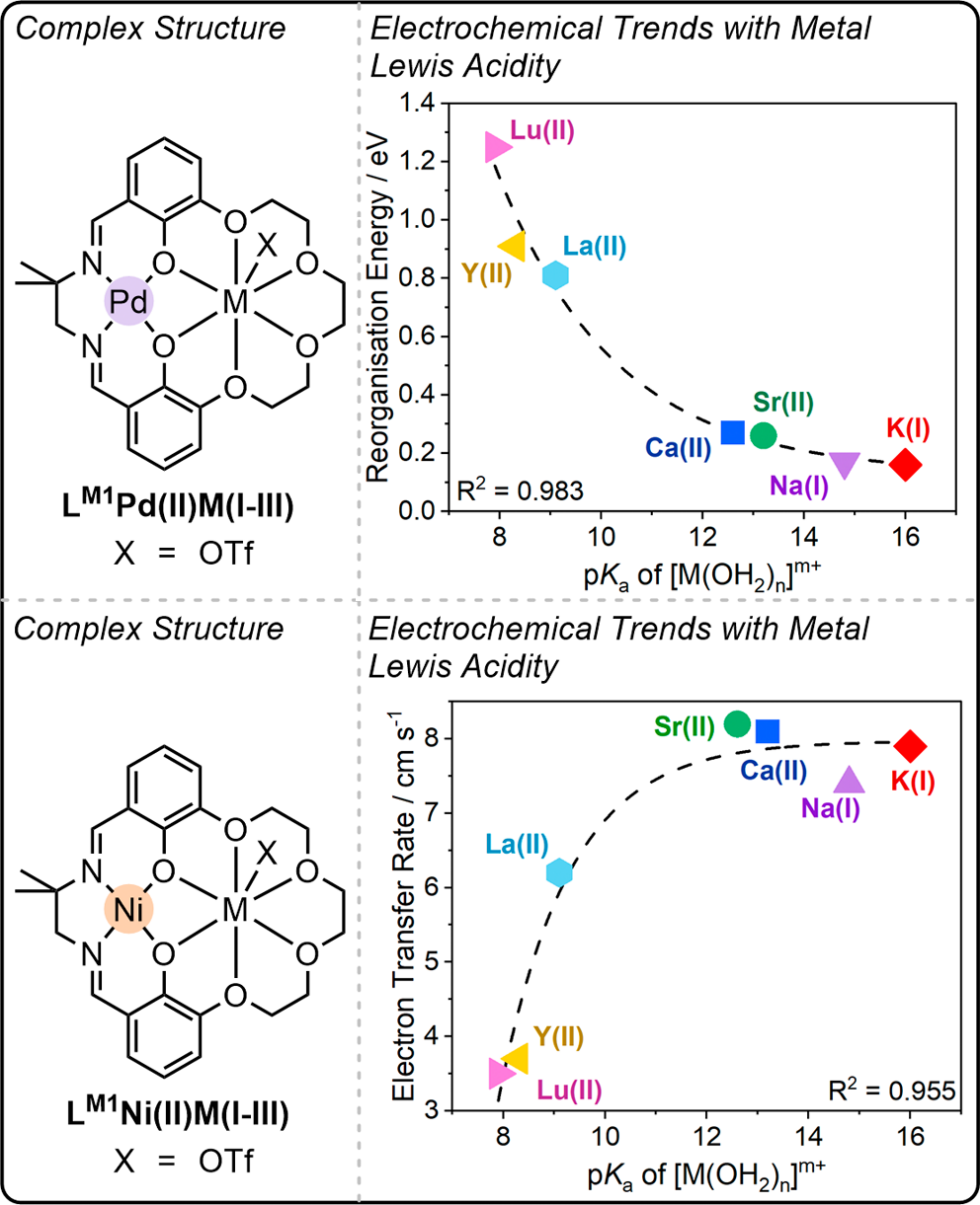
Plots showing exponential trends between the rate of electron transfer (bottom) or reorganization energy (λ; top) with M­(I–III) Lewis acidity (measured by aqua complex p*K*_a_) observed for L^M1^Ni­(II)­M­(I–III) and L^M1^Pd­(II)­M­(I–III).^13,14^ Plots adapted with permission from ref. 13. Copyright 2022 John Wiley and Sons (top) and from ref. 14. Copyright 2024 American Chemical Society (bottom).

Given the chemical irreversibility of the Pd­(II/I) reduction wave, the previously-reported method by Savéant and Costentin was employed to determine the reorganization energies associated with the individual electron transfer processes from the broadening of the voltametric wave for each L^M1^Pd­(II)­M­(I–III) complex.^58^ According to Marcus theory, the reorganization energy is a key kinetic parameter, taking into account both inner and outer sphere effects, which directly relates to the rate of electron transfer.^59^ The rate of electron transfer and reorganization energy are inversely related; faster electron transfer is generally associated with a smaller reorganization energy, and vice versa. It was observed that the reorganization energy , obtained from the voltametric waves, was exponentially dependent on the Lewis acidity of the M­(I–III) cation (Figure 5, top RHS). The most Lewis acidic cation studied, Lu­(III), led to the highest reorganization energy, whereas the least Lewis acidic cation, K­(I), led to the lowest reorganization energy, and hence, the highest rate of electron transfer.

In a subsequent study, the same group investigated the rate of electron transfer in a series of L^M1^Ni­(II)­M­(I–III) complexes, where M­(I–III) = La­(III), Y­(III), Sr­(II), Ca­(II), K­(I) and Na­(I) (Figure 5, bottom).^14^ An increase in the peak-to-peak separation of the Ni­(II/I) redox events was observed in complexes containing stronger Lewis acidic cations, such as La­(III), compared to more weakly Lewis acidic cations, such as Na­(I). Using the method of Nicholson and Shain, electron transfer rates were calculated from the peak-to-peak separations for each L^M1^Ni­(II)­M­(I–III) complex. An exponential increase in the rate of electron transfer (i.e., an exponential decrease in the reorganization energy) was observed with decreasing Lewis acidity (i.e., an increasing metal aqua complex p*K*_a_) of the M­(I–III) cation; K­(I) gave the highest rate of electron transfer (Figure 5, bottom). Notably, complexes containing trivalent cations showed a significantly slower electron transfer rate than those containing mono- or divalent cations. This result supports the previous study on Pd­(II)­M­(I–III) complexes, as a higher electron transfer rate is expected to correlate to a lower reorganization energy, which was previously observed using less Lewis acidic metals (Figure 5, top). A subsequent buried volume analysis, using X-ray diffraction data, of the Ni­(II)­M­(I/II) complexes highlighted that fastest electron transfer was achieved in complexes showing the highest free volume around the Ni­(II) center (e.g., Na­(I), Sr­(II), K­(I)), indicating that steric features may be involved in controlling the rate of electron transfer.^14^ These studies highlight that intermetallic synergy modulates both the thermodynamics of the transition metal complex and the electron transfer kinetics.

Systematic studies of L^M^M­(II)­M­(I–III) and L^A^M­(II)­M­(I–III) complexes provide strong evidence for intermetallic synergy between the two metals in both ligand frameworks. These studies demonstrate that intermetallic synergy causes predictable changes in the thermodynamic and kinetic properties of the metal centers involved. While the result of intermetallic synergy can be quantified through linear and exponential correlations to ground state properties of the non redox active metal cation (Lewis acidity as measured by aqua complex p*K*_a_), its fundamental origins are still rather unclear, with some studies suggesting electric fields may be responsible while others propose the ligands and cations enable inductive effects.

In addition to several studies exploring the fundamental properties of L^M^MM­(I–III) complexes, catalysts belonging to this family have been employed as electrocatalysts in multiple reports over the past 20 years.^57^ In 2016, Soo and co-workers reported an L^A2^Ni­(II) electrocatalyst for hydrogen evolution from seawater, which showed enhanced activities upon the addition of electrolytes containing s-block metal (Figure 6).^60^ It was proposed that the added s-block metal is coordinated within the ligand’s ether pocket, thereby forming an L^A2^Ni­(II)­M­(I/II) catalyst *in situ* (where M­(I) = Li­(I), Na­(I), K­(I)). The Ni­(II)­Li­(I) metal combination was the most active, and its performance was attributed to the higher Lewis acidity of Li­(I) compared to K­(I) and Na­(I).

**Figure 6:**
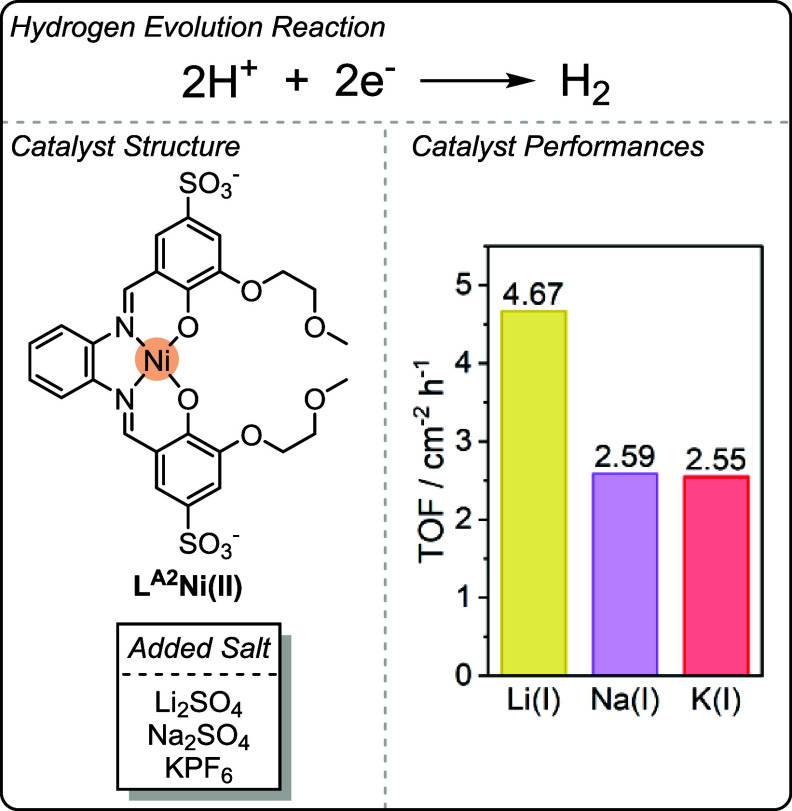
Catalyst structure of the L^A2^Ni­(II)­M­(I) hydrogen evolution catalysts reported by Soo and co-workers.^60^ Bar chart showing the difference in TOF upon addition of Li­(I), Na­(I) and K­(I) salts.^60^ Adapted from ref. 60. Available under a CC-BY 3.0 license. Copyright 2016, Shao et al.

The authors proposed that coordination of the Lewis acidic M­(I) could decrease the reaction overpotential through either electrostatic or inductive effects. This hypothesis agrees with the previously discussed fundamental studies using L^M^M­(II)­M­(I–III) complexes, where linear increases in *E*_1/2_ with increasing M­(I–III) Lewis acidity (i.e., decreasing metal aqua complex p*K*_a_) were observed.^11,12,36,37^ The authors also propose that that intermetallic synergy might also affect catalysis through substrate-directing interactions, which can lead to significant preorganization for the rate-determining step, thereby lowering energetic reaction barriers.

The effect of intermetallic synergy on the electrochemical properties of a transition metal center, and improved substrate interactions exhibited by synergistic catalysts have also been explored by Yang and co-workers.^22,23^ A series of dinuclear Fe­(III)­M­(I/II) and mononuclear Fe­(III) salen complexes were compared for the aerobic oxidation of C–H bonds (Figure 7).^23^ Specifically, LFe­(III), L^M2^Fe­(III)­K­(I) and L^M2^Fe­(III)­Ba­(II) were tested for the oxidation of cyclohexene (0.5 M cyclohexene, CH_3_CN, 1 bar O_2_, 24 h) forming 2-cyclohexen-1-ol and 2-cyclohexenone. The monometallic salen Fe­(III) catalyst (LFe­(III)) was completely inactive and required the addition of a strongly electron-withdrawing NO_2_ substituent for the catalyst to show any turnover. This substituent led to a strongly oxidizing iron center (*E*_1/2_Fe­(III/II) > - 0.32 V). In contrast, upon coordination of K­(I) or Ba­(II) in the unfunctionalized L^M2^Fe­(III) complexes, activities were observed at significantly less oxidizing potentials (Figure 7, bottom).

**Figure 7:**
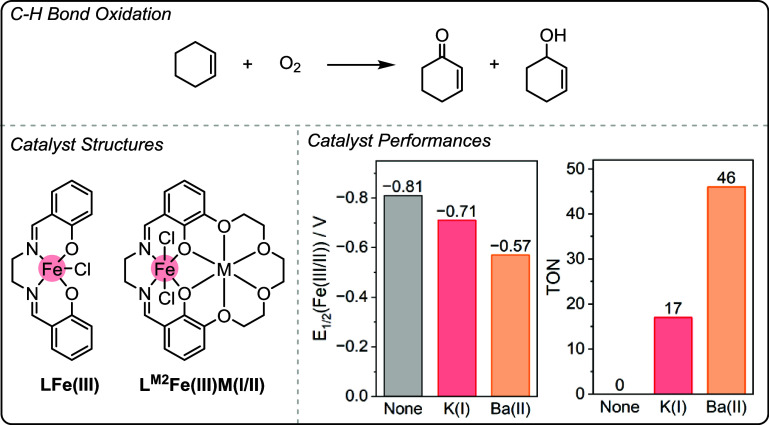
Top: Aerobic oxidation of C–H bonds in cyclohexene. Bottom LHS: LFe­(III) and L^M2^Fe­(III)­M­(I/II) catalyst structures reported by Yang and co-workers for the aerobic oxidation of C–H bonds in cyclohexene. Bottom RHS: Bar charts showing the inversed relationship between *E*_1/2_(Fe­(III/II)) and catalytic activity (TON).^23^ Adapted from ref. 23. Available under a CC-BY 3.0 license. Copyright 2018 Chantarojsiri et al.

The proposed catalytic cycle follows a mechanism previously proposed by Gray, Labinger and co-workers for other Fe­(III) complexes, in which cyclohexene hydroperoxide is formed and then decomposes to produce the ketone and alcohol products (Figure S8).^61–63^ The coordination of the second metal in close proximity to the iron center was proposed to promote catalytic activity in the rate-limiting step, which is the oxidation of an organic hydroperoxide, by facilitating intramolecular electron transfer, for example through coordination of the lone pairs of hydroperoxides to M­(I/II). The proposed effect of M­(I/II) coordination on the rate of electron transfer is consistent with the recent studies conducted by Blakemore and co-workers, where more weakly Lewis acidic M­(I/II) metals show faster rate of electron transfer in L^M^MM­(I–III) complexes.^13,14^

Using the Fe­(III)­M­(I/II) heterodinuclear catalysts in the oxidation of cyclohexene also resulted in a lower rate of catalyst deactivation to an inactive iron-μ_2_-oxo species (Figure S8). The half-lives of the aerobic oxidation of L^M2^Fe­(III)­K­(I) and L^M2^Fe­(III)­Ba­(II) were 18 and 37 s, respectively. The slower rate of oxidation observed for the L^M2^Fe­(III)­Ba­(II) is consistent with the overall higher activity and the more positive *E*_1/2_Fe­(III/II). This study illustrates that intermetallic synergy both enhances and accelerates the desired forward reaction in electrocatalysis and may help to suppress undesired side reactions.

Yang and co-workers have further studied and applied intermetallic synergy in L^M2^[Mn­(V)­N]­M­(I–III) complexes (where M­(I–III) = Na­(I), K­(I), Ba­(II), Sr­(II), La­(III), Eu­(III); Figure 8).^22,42^ High-valent metal oxo and nitride complexes are relevant for bioinspired oxidation catalysis.^64,65^ Molecular high valent-nitrido complexes may be useful model complexes to gain insight into the oxidation mechanisms, as well as undesired side-reactions such as bimolecular coupling to form molecular N_2_ (Figure S9). In 2018, Yang and co-workers proposed that the s-block metals in the L^M2^[MnN]­M­(I/II) complexes help to suppress this undesired coupling reaction.^22^ The Mn­(VI/V) *E*_1/2_ was shifted positively with increasing M­(I/II) cation charge (189 mV per charge; Figure S10). It had previously been observed that a positive shift of the Mn­(V/VI) oxidation potential of substituted MnN salen complexes led to an increase in the rate of N–N coupling reaction to form molecular N_2_ and Mn­(III) complexes.^22^ However, for L^M2^[MnN]­M­(I/II), where M­(I/II) = K­(I), Na­(I), Ca­(II) or Sr­(II), the opposite trend was observed: the rate of the bimolecular coupling reaction between two [Mn­(VI)­N]^+^ complexes, obtained by an electrochemical, one electron oxidation of the starting [Mn­(V)­N] complexes, decreased with positively shifted oxidation potentials (Figure 8, bottom LHS). Specifically, it was observed that the semilogarithmic plot of the rate of bimolecular coupling vs *E*_1/2_ Mn­(VI/V) was linear, revealing a linear free energy relationship. It was proposed that an increased electrostatic repulsion with increasing cation charge leads to the different rates of bimolecular coupling observed for different L^M2^ Mn­(VI)­M­(I/II) catalysts (Figure S10).

**Figure 8:**
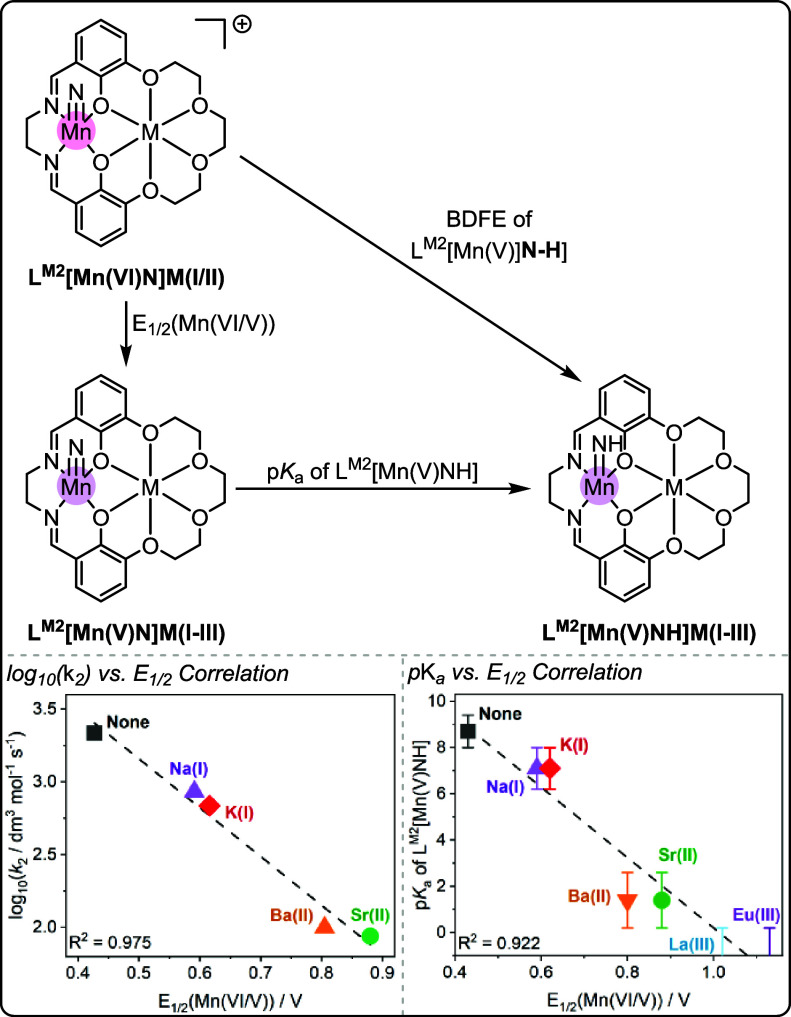
Top: Overview of the L^M2^Mn­(VI/V)­M­(I–III) complexes and how they are interrelated, using data from papers by Yang and co-workers.^22,42^ Bottom: Correlations between the rate of undesired bimolecular coupling to form N_2_ and Mn­(V) imido complex p*K*_a_ with Mn­(VI/V) *E*_1/2_.^22,42^ Plot LHS (bottom) reproduced with permission from ref. 22. Copyright 2018 John Wiley and Sons. Mechanism and plot RHS (bottom) re-produced from ref. 42. Copyright 2022 American Chemical Society.

High-valent Mn-nitrido complexes have also been explored for the activation of nitrogen–hydrogen bonds. As such, the bond dissociation free energies of the reactant Mn-nitrido complexes ([Mn­(VI)≡N]^+^) and product complexes ([Mn­(V)=NH]^+^) are important parameters. The bond dissociation free energy is expected to be dependent on both the Mn­(VI/V) redox potential, and the p*K*_a_ of the imido complex, which describes the protonation of the nitrido complex to form the transient imido ([Mn­(V)=NH]^+^) complex (Figure 8).

It was observed that the redox potentials for the series of L^M2^[Mn­(VI)­N]­M­(I/II/III) span >700 mV, and the p*K*_a_ of the corresponding [Mn­(V)=NH]^+^ complexes span >9 p*K*_a_ units (Figure 8, bottom RHS and Figure S11).^22,42^ It was proposed that the redox potential and the p*K*_a_ of each Mn­(V)N complex exhibit a compensatory relationship, leading to an overall unchanged bond dissociation free energy. This suggests that intermetallic synergy can modulate the electronic properties of a transition metal center, without affecting its ability to undergo hydrogen atom transfer.

Overall, intermetallic synergy, achieved through the installation of proximal f- or s-block metals in transition metal complexes, allows for precise tuning of their electrocatalytic activity. Building on a series of carefully conducted studies on the fundamental effects of intermetallic synergy between nonredox active cations and transition metals, more recent studies on the catalytic applications of these complexes have shown that intermetallic synergy can also contribute by directing substrates or suppressing side reactions.

## Ring-Opening Copolymerization

There are a range of different catalysts reported for the ring-opening copolymerization (ROCOP) of epoxides with CO_2_ to make polycarbonates, or of epoxides with cyclic anhydrides to make polyesters (Figure 9).^9,66–68^ Using these catalytic polymerizations, there are opportunities to reduce greenhouse gas emissions compared to current industrial manufacturing methods. For example, life cycle assessments have established significant reductions by using carbon dioxide directly to make polycarbonates.^67,69–74^ Furthermore, several epoxides and cyclic anhydrides are bioderived which may also help reduce embedded greenhouse gas emissions associated with polymer manufacturing. For example, poly­(propene carbonate) and poly­(propene ether carbonate) can be used as polymer electrolytes, surfactants, polyurethane foams, coatings, elastomers, adhesives and sealants.^72,75–77^

**Figure 9:**
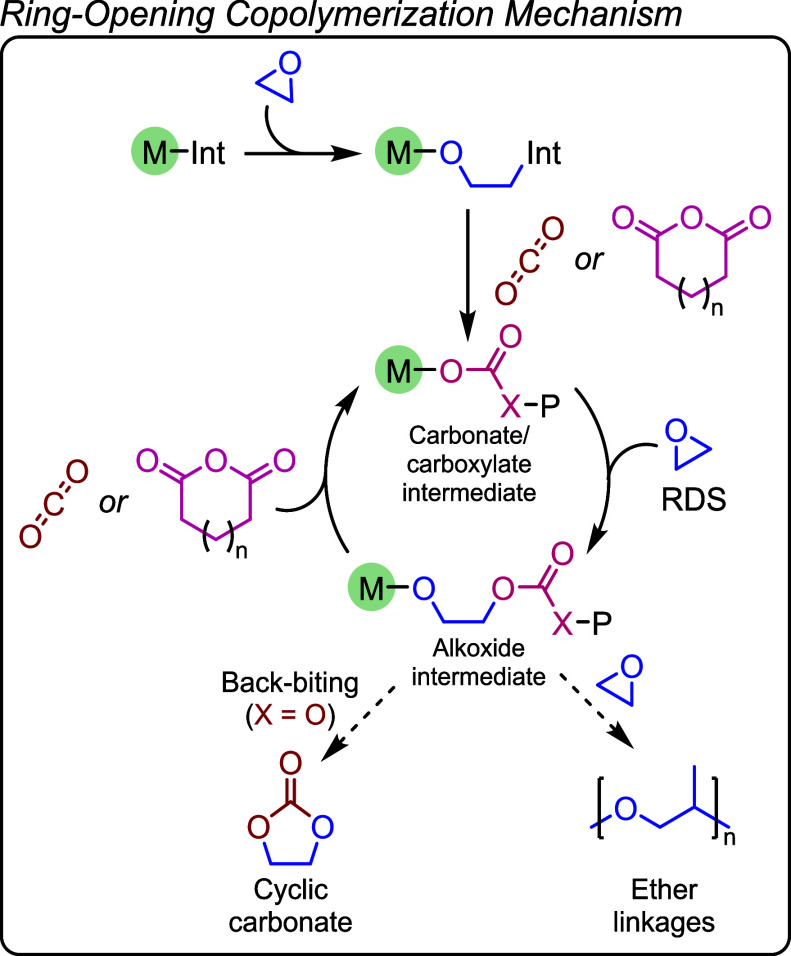
General mechanism for the ring-opening copolymerization of epoxides with CO_2_ (X = O) or cyclic anhydrides (X = C) where Int = initiator, *P* = polymer, and RDS = rate-determining step.

In 2015, we reported the first synergistic, heterodinuclear catalysts for the ROCOP of epoxides and CO_2_, incorporating Zn­(II) and Mg­(II) in a diphenolate tetra-amine-type ligand.^78–80^ Using this family of catalysts, detailed investigations into synergistic metal combinations as well as kinetic, computational and mechanistic studies using the most effective Co­(II)­Mg­(II) catalyst uncovered a basis for catalytic synergy.^80^ Since this report, a number of high performance synergistic heterometallic catalysts have been reported for the ROCOP of epoxides and CO_2_, and of epoxides with anhydrides as well as for the related ring-opening polymerization (ROP) of cyclic esters to produce polyesters.^3,9,66,67,69–74,81,82^

For example, we and others have reported catalysts deliberately selected to target transition metal (M­(III)) and s-block metal (M­(I/II)) combinations, featuring L^M^ and L^A^ ligands. It is this second generation of heterodinuclear catalysts are the focus of this perspective, as they share similar metal combinations (and oxidation states) and show closely related quantified structure-performance relationships, albeit for a different form of catalysis to the previously discussed electrocatalysts.

The ROCOP of epoxides with CO_2_ or with cyclic anhydrides occur via related mechanisms (Figure 9). Initiation generally involves coordination of the epoxide at one of the metal centers of the catalyst and its ring-opening is initiated by the catalyst-coordinated nucleophile, e.g. carboxylate or halide coligands. The first ring-opening step generates a catalyst-alkoxide intermediate which inserts carbon dioxide to form a catalyst-carbonate intermediate. Propagation involves cycling between the alkoxide and carbonate intermediates by sequential insertion of epoxide and carbon dioxide, respectively.^66–68^ The ROCOP of epoxides and anhydrides operates through an analogous mechanism, with the alkoxide intermediate ring-opening the cyclic anhydride to form a carboxylate intermediate. ROCOP catalysis is generally a living polymerization, meaning that the loading of catalyst and protic additives control the polymer molar mass and end group chemistry – both features are essential for further applications of the polymers.^83,84^

There are two side reactions which should be suppressed. In both epoxide/CO_2_ and epoxide/anhydride ROCOP, sequential epoxide enchainment is feasible to form ether linkages. This reduces carbonate or carboxylate linkage selectivity, and carbon dioxide uptake in the case of polycarbonates. In epoxide/CO_2_ ROCOP, intramolecular cyclization (“backbiting”) is also feasible and forms the thermodynamically stable five-membered ring cyclic carbonate.

In 2020, we reported the first series of heterodinuclear catalysts active for PO/CO_2_ ROCOP; the catalysts featured a macrocyclic L^M2^ ligand and Co­(III)­M­(I) metal combination (M­(I) = Na­(I), K­(I), Rb­(I), Cs­(I); Figure 10).^86^ The most active catalyst was L^M2^Co­(III)­K­(I), which was able to operate at a low loading, moderate temperature and showed a high turnover frequency of up to 800 h^–1^. It also maintained a high selectivity of 93% for polymer over cyclic carbonate, and was quantitatively selective for carbonate linkages (1:20:4000 [catalyst]_0_:[diol]_0_:[PO]_0_, 70 °C, 30 bar CO_2_; Figure 10, top; Figure 11, RHS).^86^ Kinetics investigations revealed an overall second order rate law which included first-order dependencies in both [PO] and [catalyst]. There was a zero-order dependence on CO_2_ pressure between 10 and 40 bar. The relative transition state free energy barriers were measured, using Eyring analyses, for both polymerization and cyclic carbonate byproduct formation. Computational investigations of the catalytic cycle supported a dinuclear metalate mechanism where the rate-determining step was metal-carbonate attack on the coordinated propene oxide. The calculations suggested distinct roles for Co­(III) and K­(I) in the rate-determining step, with Co­(III) coordinating and activating the propene oxide, and ring-opening occurring by attack from the K­(I)-carbonate intermediate (Figure 10, bottom).^85,86^ There was a very good agreement between the calculated and experimental transition state Gibbs free energy barriers, which supported the proposed heterodinuclear polymerization mechanism and gave insight into the basis for the synergistic cooperativity.

**Figure 10:**
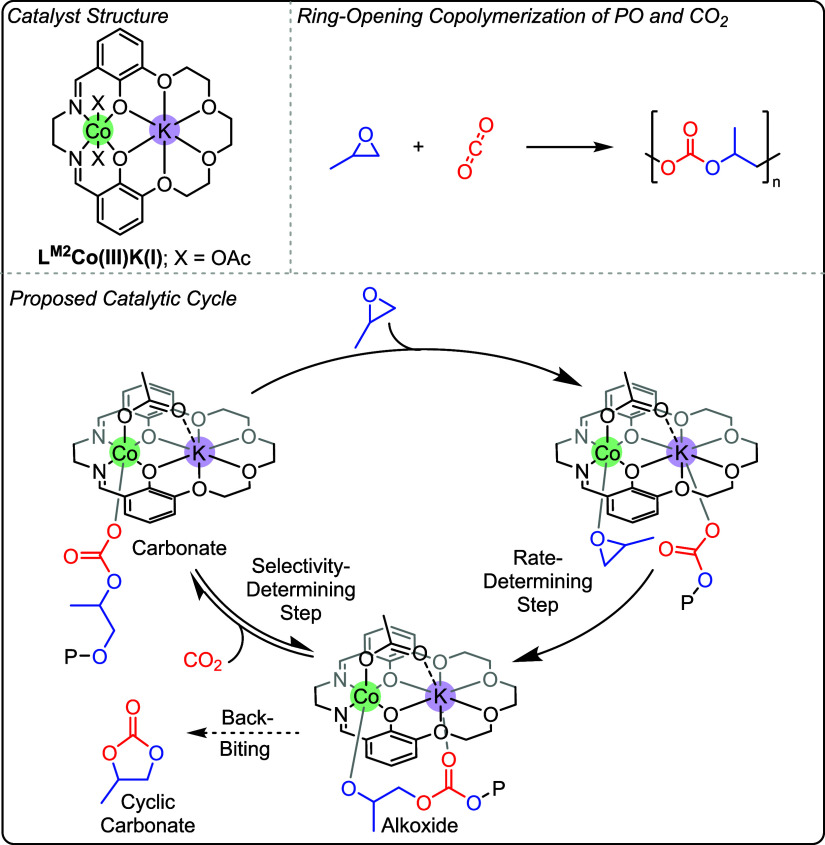
Top: Structure of L^M2^Co­(III)­K­(I) catalyst for the ring-opening copolymerization of propene oxide and CO_2_. Bottom: Proposed catalytic cycle, informed by kinetic and computational experiments, from reports by Williams and co-workers.^85,86^

**Figure 11:**
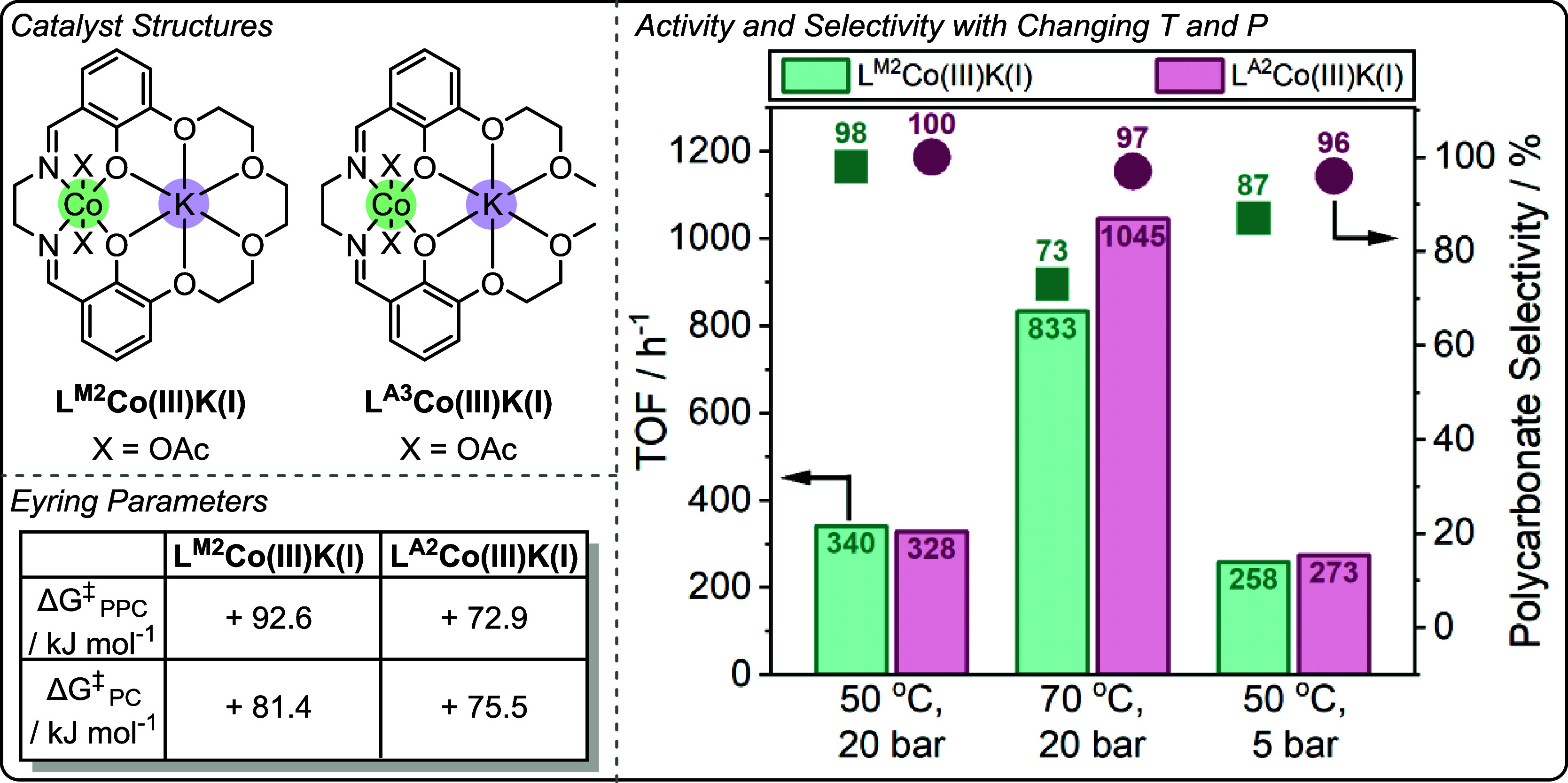
Comparison of macrocyclic L^M2^Co­(III)­K­(I) and acyclic L^A3^Co­(III)­K­(I) catalysts for the ring-opening polymerization of propene oxide and CO_2_ using data reported by Williams and co-workers.^86,87^ Adapted from refs. 86, 87. Copyright 2020 and 2024 American Chemical Society.

In 2024, the performance of the L^M2^Co­(III)­K­(I) catalyst for PO/CO_2_ ROCOP was improved by using a more flexible, acyclic L^A3^ ligand system. L^A3^Co­(III)­K­(I) shows both a higher activity (TOF = 1,045 h^–1^) and polymer selectivity (97%) compared to L^M2^Co­(III)­K­(I) under the same conditions (TOF = 833 h^–1^, and polymer selectivity = 63%, 70 °C, 20 bar; Figure 11).^87^ Comparison of the transition state barriers, determined by Eyring analysis, for the L^M2^Co­(III)­K­(I) and L^A3^Co­(III)­K­(I) catalysts indicates that the more flexible L^A3^ framework leads to a lower transition state energy (Δ*G*_PPC_^⧧^ = +72.9 ± 5.1 kJ mol^–1^ vs Δ*G*_PPC_^⧧^ = +92.6 kJ mol^–1^, Figure 11).

A detailed kinetic analysis of the L^A3^Co­(III)­K­(I) catalyst revealed that it was active from 2 to 35 bar CO_2_ pressure. At higher pressures (12–35 bar), the rate law was overall second order, with a first order dependence on [PO] and [catalyst], as was observed for L^M2^Co­(III)­K­(I). Between 2 and 12 bar CO_2_ pressures, a third order rate law which was also dependent on [CO_2_] was uncovered. The data are interpreted by a catalytic cycle involving a carbon dioxide insertion equilibrium between iso-energetic catalyst-alkoxide and catalyst-carbonate intermediates. The CO_2_ insertion step is a pre-rate determining equilibrium and is responsible for the high selectivity for polymer (vs cyclic carbonate). Accordingly, at low CO_2_ pressures, the extent of the pre-equilibrium controls the concentration of the key catalyst-carbonate intermediate. At and above the key threshold pressure, ∼ 12 bar, the pre-equilibrium is saturated and the maximum concentration of carbonate intermediate is achieved resulting in rates which become independent of carbon dioxide pressure.^87^

It is proposed that both L^M2^Co­(III)­K­(I) and L^A3^Co­(III)­K­(I) show such reversible carbon dioxide insertion chemistry, in the pre-rate limiting step and that selectivity is controlled by the extent of this insertion equilibrium. It is important to understand the kinetics and proposed catalytic cycles for these classes of catalysts since they are directly implicated in the interpretation of the quantified structure-performance relationships.

In 2023, a systematic series of L^M2^Co­(III)­M­(I/II) catalysts, where M­(I/II) = Na­(I), K­(I), Ca­(II), Sr­(II), Ba­(II), were investigated for three different polymerizations: PO/CO_2_ ROCOP, PO/phthalic anhydride (PA) ROCOP, and rac-lactide (LA) ROP.^24^ The study revealed clear, quantified correlations between both catalytic activity and selectivity with s-block metal Lewis acidity, as measured by the metal aqua complex p*K*_a_, for all three polymerizations (Figure 12).^24,47^ There were no correlations between catalyst performances and the strength of M­(I/II) coordination (estimated by binding constant of the s-block cations to 18-crown-6) or to the M­(I/II) ionic radius (Figure S12).^45,88^ For the ROCOP of PO and CO_2_, the catalytic activity increases exponentially with decreasing s-block metal Lewis acidity and increasing M­(I/II) Lewis acidity (measured by the metal aqua complex p*K*_a_), with performance peaking at Co­(III)­K­(I).

**Figure 12:**
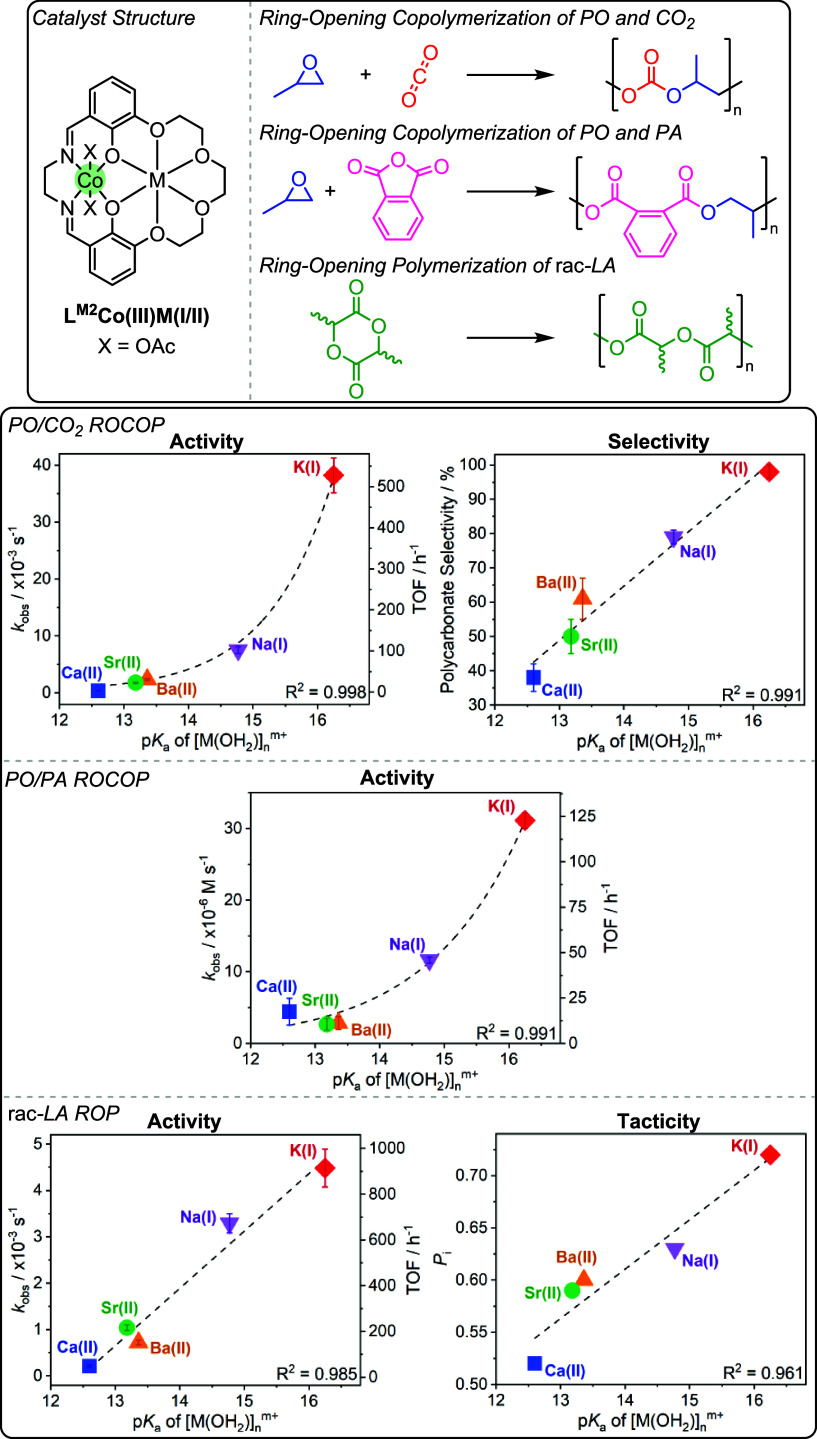
Top: Structure of L^M2^Co­(III)­M­(I/II) catalysts for the ring-opening copolymerizations of propene oxide with CO_2_, and propene oxide with phthalic anhydride, and the ring-opening polymerization of racemic lactide, from a report by Williams and co-workers.^24^ Bottom: Structure-performance correlations for each of the tested polymerizations against s-block metal Lewis acidity.^24^ Reproduced from ref. 24. Available under a CC-BY 4.0 license. Copyright 2023 Fiorentini et al.

The exponential correlation is rationalized with reference to the dinuclear metalate mechanism, and the functions for the two metals, as proposed by DFT investigations. As such, the s-block metal–carbonate species is the key nucleophile and is proposed to become more labile when coordinated to progressively more weakly Lewis acidic metals. It is the enhancement of catalyst-carbonate nucleophilicity which is proposed to lower the enthalpy barrier to epoxide ring-opening and increase catalytic activity. Plots of the polycarbonate selectivity showed a linear increase with decreasing s-block metal M­(I/II) Lewis acidity (i.e., increasing metal aqua complex p*K*_a_). This was rationalized by the chemistry of the key Co­(III)-alkoxide intermediate and the effect of decreasing s-block metal Lewis acidity to disfavor intramolecular backbiting. Examining the structure–activity data for PO/PA ROCOP showed the same exponential increase of catalyst activity with decreasing s-block metal M­(I/II) Lewis acidity (metal aqua complex p*K*_a_), again reaching a maximum with the Co­(III)­K­(I) catalyst.

This trend is consistent with the proposed catalytic cycle in which the rate-determining step involves M­(I/II)-carboxylate nucleophile attack on a Co­(III)-PO adduct. Once again, we proposed that the weaker the Lewis acidity of the M­(I/II) center, the more labile the carboxylate species, the lower the enthalpy barrier and the faster the catalysis.

The final polymerization examined was *rac*-LA ROP; examining the series of catalysts revealed a linear increase in polymerization activity and stereocontrol, specifically isotacticity (*P*_i_), with decreasing s-block M­(I/II) metal Lewis acidity (i.e., increasing metal aqua complex p*K*_a_). The structure–activity relationship was fully consistent with the prior trends since *rac*-LA ROP involves a rate-determining step in which a catalyst-alkoxide nucleophile attacks and ring-opens a catalyst-activated LA species.

An unexpected revelation from the structure-performance investigations using these three different catalytic processes is that the nature of the correlations, exponential or linear, may inform upon the nature of the chemistry of the key reaction intermediate. Carbonate and carboxylate intermediates seemingly result in exponential correlations, while alkoxide intermediates resulted in linear correlations. In each case, the logarithmic plots of the exponential correlations are linear, indicating that the observed structure–activity relationships are linear free energy relationships (Figure S13). These quantified structure–activity/selectivity correlations provide a very interesting opportunity to identify the key catalytic intermediates and may aid future catalyst design.

This year, we published a series of structure–activity correlations using the same L^M2^Co­(III)­M­(I/II) catalysts for cyclohexene oxide (CHO)/CO_2_ ROCOP (Figure 13).^89^ The copolymerization of cyclohexene oxide is important since it is widely used in academic laboratories to benchmark catalyst performances. The resulting polymer, poly­(cyclohexene carbonate), shows promise as an engineering plastic and can be effectively chemically recycled to CHO/CO_2_ mixtures after use.^90–92^ The structure-performance relationships for CHO/CO_2_ ROCOP, using 20 bar CO_2_ pressure, showed the same exponential increase in activity with decreasing Lewis acidity of the s-block metals, as observed for PO/CO_2_ ROCOP (Figure 13 top). This is fully consistent with the proposed dinuclear metalate mechanism, again, quantifying the reactivity of the s-block metal carbonate intermediate.

**Figure 13:**
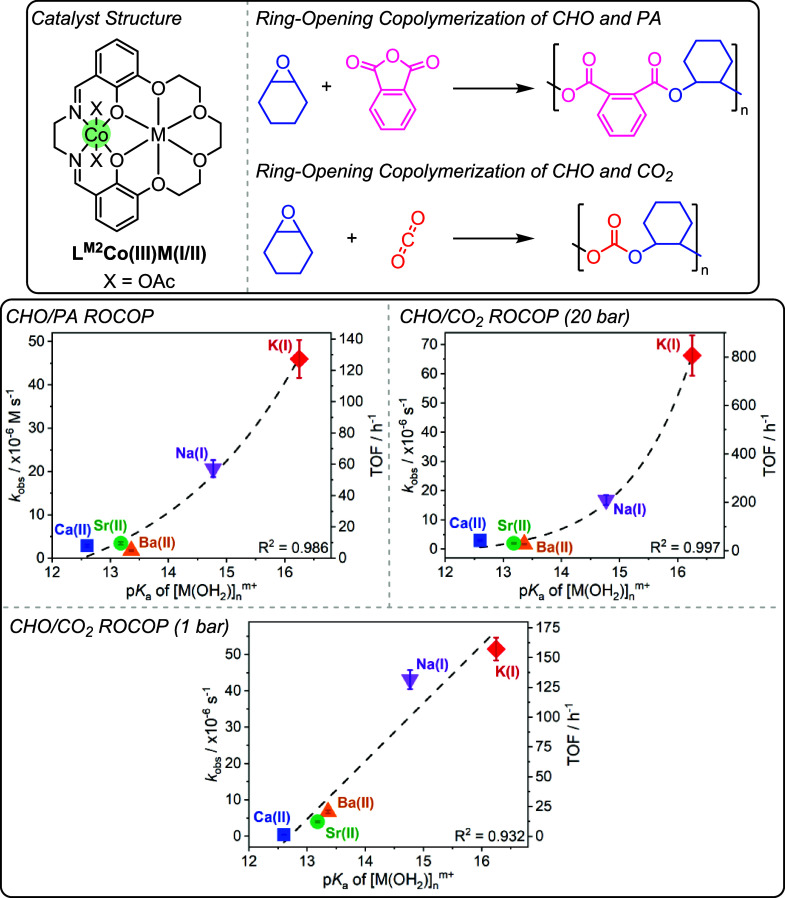
Top: Structure of L^M2^Co­(III)­M­(I/II) catalysts for the ring-opening copolymerizations of cyclohexene oxide with phthalic anhydride, and cyclohexene oxide with CO_2_, as reported by Williams and co-workers.^89^ Bottom: Structure-performance correlations for each of the tested polymerizations against s-block metal Lewis acidity.^89^ Reproduced from ref. 89. Available under a CC-BY 4.0 license. Copyright 2025 Butler et al.

However, when decreasing the CO_2_ pressure to 1 bar, it was observed that catalyst activity increased linearly with decreasing M­(I/II) Lewis acidity, instead of the previous exponential correlations (Figure 13, bottom). In line with the previous structure-performance study on PO/CO_2_ ROCOP, in which linear correlations had been observed for selectivity vs p*K*_a_, the linear correlation at 1 bar was interpreted as being indicative of an alkoxide as the key reaction intermediate. The change in the nature of the correlation, and hence key reaction intermediate from exponential (indicative of a carbonate intermediate) at 20 bar to linear (indicative of an alkoxide intermediate) at 1 bar, was rationalized by understanding the chemistry of carbon dioxide insertion in these catalytic cycles. As CO_2_ pressure is decreased from 20 to 1 bar, the CO_2_ insertion equilibrium should shift from the carbonate intermediate toward the alkoxide intermediate. It is proposed that the linear increase in activity vs decreasing Lewis acidity signals that under the lowest pressures, the carbon dioxide insertion equilibrium is shifted further towards the alkoxide intermediate, such that it influences rate. In contrast, at high CO_2_ pressures, the equilibrium is proposed to be shifted toward the carbonate intermediate and hence, does not affect the rate of reaction (Figure S14).

Finally, CHO/PA ROCOP was also investigated using the same series of catalysts and showed an exponential increase in activity vs decreasing Lewis acidity of the M­(I/II) center.^89^ For the two exponential correlations, linear free energy relationships were verified using double logarithmic plots (Figure S15). The investigations into both PO/heteroallene and CHO/heteroallene ROCOP catalysts showed generally applicable quantified structure–activity and structure-selectivity relationships. These linear free energy relationships help rationalize differences in catalytic performance, substantiate the dinuclear metalate polymerization mechanism, and may help to inform future catalyst design.

In 2023, we reported a series of L^M^Co­(III)­K­(I) catalysts with modified ligand phenyleneimine linker chemistries, particularly focused on systematic changes to the substituents on the phenylene groups (Figure 14).^25^ It was noted that the Co­(III/II) redox potentials correlate linearly with catalytic activity and selectivity for the ROCOP of PO and CO_2_.

**Figure 14:**
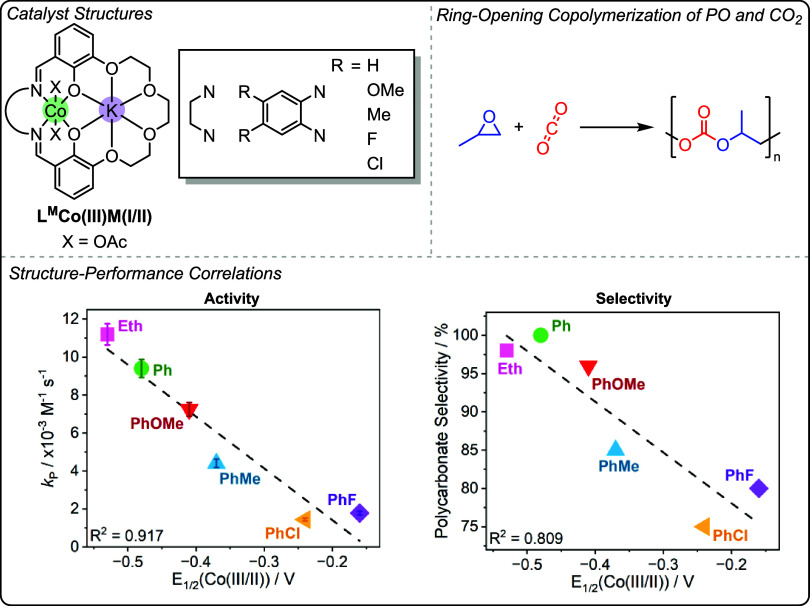
Top: Series of L^M^Co­(III)­K­(I) catalysts for the ring-opening copolymerization of propene oxide and CO_2_ with changing imine linker to change the electronics of the Co­(III) center, as reported by Williams and co-workers.^25^ Bottom: Correlations between *E*_1/2_(Co­(III/II) (used as a measure of Co­(III) electron density) and activity and selectivity.^25^ Reproduced from ref. 25. Available under a CC-BY 4.0 license. Copyright 2023 Lindeboom et al.

The Co­(III) centers with more negative reduction potentials, perhaps correlating with higher electron densities, showed the fastest rates and highest polymer selectivity in the catalysis.^25^ The linear structure–activity data was rationalized by the same dinuclear metalate mechanism, with electron-rich Co­(III) centers resulting in slightly destabilized alkoxide and/or epoxide-coordination intermediates, perhaps biasing the CO_2_ insertion equilibrium toward the carbonate intermediate, favoring fast and selective polycarbonate formation. This structure–activity study emphasizes the need to carefully balance the ligand electronics in these Co­(III)­K­(I) catalysts, to mediate the relative energies of the key intermediates in the catalytic cycle and promote polymerization.^25^

The importance of optimizing the M­(III) center electron density was further highlighted in a 2024 study comparing the epoxide/heteroallene ROCOP activity of a series of three catalysts featuring different M­(III) sites but otherwise identical chemistries, L^A3^ M­(III)­K­(I) (where M­(III) = Co­(III), Al­(III), or Fe­(III), Figure S16).^93^ For PO/CO_2_ ROCOP and CHO/CO_2_ ROCOP, the Co­(III)­K­(I) catalyst was the best: it outperformed both the Al­(III) and Fe­(III) catalysts (Figure S16). Investigations to try to rationalize the differences in catalytic performances between the three M­(III) combinations used spectroscopy and X-ray diffraction characterization of the ground state complex structures (Figure S16). These data were interpreted by considering the metals as Lewis acids, with the Al­(III) and Fe­(III) centers being less Lewis acidic than the analogous Co­(III) center when bound in the same L^A2^ ligand framework with K­(I). It was hypothesized that the activity of the catalysts correlates with the Lewis acidity of the M­(III) center. The less Lewis acidic Fe­(III) and Al­(III) centers may perhaps lead to a weaker polarization of the epoxide C–O bond, and hence, a lower rate of epoxide ring opening compared to the Co­(III) catalyst. This highlights that, while weakly Lewis acidic (more electron rich) Co­(III) centers favor rapid and selective polymerization, there is a limit to the rate- and selectivity-enhancement with increasing electron-density.^93^

In 2024, we reported upon the influences of intermetallic separation in a series of Co­(III)­K­(I) catalysts for PO/CO_2_ and PO/PA ROCOP.^94^ The study included two heteronuclear Co­(III)­K­(I) catalysts with well-defined, either narrow or wide, intermetallic Co­(III)–K­(I) separation. These were compared against a catalyst mixture of variable intermetallic separation (Figure S17).^94^ The catalyst with wide intermetallic separation (Co – K = 8.06 Å, determined by single crystal X-ray diffraction) showed field-leading activity for PO/PA ROCOP with a TOF of 1,686 h^–1^ (1:20:400:1000 [catalyst]_0_:[diol]_0_:[PA]_0_:[PO]_0_, 60 °C, neat), but is completely inactive for PO/CO_2_ ROCOP. In contrast, the catalyst with short intermetallic separation (L^M3^Co­(III)­K­(I); Co – K = 3.59 Å by X-ray diffraction) is an excellent catalyst for PO/CO_2_ ROCOP with a TOF of 389 h^–1^ and polymer selectivity >99%, (1:20:4000 [catalyst]_0_:[diol]_0_:[PO]_0_, 50 °C, 20 bar CO_2_, neat) but is much less active for PO/PA ROCOP with a TOF = 231 h^–1^ (1:20:400:1000 [catalyst]_0_:[diol]_0_:[PA]_0_:[PO]_0_, 60 °C, neat). The catalyst mixture performs well in both polymerizations at sufficiently high catalyst loadings but is not effective under the desired high catalyst dilution conditions. Based on kinetic analyses, it was proposed that the wider separation catalyst and the shorter separation catalyst operate via different mechanisms in the two PO/heteroallene ROCOPs. In both mechanisms, the K­(I) center is hypothesized to bind the propagating carboxylate/carbonate chain, however, for PO/PA ROCOP the wider separation catalyst may benefit from a dissociated, free carboxylate nucleophile. In contrast, for PO/CO_2_ ROCOP such dissociation is proposed to be a disadvantage for the reaction selectivity, and hence, the best catalyst is the narrow separation species where the K-carboxylate is coordinated throughout the cycle.^94^

We have also reported synergistic Al­(III)­M­(I) catalysts for epoxide/anhydride ROCOP.^9,95–97^ In 2021, we reported a series of L^A3^Al­(III)­M­(I) catalysts for CHO/PA ROCOP (Figure S18).^95^ The best catalysts contained relatively weak Lewis acids K­(I) and Rb­(I), which outperformed catalysts in corporating Na­(I) and Cs­(I), further underlining the importance of s-block metal choice (L^A3^Al­(III)­M­(I): TOF = 1072 h^–1^ (Al­(III)­K­(I)), 1136 h^–1^ (Al­(III)­Rb­(I)) 1:400:2000 > 99% polyester linkages, [catalyst]_0_:[PA]_0_:[CHO]_0_, 100 °C, neat; Figure S18).^95^ In line with our initial reports on the importance of the s-block metal in synergistic Al­(III)­M­(I) catalysts, similar observations were made by Plajer and co-workers in 2024, using related L^A5^Al­(III)­M­(I–III) catalysts for the ROCOP of CHO with polythioanhydride (PTA; Figure S19).^98^ In that ROCOP catalysis, the Al­(III)­Rb­(I) was the highest performing metal combination, yielding perfectly alternating poly­(ester-thioesters) with good activity (TOF = 638 h^–1^, 1:250:1000, [catalyst]_0_:[PTA]_0_:[CHO]_0_, 80 °C, neat).

In 2024, we investigated the role of the Al­(III) coordination environment.^97^ An increase in the activity of L^A^Al­(III)­K­(I) catalysts by two orders of magnitude was achieved when changing the imine backbone linker from phenylene to dimethyl-substituted propylene (TOF = 46 vs 1070 h^–1^, > 99% polyester linkages, 1:400:2000, [catalyst]_0_:[PA]_0_:[CHO]_0_, 100 °C, neat; Figure S20).^97^ Rate differences were tentatively attributed to changes to the ligand conformations and coordination environment at Al­(III), emphasizing the importance of the overall complex structure in facilitating synergistic rate enhancements.

In 2018, Okuda, Mashima and co-workers reported a series of tetranuclear L^M4^Zn­(II)_3_Ln­(III) catalysts (where Ln­(III) = Dy­(III), Gd­(III), Eu­(III), Sm­(III), Nd­(III), Pr­(III), Ce­(III) or La­(III)) for CHO/CO_2_ ROCOP (Figure 15).^38^ The ligand features three phenoxy-imine Zn­(II) subunits, which form a macrocyclic structure with the Ln­(III) coordinated by six phenoxy donors. L^M4^Zn­(II)_3_Ce­(III) was the most active catalyst of the series, and a cooperative mechanism was proposed where Ce­(III) binds the epoxide which is attacked by a Zn­(II)-carbonate nucleophile (TOF = 370 h^–1^, polymer selectivity >99%, carbonate linkage content >99%, [catalyst]_0_:[CHO]_0_ 1:2000, 100 °C, 10 bar CO_2_). In the original publication there were no reported plots showing structure-performance correlations, however, when we plotted the reported catalytic activity against lanthanide Lewis acidity (measured by the metal aqua complex p*K*_a_), a correlation is apparent; the highest activity was achieved using Ce­(III) (Figure 15, bottom RHS).^38^ This correlation could either be described by a linear fit, considering La­(III) as an outlier, or by a volcano-type correlation, including La­(III). La­(III) could be an outlier due to its ability to undergo ligand exchange reactions. The observed correlation, independent of its shape, may be rationalized by considering two requirements for the lanthanide center: to be a Lewis acid able to activate the epoxide, yet to provide a labile lanthanide-alkoxide bond to furnish the Zn-carbonate nucleophile.

**Figure 15:**
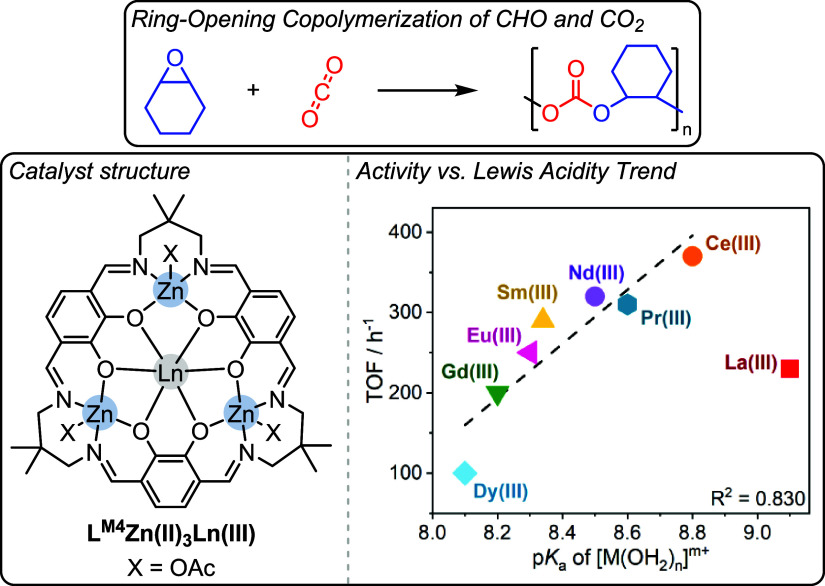
Tetranuclear L^M4^Zn­(II)_3_Ln­(III) catalysts for the ring-opening copolymerization of cyclohexene oxide and CO_2_, as reported by Mashima and Okuda and co-workers.^38^ Bottom RHS: Plot of catalyst activity against lanthanide Lewis acidity, which we constructed by plotting activity data reported in the original article with lanthanide aqua complex p*K*_a_ values.^38^ Adapted with permission from ref. 38. Copyright 2018 John Wiley and Sons.

In 2020, Mashima, Nozaki and co-workers reported a related series of L^M5^Co­(II)_3_Ln­(III) (Ln­(III) = La­(III), Ce­(III), Pr­(III), Nd­(III), Eu­(III) or Gd­(III)) catalysts for CHO/CO_2_ ROCOP (Figure 16).^99^ In this series, the Co­(II)_3_Nd­(III) combination was the most active with a TOF = 1625 h^–1^ and 98% polymer selectivity (1:21250 [catalyst]_0_:[CHO]_0_, > 99% polycarbonate linkages, 130 °C, 20 bar CO_2_ pressure, neat). This is in contrast to the related series of Zn­(II)_3_Ln­(III) complexes, in which Ce­(III) was the optimal partner for Zn­(II).^38^ These results imply that, while each metal has a separate role in the mechanism, the metals must be considered together as part of the catalyst structural optimization.

**Figure 16:**
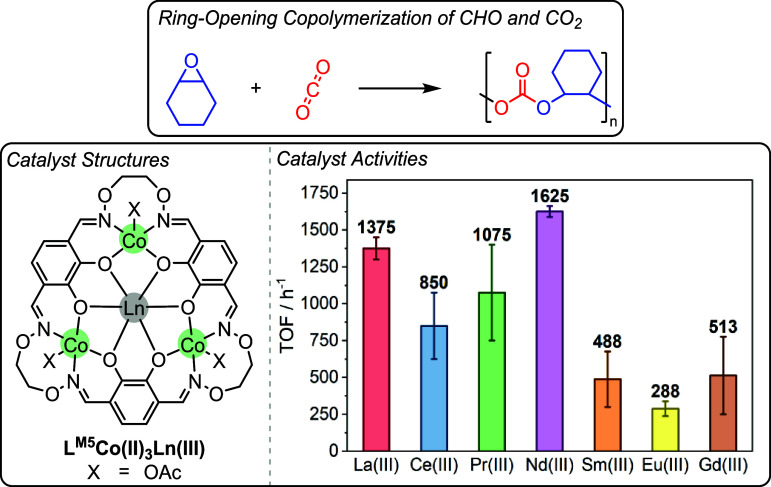
Tetranuclear L^M5^Co­(II)_3_Ln­(III) catalysts for the ring-opening copolymerization of cyclohexene oxide and CO_2_, as reported by Nozaki and co-workers.^99^ Adapted from ref. 99. Copyright 2020 American Chemical Society.

In 2023, Mashima, Okuda, and co-workers published a third report investigating this family of tetranuclear catalysts, using L^M7^ M­(II)_3_Ca­(II) (M­(II) = Co­(II), Mn­(II), Fe­(II), Ni­(II), Cu­(II) or Zn­(II)) catalysts for PO/CO_2_ ROCOP (Figure S21).^100^ Only the L^M7^Co­(II)_3_Ca­(II) catalyst was active, and the activity was significantly improved by adding a cocatalyst, specifically equimolar quantities of [PhMe_2_NH]­[B­(C_6_F_5_)_4_] (TOF = 102 h^–1^, 95% polymer selectivity, 98% polycarbonate linkages, 1:1:6000 [catalyst]_0_:[[PhMe_2_NH]­[B­(C_6_F_5_)_4_]]_0_:[PO]_0_, 50 °C, 10 bar CO_2_, neat). Experimental and DFT investigations support a mechanism where the ionic cocatalyst forms a cationic [Co­(III)]^n+^ intermediate (by acetate ligand abstraction). The cationic cobalt center is proposed to activate the PO, accelerating its ring-opening by the Ca­(II)-carbonate intermediate.

Overall, these recent studies signal that heterometallic catalysts can be highly effective when intermetallic synergy is feasible. Catalysts featuring transition metals with earth abundant s- and f-block metals show promise in terms of their low loadings, high rates and high selectivity for polycarbonate and polyester production (using different catalytic cycles).

The heterometallic catalysts are likely advantaged compared to homodinuclear analogues by substituting atleast one of the toxic and expensive transition metals with earth-abundant s-block metals. This strategy could be useful to reduce cost and improve future sustainability. The identification of experimental quantified structure–activity or structure-selectivity relationships is rare in oxygenated monomer ROP and ROCOP polymerization catalysis. The phenoxy-imine-ether heteronuclear catalysts show promise across different oxygenated monomer polymerizations both in terms of their absolute performances and the ability to deconvolute structure-performance trends. When combined with detailed kinetics, computational analyses and catalyst characterization investigations, these new structure–activity and structure-selectivity correlations improve understanding of the polymerization mechanism. These studies also demonstrate that correlations of activity and selectivity with structural parameters apply beyond electrocatalysis and to thermal catalysis, i.e. reactions where redox chemistry is not required for catalysis. Future efforts should continue to identify the generality of these structure-performance relationships to improve synergistic polymerization catalysis.

## Olefin Polymerization

In contrast to the previously discussed field of ring-opening epoxide/heteroallene copolymerization and cyclic ester polymerization catalysts, many of the most successful olefin polymerization catalysts are not multinuclear.^24,80^ There has, however, been a long-standing track record of investigations into synergistic dinuclear catalysts that apply ligands beyond the scope of those addressed in this perspective.^27,101,102^ In some circumstances, dinuclear catalysts have shown better performances than their mononuclear analogues, and there may be evidence of rate enhancements due to intermetallic synergy.^103–106^ Here, the focus is on Ni­(II)­M­(I) catalysts, using L^H^ ancillary ligands, for ethylene polymerization catalysis (Figure 17).^6,15,39,107^

**Figure 17:**
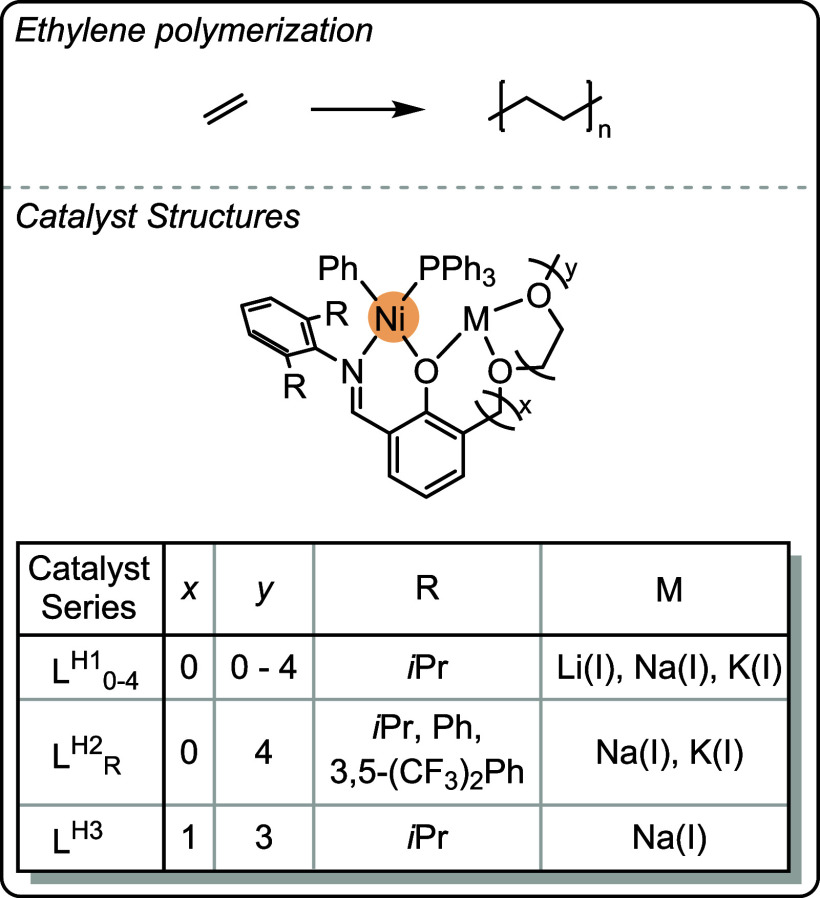
Top: Reaction scheme for the ethylene polymerization. Bottom: Series of ethylene polymerization L^H^Ni­(II)­M­(I) catalysts reported by Do and co-workers.^15,39,107^ Reproduced from refs. 15, 39, 107. Copyright 2017, 2024, 2015 American Chemical Society.

In 2015, Do and co-workers reported a series of Ni­(II) ethylene polymerization catalysts, coordinated by L^H1^_0–4_ ligands, with Group I metal BAr^F^ salts added *in situ*, which are proposed to coordinate to the ether donors to form the Ni­(II)­M­(I) heterodinuclear complexes (Figure 17).^107^ The *in situ*-generated Ni­(II)­M­(I) catalysts, when used alongside Ni­(COD)_2_ as a phosphine scavenger, all produced semicrystalline polyethylene with moderate activities of 2500–2800 g mol^–1^ h^–1^ ([catalyst]_0_:[Ni­(COD)_2_]_0_ = 1:2, [catalyst]_0_ = 4.8 mM, 6.9 bar, 5 mL toluene, 1 h, room temperature). UV–vis spectroscopy studies were used to calculate M­(I) association constants for M­(I) = Li­(I), Na­(I), K­(I) to the Ni­(II) complexes with ether chains of n = 1–4 (Figure S22).^107^

The authors proposed that the effect of M­(I) on the rate of polymerization was correlated to the number of ether donors and the nature of the s-block metal. The ligand with the longest ether chain showed a 20-fold increase in activity with the addition of Na­(I)­Bar^F^_4_, while the ligand with the shortest chain showed a decrease in activity with the addition of the metal salts. The catalyst with the highest activity, L^A1^_4_Ni­(II)­Na­(I), also has the highest association constant, while the catalysts with the lowest association constants also have the lowest activity (Figure S22). In accordance with the increase in activity with increasing binding strength, the authors propose that the Group I metal may increase the electrophilicity of the Ni­(II) center, accelerating ethylene binding and insertion.

A 2017 study by the same group found that the choice of Group I metal, between Na­(I) and K­(I), and differing aryl imine substituents, appeared to play a role in determining the microstructure and molecular weight of polyethene formed, alongside the rate of polymerization (Figure 17, L^H2^).^15^ Each of the dinuclear catalysts were more active than the monometallic Ni­(II) catalysts, with the Ni­(II)­Na­(I) catalysts outperforming the K­(I) analogues ([L^H2^Ni­(II)]_0_:[B­(C_6_F_5_)_3_]_0_:[M­(I)­BAr^F^_4_]_0_ = 1:1:1; [catalyst]_0_ = 1.5 mM, 6.9 bar ethylene, 10 mL toluene, 1 h, room temperature).

In 2024, Do and colleagues further improved the performance of the L^H^Ni­(II)­M­(I) catalysts by modifying the ligand through the addition of a methyl group between the aromatic ring and the ether oxygen (Figure 17, L^H3^).^39^ The authors argued that the more flexible ligand resulted in selective formation of only the monomeric, heterodinuclear catalyst, observed by X-ray crystallography. The isolated heterodinuclear complex showed higher activity compared to the previously reported catalysts. This was proposed to be due to decreasing the intermetallic separation between Ni­(II) and M­(I) centers, and enhancement of the electrophilicity of the Ni­(II) center.

The series of studies conducted on the L^H^Ni­(II)­M­(I) ethylene polymerization catalysts highlight the benefits of synergy in controlling catalytic activity and selectivity, enabling access to controlled polymer architectures with varying molecular weights and microstructure. In the case of the L^H^Ni­(II)­M­(I) catalysts, intermetallic synergy was proposed to originate from the s-block metal, M­(I), exerting control over the electrophilicity of the Ni­(III) center. While the hypothesis has not yet been quantified or explored using other olefins, the structure–activity data agrees with linear dependences of transition metal redox potentials with the Lewis acidity of neighboring M­(I), which have been repeatedly observed in electrocatalysis.^11,12^ This potential correlation exemplifies the benefits of cross-referencing studies from other fields of catalysis to inform upon origins/bases for intermetallic synergy.

## Asymmetric Catalysis

Following the observation of the electrocatalysts' and polymerization catalysts’ synergy and structure-performance trends, we were interested to understand how related families of heteronuclear catalysts, coordinated by the same ligands, were used in other fields such as asymmetric organic transformations. It is worth emphasizing that in some of the following reports, the primary subjects under investigation were not quantitative or mechanistic investigations of intermetallic synergy but rather the authors were deliberately targeting mixed metal catalysts due to the performance benefits associated with heteronuclear synergy. We considered that with the knowledge gained from the quantified structure-performance relationships from the electrochemistry and polymerization catalysis fields, it may be appropriate to reconsider these previously reported catalysts to understand whether a similar rationale might retrospectively be applied to their performances.

In asymmetric organic catalysis, heteronuclear catalysts featuring the ligand structures considered in this perspective have been successfully utilized in a wide range of different chemical transformations. These studies are often highly pragmatic in their approach – the primary topic of discussion and investigation is often catalytic performance as assessed by activity data obtained from point kinetics, and control as assessed by enantioselectivity and/or diastereoselectivity, rather than detailed investigations of the mechanism via kinetic and/or computational analysis, with some exceptions. Further, in many reports the catalyst complexes were not isolated or characterized, and neither were any of the proposed intermediates. Instead, it is common practice to generate the heteronuclear catalysts *in situ* by combining the proligand and two metal salts, or by combining a monometallic complex with an additional metal salt during the reaction.

Nonetheless, much of the requisite data enabling some insights into intermetallic synergy in these catalysts have been included in the original reports, allowing us to explore the generality of structure-performance trends. In each case, we fitted the data using linear functions to avoid overinterpretation and/or overfitting of the single time-point kinetic data.

A 2001 report from Kozlowski and colleagues identified the potential for these heteronuclear catalysts for the asymmetric Michael addition of benzyl malonate to cyclohexanone (Figure 18).^16^ The ligand, L^A6^, featured two 1,1′-bi-2-naphthol moieties tethered to a salen binding pocket, which enabled coordination of three metal centers in close proximity. The authors combined various monometallic transition metal complexes with Group I metal salts to generate the heteronuclear catalysts *in situ* (L^A4^ M­(II)­M­(I)_2_ where M­(II) = Ni­(II), Cu­(II), Pd­(II), Zn­(II) and M­(I) = Li­(I), Na­(I), K­(I), Cs­(I)). The best performing catalyst was the Ni­(II)­Cs­(I)_2_ combination, which formed the Michael addition product with a yield of 79%, and 71% enantiomeric excess after optimization of reaction conditions (1:1:5:5 [L^A6^H_2_Ni­(II)]_0_:[Cs_2_CO_3_]_0_:[cyclohexeneone]_0_:[benzyl malonate]_0_, room temperature, THF, 48 h). A later report isolated the L^A6^Ni­(II)­Cs­(I)_2_ catalyst and showed that this species exhibits the same performance as the *in situ* generated species under the same conditions.^108^ A cooperative dinuclear mechanism was proposed following kinetic analysis showing first-order reaction kinetics with respect to catalyst concentration. The roles of the metals were explored by testing the monometallic complexes: the monometallic Ni­(II) complex was inactive, while a monometallic Cs­(I) naphthalate complex showed turnover, but a lack of enantioselectivity.^108^ Further, the Pd­(II)­Cs­(I) analog showed slightly reduced activity (70% yield after 48 h) compared to the Ni­(II)­Cs­(I) combination (79% yield after 48 h), but a significantly lower enantioselectivity of 10%, compared to 71% for the Ni­(II)­Cs­(I). These data seem to indicate that the Ni­(II) site is responsible in part for achieving high enantioselectivity and the Cs­(I) site is predominantly responsible for achieving high activities.

**Figure 18:**
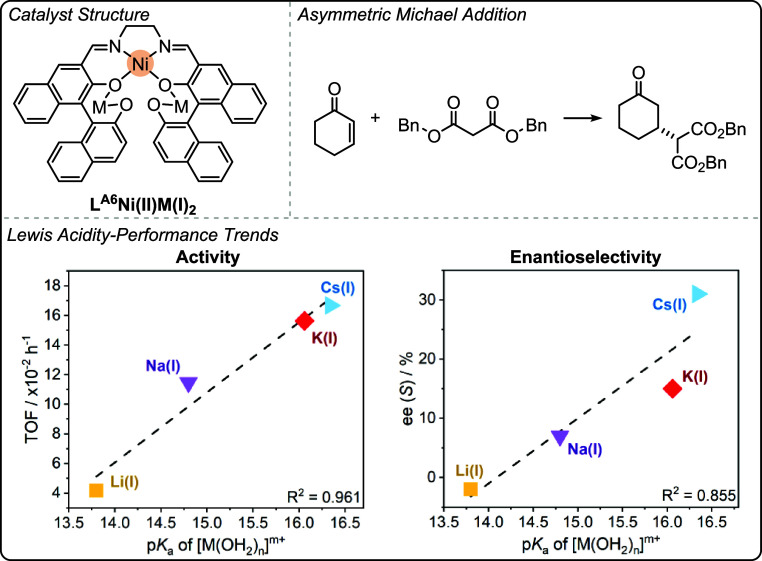
Top: Proposed structure of L^A6^Ni­(II)­M­(I)_2_ catalysts reported by Kozlowski and DiMauro for asymmetric Michael additions.^16^ The catalyst is formed *in situ* from the monometallic L^A6^Ni­(II) complex and the respective s-block metal salt. Bottom: Correlations between activity and enantioselectivity with changing Lewis acidity of the s-block metals, obtained by plotting data reported in the original article with s-block metal aqua complex p*K*_a_.^16^ Adapted from ref. 16. Copyright 2001 American Chemical Society.

The authors proposed a mechanism where the malonate is initially deprotonated by one of the naphthoxides, followed by coordination of the resultant nucleophilic enolate by one of the Cs­(I) centers (Figure S23). The role of the Ni­(II) center is proposed to be coordination and stabilization of the cyclohexenone, which acts as the electrophile.^108^

We plotted activity and enantiomeric excess of each of the L^A6^Ni­(II)­M­(I) catalysts against the s-block metal Lewis acidity (measured using the p*K*_a_ of the metal aqua complexes; M­(I) = Li­(I), Na­(I), K­(I), Cs­(I)).^16^ The plots show that both enantioselectivity and catalyst activity increase with decreasing Lewis acidity of the s-block metal aqua complexes (Figure 18). This may help to substantiate the authors’ proposal that the nucleophilic enolate is coordinated by one of the Cs­(I) centers; the weak Lewis acidity of the Cs­(I) center may provide a highly labile enolate, which can then rapidly react with the Ni­(II)-bound cyclohexenone. Correlations with changing transition metal Lewis acidity or ionic radius were less clear; there may be a correlation between enantioexcess and yield with Ni­(II), Cu­(II), and Pd­(II), with Zn­(II) an exception (Figure S24). However, more data points would be necessary to understand if there are truly correlations between transition metal Lewis acidity and/or ionic radius and L^A6^ M­(II)­Cs­(I) catalyst performance.

In 2007, Matsunaga, Shibasaki, and co-workers reported a series of heterodinuclear catalysts, formed *in situ* by combining the H_2_L^A7^ proligand with transition metal acetate salts and lanthanide isopropoxide salts (L^A7^ M­(II)­M­(III) where M­(II) = Cu­(II), Zn­(II), Mg­(II), Ni­(II), Rh­(II) and M­(III) = La­(III), Pr­(III), Sm­(III), Eu­(III), Dy­(III)).^17^ These catalyst mixtures were tested for the *syn*-selective asymmetric nitro-Mannich (or aza-Henry) reaction for the synthesis of β-nitroamines (Figure 19).^17^ The best catalyst of the series was the Cu­(II)­Sm­(III) combination, which allowed the desired *syn*-product (*R, R*) to be obtained in 96% yield, with >95% diastereoselectivity and 94% enantioexcess after optimization of reaction conditions (1:1:1:1:5:5 [H_2_L^A7^]_0_:[Cu­(OAc)_2_]_0_:[Sm­(O^i^Pr)_3_]_0_:[4-^t^Bu-phenol]_0_:[*N*-Boc imine]_0_:[nitroethane]_0_; −40 °C, THF, 23 h).^17^

**Figure 19:**
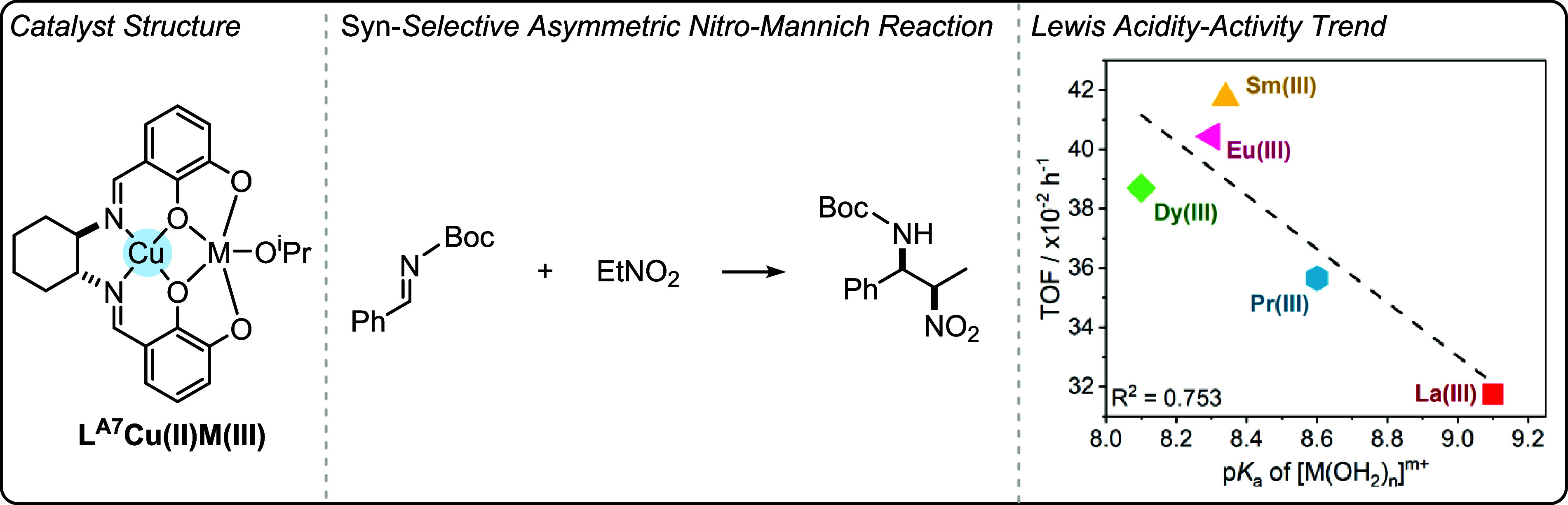
L^A7^Cu­(II)­M­(III) catalysts, reported by Shibasaki and co-workers, for syn-selective asymmetric nitro-Mannich reaction (LHS and middle).^17^ Correlation between activity and M­(III) Lewis acidity, obtained by plotting data reported in the original article with lanthanide aqua complex p*K*_a_s (RHS).^17^ Adapted from ref. 17. Copyright 2007 American Chemical Society.

For the Cu­(II)­M­(III) series, we observe a decrease in catalyst activity against M­(III) Lewis acidity (measured by aqua complex p*K*_a_). Analogous correlations with enantioexcess and diastereoselectivity were weaker (Figures S25 and S26). No such correlations between activity and/or selectivity were observed with the changing divalent metals; L^A7^Cu­(II)­Sm­(III) showed the best activity, diastereoselectivity, and enantioselectivity. Based on initial rates, kinetic isotope, and nonlinear effect experiments, the authors proposed that the active catalyst is a μ-oxo-μ-aryloxo trimeric species, containing an additional phenoxy ligand which bridges the Sm­(III) centers. The addition of an achiral phenol at the beginning of the reaction is proposed to form this species by breaking up larger aggregates. The authors proposed that the rate-determining step involved deprotonation of the nitroalkane by the bridging phenoxy ligand (Figure S28).^109^

The Sm­(III) center may, therefore, need to be sufficiently Lewis acidic to bind the deprotonated nitroalkane. The imine substrate was, in turn, proposed to coordinate to the Cu­(II) center via the Boc protecting group, favoring a transition state where the *syn* diastereomer is formed preferentially via addition-protonation.

A similar catalyst, featuring the same L^A7^ ligand structure, was reported in 2008 for the *anti*-selective asymmetric nitro-aldol reaction (Figure 19–20).^18^ Again, four transition metals were combined with four lanthanides (M­(II) = Cu­(II), Ni­(II), Zn­(II), Pd­(II)) and M­(III) = La­(III), Sm­(III), Gd­(III), Dy­(III), respectively). In this case, the L^A7^Pd­(II)­La­(III) combination was the most active and selective after optimization of reaction conditions (92% yield, *anti*/*syn* = 19:1, 84% *ee*, 1:1:1:1:5:5 [H_2_L^BN^]_0_:[Pd­(OAc)_2_]_0_:[La­(O^*i*^Pr)_3_]_0_:[4- BrC_6_H_4_OH]_0_:[benzaldehyde]_0_:[EtNO_2_]_0_, - 40 °C, THF/xylenes, 69 h).^18^

**Figure 20:**
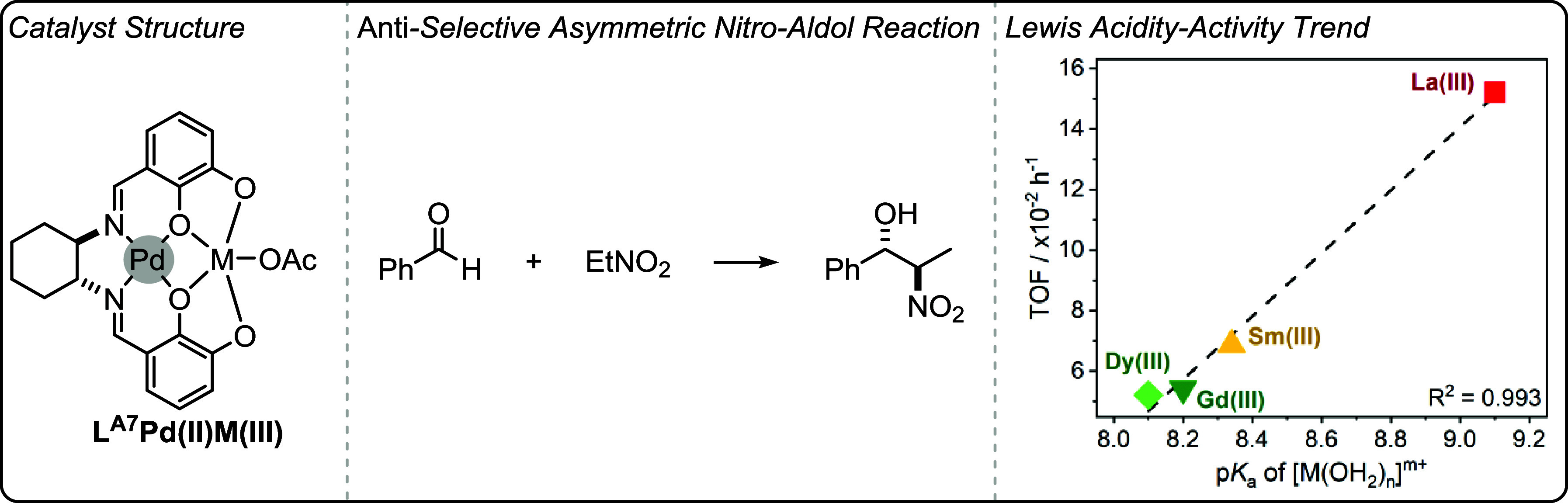
Structure of L^A7^Pd­(II)­M­(III) catalysts (LHS), reported by Shibasaki and co-workers, for antiselective asymmetric nitro-aldol coupling (middle).^18^ Correlation between activity and lanthanide aqua complex p*K*_a_s obtained by plotting data reported in the original article with (RHS).^18^ Adapted with permission from ref. 18. Copyright 2008 John Wiley and Sons.

Initially, the authors performed the lanthanide scope using L^A7^Cu­(II)­M­(III).^18^ When we reanalyzed this Cu­(II)­M­(III) series data from the original publication, we observe a linear increase in activity with decreasing lanthanide Lewis acidity for the L^A7^Cu­(II)­M­(III) catalysts (Figure 19). L^A7^Cu­(II)­La­(III) had the highest activity and enantioselectivity. It did, however, also have the lowest selectivity for the *anti*-diastereomer, although these selectivities varied comparatively little between the tested catalysts (Figure S29).

Subsequently, the authors investigated the performance of different transition metals in combination with La­(III) and identified L^A7^Pd­(II)­La­(III) as the most active catalyst. When were plotted the original data, we may observe correlations between performance and metal Lewis acidity as well as ionic radius of Pd­(II), Cu­(II), and Ni­(II); Zn­(II) is an outlier and does not follow these correlations (Figures S27, S29 and S30). While these correlations are similar to those for the previously discussed L^A6^ M­(II)­Cs­(I) catalysts reported by Kozlowski and co-workers, no definitive conclusions can be drawn due to the small number of data points.

In 2009, Matsunaga, Shibasaki, and co-workers reported a related series of catalysts for the α-addition of isocyanides to aldehydes.^19^ Catalysts were proposed to form *in situ* by combining the H_2_L^A8^ proligand with M­(III) isopropoxide and lanthanide triflate salts (M_1_(III) = Al­(III), Ga­(III), In­(III), Yb­(III), and M_2_(III) = Yb­(III), Gd­(III), Nd­(III), La­(III), respectively; Figure 21).^19^ The most active catalyst was the Ga­(III)­Yb­(III) combination, which achieved complete turnover with 96% enantioexcess over 24 h (1:1:0.95:5:5 [H_2_L^A6^]_0_: [Ga­(O^*i*^Pr)_3_]_0_: [Yb­(OTf)_3_]_0_: [isocyanide]_0_: [benzaldehyde]_0_, - 20 °C, CHCl_3_).

**Figure 21:**
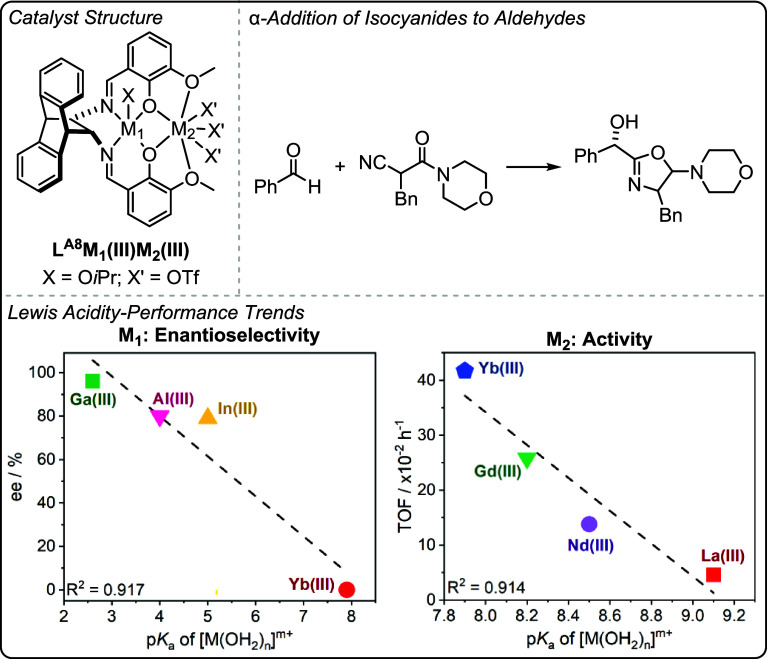
Top: Structure of L^A8^ M_1_(III)­M_2_(III) catalysts, reported by Shibasaki and co-workers, for the α-addition of isocyanides to aldehydes.^19^ Bottom: Correlations between enantioselectivity and catalyst activity with changing lanthanide Lewis acidity, obtained by plotting data reported in the original article with lanthanide aqua complex p*K*_a_s.^19^ Adapted from ref. 19. Copyright 2009 American Chemical Society.

When we plotted the enantioselectivities of the L^A8^M_1_(III)­Yb­(III) against M_1_(III) Lewis acidity, as measured by aqua complex p*K*_a_, a clear decrease in enantioexcess with decreasing metal Lewis acidity was observed (Figure 21). Plotting the activities of the L^A8^Ga­(III)­M_2_(III) catalysts against the Lewis acidity of the lanthanide metals again showed a decrease in turnover frequency with decreasing Lewis acidity. Plots of changing L^A8^ M_1_(III)­Yb­(III) enantioselectivity and L^A8^Ga­(III)­M_2_(III) activity did not show strong correlations (Figure S31). These correlations appear to indicate that, for both metal centers, the best metals are also the strongest Lewis acids. However, it should be noted that the metals tested for the M_1_(III) scope are much more strongly Lewis acidic than those tested for the M_2_(III) scope. Indeed, the weakest Lewis acid used in the M_1_(III) scope, and the strongest Lewis acid used in the M_2_(III) scope are both Yb­(III)).

The authors proposed a mechanism where one metal coordinates and activates the aldehyde, and the other metal coordinates the isocyanide within sufficient proximity to allow for its nucleophilic attack at the aldehyde.^19^ Considering the relative Lewis acidities of the two metals in the optimized catalyst, it might be expected that the strongly acidic Ga­(III) center may be responsible for activation of the aldehyde, in the correct configuration, which is proposed to act as the electrophile in the reaction. The weaker Lewis acid Yb­(III) center may be expected to coordinate the nucleophilic isocyanide. Decreasing the Lewis acidity of the lanthanide center may result in decreased activity as the isocyanide may be too weakly coordinated to be held sufficiently close to the aldehyde to allow efficient nucleophilic attack.

In 2011 and 2014, the same group reported a series of L^A9^ M_1_(III)­M_2_(III) catalysts, prepared *in situ* by using mixtures of lanthanide or Group III *iso*-propoxide and triflate salts, for the asymmetric ring-opening of *meso* aziridines with malonates, and the regiodivergent kinetic resolution of racemic aziridines with malonates, respectively (aziridine ring-opening: M_1_(III) = La­(III), Nd­(III), Sm­(III), Gd­(III), Er­(III), Sc­(III), M_2_(III) = Yb­(III), Y­(III), Gd­(III), Sm­(III), La­(III); kinetic resolution of aziridines: M_1_(III) = La­(III), Nd­(III), Sm­(III), Gd­(III), Er­(III), M_2_(III) = Yb­(III), Y­(III), Gd­(III), Sm­(III), La­(III); Figure 22).^20,110^ For both transformations, we plotted catalyst activity against the Lewis acidity of M_1_(III), measured by aqua complex p*K*_a_, and observed that there is an increase in activity with decreasing metal Lewis acidity for both transformations, with La­(III) giving the highest activity (Figure 22). For the regiodivergent kinetic resolution of racemic aziridines, catalysts L^A9^ M_1_(III)­Yb­(III) with Er­(III), Sm­(III), and Gd­(III) were all inactive under the tested conditions, perhaps due to their high Lewis acidities.

**Figure 22:**
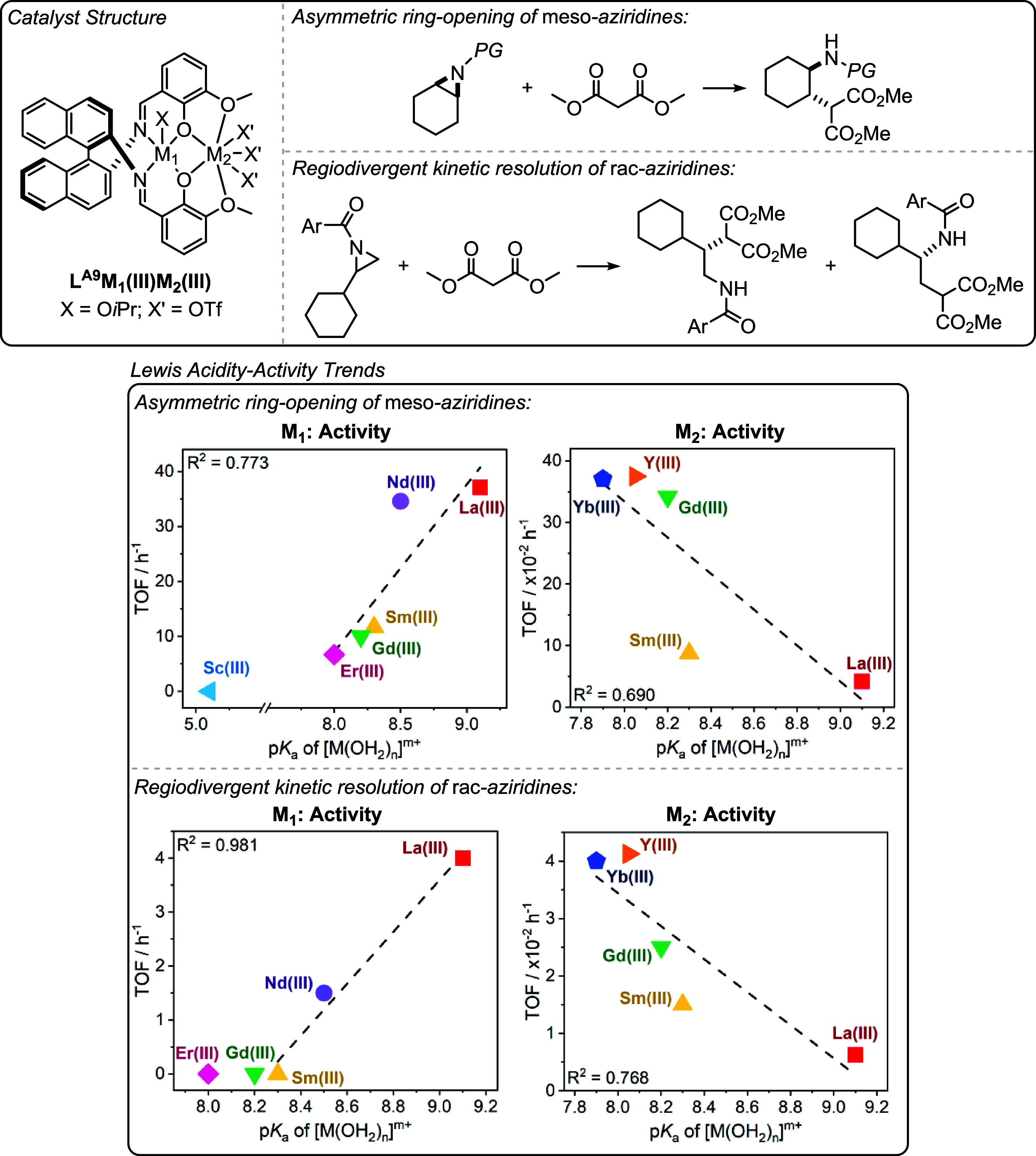
Top: Structure of L^A9^ M_1_(III)­M_2_(III) catalysts for the asymmetric ring-opening of meso-aziridines, and the regiodivergent kinetic resolution of racemic aziridines, as reported by Shibasaki and co-workers.^20,110^ Bottom: Correlations between catalyst activity and changing lanthanide Lewis acidity, obtained by plotting data reported in the original article with lanthanide aqua complex p*K*_a_.^20,110^ Adapted from refs. 20, 110. Copyright 2011 and 2014 American Chemical Society.

When M_2_(III) was varied systematically in catalyst L^A9^La­(III)­M_2_(III) the opposite result was observed for both reactions; the best activities were achieved with the La­(III)­Yb­(III) and La­(III)­Y­(III) metal combinations, with a decrease in activity with decreasing metal Lewis acidity (i.e., increasing p*K*_a_ of the aqua complex; M_2_(III) = Yb­(III), Y­(III), Gd­(III), Sm­(III), La­(III))).

These correlations corroborate the argument proposed by the authors, where for both reactions the La­(III) center is proposed to deprotonate the malonate, and the Yb­(III) or Y­(III) center is proposed to act as a Lewis acid.^20,110^ While the mechanisms of the transformations were not further investigated, it might be expected that the La­(III) center coordinates the resultant nucleophilic species, while the Yb­(III) or Y­(III) center may perhaps activate the aziridine to ring-opening. For both metal series, no real differences in enantioselectivity were observed, as all tested catalysts produced the desired product with >90% ee. This may imply that the predominant factor affecting enantioselectivity for this catalyst is the chiral backbone, rather than metal selection.

Considering the use of L^A^M­(II/III)­M­(I/III) complexes in organic small-molecule asymmetric catalysis further illustrates the versatility of these heteronuclear catalysts. The repeated finding of structure-performance correlations between catalyst activity and selectivities against s-block and lanthanide metal Lewis acidity (as measured by aqua complex p*K*_a_) for various organic transformations establishes both its generality and importance across three branches of catalysis: electrocatalysis, polymerization catalysis, and asymmetric catalysis.

The correlations between enantioselectivity and diastereoselectivity and varying s- or f-block metal Lewis acidity highlight the potential for metal synergy, as well as chiral ligands, in optimizing control. The lack of clear correlations between catalyst performance and changing transition metal Lewis acidity is both expected and rational since ligand field stabilization and orbital energies would be expected to play a more significant role than simply metal Lewis acidity. The lanthanides and s-block metals, on the other hand, are more appropriately considered as simple Lewis acids of various strengths, leading to the correlations we repeatedly observe.

## Conclusions

This perspective highlights the benefits of quantified structure-performance trends to investigate the underpinning bases for intermetallic synergy in a series of homogeneous catalysts used in three diverse fields of chemistry: electrochemistry, polymerization catalysis and asymmetric organic transformations. The catalysts examined in this perspective are homogeneous complexes featuring a transition metal or lanthanide, M­(II–III), partnered with an s-block, lanthanide or Group III metal, M­(I–III). They are all coordinated by phenoxy-imine-ether donor ligands, classified here into three subfamilies. The ligands are generally straightforward to synthesize and derivatize, facilitating structure-performance investigations. So far, each of these fields has developed their catalysts, understandings and instincts relating to how to exploit intermetallic synergy largely independently of each other. This perspective shows the potential of inter-field knowledge sharing and the common catalyst-structure performance relationships. Indeed, these fields each show the same generally applicable and quantifiable relationships between catalytic performance, i.e. activity and selectivity, with Group III, s- and f-block metal Lewis acidity (as measured through metal aqua complex p*K*_a_). Researchers working in electrochemistry were the first to establish these trends between the transition metal redox potential and/or rate of electron transfer and the Lewis acidity of an s-block, lanthanide or Group III metal. They have pioneered in characterizing synergistic complexes and catalysts, and proposing how synergy may operate. Recent studies of synergistic catalysts for epoxide/carbon dioxide or epoxide/cyclic anhydride ring-opening copolymerizations, and cyclic ester ring-opening polymerizations have all shown the same types of quantifiable correlations between both catalytic activity and selectivity, with s-block metal Lewis acidity.

To further probe the generality of the observed correlations, in this perspective, we have re-analyzed previously reported data from the field of homogeneous catalysis for asymmetric organic transformations. Our analysis reveals the same trends relating catalyst activity, enantioselectivity, and occasionally diastereoselectivity, with s-block, lanthanide or Group III metal Lewis acidity in this third branch of catalysis.

This perspective suggests, albeit at this stage tentatively, that the “direction” of the correlations between catalytic activity and s-block metal Lewis acidity may provide a clue to the role of the studied metal in catalysis. Positive slopes with increasing metal aqua complex p*K*_a_ (i.e., decreasing Lewis acidity) seem to suggest that the s-block, lanthanide or Group III metal may bind the nucleophilic species in the rate-determining step, while negative slopes may imply that the s-block, lanthanide or Group III block metal is responsible for binding and activating the electrophile. A clear benefit of using quantified structure-performance trends to examine synergistic heteronuclear catalysts is, therefore, that it can help delineate the different functions or roles for the metals within the catalytic cycle. Kinetic experiments alone cannot define such roles, although researchers' hypotheses have often turned out to be remarkably accurate. Researchers often use computational methods, such as DFT, to study the roles of the different metals in catalysis, but an experimental method would be valuable to support such studies.

We emphasize the importance of using these structure-performance correlations together with, but not as a substitute for, full kinetic analyses, which are essential to draw any meaningful conclusions about the catalytic mechanism. Knowledge of the rate-determining step, and the key intermediate chemistry, is vital to appropriately interpret any structure-performance relationships. Researchers may benefit from using these structure-performance correlations, together with traditional kinetic studies supported by computational analyses, in determining the roles of the different metals in synergistic catalysts.

Considering future improvements to the use of these structure–activity studies to gain mechanistic insights, it is also important to further consider both how to optimize measurement or estimation of metal Lewis acidity and other experimental parameters which could inform upon it. Using the metal aqua complex p*K*_a_ as a proxy for Lewis acidity may be flawed in some circumstances and cannot account for influences of the ligand environment in producing subtle changes to Lewis acidity. Use of titration experiments to estimate relative Lewis acidity may not be appropriate for some catalysts, particularly those featuring sterically constrained environments, and may suffer from the lack of sensitivity of NMR spectroscopy. Attractive alternative measurements include those from recently reported electrochemical studies which apply spectroscopy to estimate the HOMO–LUMO gap and/or descriptors of frontier molecular orbital energies obtained by DFT calculations to measure relative s-block metal Lewis acidity. In any case, the structure-performance correlations should also be accompanied by catalyst characterization methods, preferably conducted under similar conditions to the true catalytic process (e.g., phase, concentration, additives, temperature).

Our objective in reviewing the literature is to highlight the potential for intermetallic synergy across different branches of catalysis and to help move the field beyond empirical observations and toward a rational design strategy. Clearly there is a lot more experimental and theoretical research remaining to be done prior to any complete understanding of intermetallic synergy, but the generality of these structure–activity relationships across the three branches of catalysis is significant. Both the insights from this work and the methods used to analyze and characterize the structure-performance relationships may be relevant to other catalysts, to fields where heterodinuclear complexes are known to out-perform their monometallic analogues such as in small molecule activation and bioinspired catalysis.^111–113^ Importantly, this perspective highlights that the fundamentals of intermetallic synergy seem to be transferable across fields. We hope the findings and methods presented in this perspective are broadly used to discover structure–activity trends which can aid in the rational development of novel, high-performance catalysts, and help to better understand and optimize for the roles of the different metals in the catalytic mechanism.

## Supplementary Material



## Notes

Overview of catalyst structures (Figures S1–S3), reaction schemes and catalytic cycles (Figures S8, S9, S14, S21, S23, and S28), further quantified structure-performance correlations (Figures S4, S6, S7, S10, S13, S15, S26, S27, S29, S31), qualitative structure-performance correlations illustrated using bar charts (Figures S5 and S16–S21) and examples of studies in which no structure-performance correlations were observed (Figures S11, S12, S24, S25, and S30) (PDF)

## †

F.F. is currently based at the David H Koch Institute for Integrative Cancer Research, Massachusetts Institute of Technology, Cambridge, MA 02139, USA

## Abbreviations

RDS: rate-determining step
PO: propene oxide
PPC: poly­(propene carbonate)
CHO: cyclohexene oxide
PCHC: poly­(cyclohexene oxide)

## References

[1] Léonard N. G., Dhaoui R., Chantarojsiri T., Yang J. Y. (2021). Electric Fields
in Catalysis: From Enzymes to Molecular Catalysts. ACS Catal..

[2] Lionetti D., Suseno S., Shiau A. A., de Ruiter G., Agapie T. (2024). Redox Processes Involving Oxygen:
The Surprising Influence
of Redox-Inactive Lewis Acids. JACS Au.

[3] Gruszka W., Garden J. A. (2021). Advances in heterometallic ring-opening (co)­polymerisation
catalysis. Nat. Commun..

[4] Park J., Hong S. (2012). Cooperative bimetallic
catalysis in asymmetric transformations. Chem.
Soc. Rev..

[5] Yoo C., Dodge H. M., Miller A. J. M. (2019). Cation-controlled catalysis with
crown ether-containing transition metal complexes. Chem. Commun..

[6] Cai Z., Xiao D., Do L. H. (2019). Cooperative
Heterobimetallic Catalysts
in Coordination Insertion Polymerization. Comments
Inorg. Chem..

[7] Quilis C., Mota N., Millán E., Pawelec B., Navarro
Yerga R. M. (2024). Application of Intermetallic Compounds as Catalysts
for the Selective Hydrogenation of CO_2_ to Methanol. ChemCatChem..

[8] Wu R., Meng Q., Yan J., Zhang Z., Chen B., Liu H., Tai J., Zhang G., Zheng L., Zhang J. (2024). Intermetallic
synergy in platinum–cobalt electrocatalysts
for selective C-O bond cleavage. Nat. Catal..

[9] Diment W. T., Lindeboom W., Fiorentini F., Deacy A. C., Williams C. K. (2022). Synergic
Heterodinuclear Catalysts for the Ring-Opening Copolymerization (ROCOP)
of Epoxides, Carbon Dioxide, and Anhydrides. Acc. Chem. Res..

[10] Zhong D.-C., Gong Y.-N., Zhang C., Lu T.-B. (2023). Dinuclear metal
synergistic catalysis for energy conversion. Chem. Soc. Rev..

[11] Reath A. H., Ziller J. W., Tsay C., Ryan A. J., Yang J. Y. (2017). Redox Potential
and Electronic Structure Effects of Proximal Nonredox Active Cations
in Cobalt Schiff Base Complexes. Inorg. Chem..

[12] Kumar A., Lionetti D., Day V. W., Blakemore J. D. (2018). Trivalent
Lewis Acidic Cations Govern the Electronic Properties and Stability
of Heterobimetallic Complexes of Nickel. Chem.
- Eur. J..

[13] Golwankar R. R., Kumar A., Day V. W., Blakemore J. D. (2022). Revealing
the Influence of Diverse Secondary Metal Cations on Redox-Active Palladium
Complexes. Chem. - Eur. J..

[14] Karnes J. P., Kumar A., Hopkins
Leseberg J. A., Day V. W., Blakemore J. D. (2024). Trivalent
Cations Slow Electron Transfer to Macrocyclic Heterobimetallic Complexes. Inorg. Chem..

[15] Cai Z., Do L. H. (2017). Customizing Polyolefin
Morphology by Selective Pairing of Alkali
Ions with Nickel Phenoxyimine-Polyethylene Glycol Catalysts. Organometallics.

[16] DiMauro E. F., Kozlowski M. C. (2001). BINOL–Salen
Metal Catalysts Incorporating a
Bifunctional Design. Org. Lett..

[17] Handa S., Gnanadesikan V., Matsunaga S., Shibasaki M. (2007). syn-Selective
Catalytic Asymmetric Nitro-Mannich Reactions Using a Heterobimetallic
Cu–Sm–Schiff Base Complex. J.
Am. Chem. Soc..

[18] Handa S., Nagawa K., Sohtome Y., Matsunaga S., Shibasaki M. (2008). A Heterobimetallic Pd/La/Schiff Base
Complex for anti-Selective
Catalytic Asymmetric Nitroaldol Reactions and Applications to Short
Syntheses of β-Adrenoceptor Agonists. Angew. Chem., Int. Ed..

[19] Mihara H., Xu Y., Shepherd N. E., Matsunaga S., Shibasaki M. (2009). A Heterobimetallic
Ga/Yb-Schiff Base Complex for Catalytic Asymmetric α-Addition
of Isocyanides to Aldehydes. J. Am. Chem. Soc..

[20] Xu Y., Lin L., Kanai M., Matsunaga S., Shibasaki M. (2011). Catalytic
Asymmetric Ring-Opening of meso-Aziridines with Malonates under Heterodinuclear
Rare Earth Metal Schiff Base Catalysis. J. Am.
Chem. Soc..

[21] Matsunaga S., Shibasaki M. (2014). Recent advances in cooperative bimetallic asymmetric
catalysis: dinuclear Schiff base complexes. Chem. Commun..

[22] Chantarojsiri T., Reath A. H., Yang J. Y. (2018). Cationic
Charges Leading to an Inverse
Free-Energy Relationship for N–N Bond Formation by MnVI Nitrides. Angew. Chem., Int. Ed..

[23] Chantarojsiri T., Ziller J. W., Yang J. Y. (2018). Incorporation
of redox-inactive cations
promotes iron catalyzed aerobic C-H oxidation at mild potentials. Chem. Sci..

[24] Fiorentini F., Diment W. T., Deacy A. C., Kerr R. W. F., Faulkner S., Williams C. K. (2023). Understanding catalytic
synergy in dinuclear polymerization
catalysts for sustainable polymers. Nat. Commun..

[25] Lindeboom W., Deacy A. C., Phanopoulos A., Buchard A., Williams C. K. (2023). Correlating
Metal Redox Potentials to Co­(III)­K­(I) Catalyst Performances in Carbon
Dioxide and Propene Oxide Ring Opening Copolymerization. Angew. Chem., Int. Ed..

[26] Park D., Jette C. I., Kim J., Jung W.-O., Lee Y., Park J., Kang S., Han M. S., Stoltz B. M., Hong S. (2020). Enantioselective Alkynylation
of Trifluoromethyl Ketones Catalyzed
by Cation-Binding Salen Nickel Complexes. Angew.
Chem., Int. Ed..

[27] Delferro M., Marks T. J. (2011). Multinuclear Olefin Polymerization Catalysts. Chem. Rev..

[28] Jacobsen E. N. (2000). Asymmetric
Catalysis of Epoxide Ring-Opening Reactions. Acc. Chem. Res..

[29] Clarke R. M., Storr T. (2014). The chemistry and applications of multimetallic salen complexes. Dalton Trans..

[30] Andruh M. (2015). The exceptionally
rich coordination chemistry generated by Schiff-base ligands derived
from o-vanillin. Dalton Trans..

[31] Juyal V. K., Pathak A., Panwar M., Thakuri S. C., Prakash O., Agrwal A., Nand V. (2023). Schiff base
metal complexes as a
versatile catalyst: A review. J. Organomet.
Chem..

[32] Haak R. M., Wezenberg S. J., Kleij A. W. (2010). Cooperative multimetallic catalysis
using metallosalens. Chem. Commun..

[33] Shaw S., White J. D. (2019). Asymmetric Catalysis
Using Chiral Salen-Metal Complexes:
Recent Advances. Chem. Rev..

[34] Van
Staveren C. J., Van Eerden J., Van Veggel F. C. J. M., Harkema S., Reinhoudt D. N. (1988). Cocomplexation of neutral guests
and electrophilic metal cations in synthetic macrocyclic hosts. J. Am. Chem. Soc..

[35] Van
Veggel F. C. J. M., Harkema S., Bos M., Verboom W., Van Staveren C. J., Gerritsma G. J., Reinhoudt D. N. (1989). Metallomacrocycles:
synthesis, x-ray structure, electrochemistry, and ESR spectroscopy
of mononuclear and heterodinuclear complexes. Inorg. Chem..

[36] Kang K., Fuller J., Reath A. H., Ziller J. W., Alexandrova A. N., Yang J. Y. (2019). Installation of internal electric fields by non-redox
active cations in transition metal complexes. Chem. Sci..

[37] Dopp C. M., Golwankar R. R., Kelsey S. R., Douglas J. T., Erickson A. N., Oliver A. G., Day C. S., Day V. W., Blakemore J. D. (2023). Vanadyl
as a Spectroscopic Probe of Tunable Ligand Donor Strength in Bimetallic
Complexes. Inorg. Chem..

[38] Nagae H., Aoki R., Akutagawa S.-N., Kleemann J., Tagawa R., Schindler T., Choi G., Spaniol T. P., Tsurugi H., Okuda J. (2018). Lanthanide Complexes Supported by a Trizinc Crown Ether
as Catalysts for Alternating Copolymerization of Epoxide and CO2:
Telomerization Controlled by Carboxylate Anions. Angew. Chem., Int. Ed..

[39] Ruiz
De Castilla L. C., Ganguly T., Tahmouresilerd B., Laconsay C. J., Wu J. I., Do L. H. (2024). Optimizing the Cation
Binding Pocket in Nickel Phenoxyimine Catalysts Improves Ethylene
Polymerization Efficiency. Organometallics.

[40] Richard J. P., Cristobal J. R., Amyes T. L. (2021). Linear Free Energy Relationships
for Enzymatic Reactions: Fresh Insight from a Venerable Probe. Acc. Chem. Res..

[41] Williams, A. ; Williams, J. Free Energy Relationships in Organic and Bio-organic Chemistry; The Royal Society of Chemistry: 2003. 10.1039/978184755

[42] Léonard N. G., Chantarojsiri T., Ziller J. W., Yang J. Y. (2022). Cationic Effects
on the Net Hydrogen Atom Bond Dissociation Free Energy of High-Valent
Manganese Imido Complexes. J. Am. Chem. Soc..

[43] Teindl K., Patrick B. O., Nichols E. M. (2023). Linear Free Energy Relationships
and Transition State Analysis of CO_2_ Reduction Catalysts
Bearing Second Coordination Spheres with Tunable Acidity. J. Am. Chem. Soc..

[44] Tsui E. Y., Agapie T. (2013). Reduction potentials
of heterometallic manganese–oxido
cubane complexes modulated by redox-inactive metals. Proc. Natl. Acad. Sci. U. S. A..

[45] Perrin, D. D. Ionisation Constants of Inorganic Acids and Bases in Aqueous Solution; Elsevier: 1982. 10.1016/B978-0-08-029214-4.500

[46] First Transition Series Metals. In Hydrolysis of Metal Ions, Brown, P. L. ; Ekberg, C. , Eds.; John Wiley & Sons: 2016; pp 499–716.

[47] Kumar A., Blakemore J. D. (2021). On the Use of Aqueous Metal-Aqua
pKa Values as a Descriptor
of Lewis Acidity. Inorg. Chem..

[48] Kobayashi S., Nagayama S., Busujima T. (1998). Lewis Acid Catalysts Stable in Water.
Correlation between Catalytic Activity in Water and Hydrolysis Constants
and Exchange Rate Constants for Substitution of Inner-Sphere Water
Ligands. J. Am. Chem. Soc..

[49] Kumar A., Lionetti D., Day V. W., Blakemore J. D. (2020). Redox-Inactive
Metal Cations Modulate the Reduction Potential of the Uranyl Ion in
Macrocyclic Complexes. J. Am. Chem. Soc..

[50] Kelsey S. R., Kumar A., Oliver A. G., Day V. W., Blakemore J. D. (2021). Promotion
and Tuning of the Electrochemical Reduction of Hetero- and Homobimetallic
Zinc Complexes. ChemElectroChem..

[51] Karnes J. P., Lind N. M., Oliver A. G., Day C. S., Day V. W., Blakemore J. D. (2025). Tunability in Heterobimetallic Complexes Featuring
an Acyclic “Tiara” Polyether Motif. Inorg. Chem..

[52] Nguyen H. M., Morgan H. W. T., Chantarojsiri T., Kerr T. A., Yang J. Y., Alexandrova A. N., Léonard N. G. (2023). Charge and Solvent Effects on the
Redox Behavior of Vanadyl Salen-Crown Complexes. J. Phys. Chem. A.

[53] Maity S., Ghosh S., Ghosh A. (2019). Elucidating
the secondary effect
in the Lewis acid mediated anodic shift of electrochemical oxidation
of a Cu­(II) complex with a N_2_O_2_ donor unsymmetrical
ligand. Dalton Trans..

[54] Bhunia P., Gomila R. M., Frontera A., Ghosh A. (2023). Combined effects of
the lewis acidity and electric field of proximal redox innocent metal
ions on the redox potential of vanadyl Schiff base complexes: an experimental
and theoretical study. Dalton Trans..

[55] Bhunia P., Gomila R. M., Frontera A., Ghosh A. (2024). Shift of the reduction
potential of nickel­(II) Schiff base complexes in the presence of redox
innocent metal ions. Dalton Trans..

[56] Kumar A., Golwankar R. R., Pyrch M. M. F., Cooper F. L., Arehart G. A., Carter K. P., Oliver A. G., Day V. W., Forbes T. Z., Blakemore J. D. (2025). Macrocyclic
control of electron transfer to high valent
uranium in heterobimetallic complexes. Dalton
Trans..

[57] van
Veggel F. C. J. M., Verboom W., Reinhoudt D. N., Bos M. (1990). Electrochemical reduction of benzyl chloride catalyzed by a hetero-dinuclear
complex. Recl. Trav. Chim. Pays-Bas.

[58] Saveant, J.-M. ; Costentin, C. Elements of Molecular and Biomolecular Electrochemistry; John Wiley & Sons Inc.: 2019. DOI:10.1002/9781119292364.

[59] Marcus R. A. (1956). On the
Theory of Oxidation-Reduction Reactions Involving Electron Transfer. I. J. Chem. Phys..

[60] Shao H., Muduli S. K., Tran P. D., Soo H. S. (2016). Enhancing electrocatalytic
hydrogen evolution by nickel salicylaldimine complexes with alkali
metal cations in aqueous media. Chem. Commun..

[61] Böttcher A., Birnbaum E. R., Day M. W., Gray H. B., Grinstaff M. W., Labinger J. A. (1997). How do electronegative substituents make metal complexes
better catalysts for the oxidation of hydrocarbons by dioxygen?. J. Mol. Catal. A. Chem..

[62] Grinstaff M. W., Hill M. G., Labinger J. A., Gray H. B. (1994). Mechanism of Catalytic
Oxygenation of Alkanes by Halogenated Iron Porphyrins. Science.

[63] Labinger J. A. (1994). A simplified
model for catalyzed isobutane autoxidation: implications for the mechanism
of catalysis by halogenated porphyrin complexes. Catal. Lett..

[64] Larson V. A., Battistella B., Ray K., Lehnert N., Nam W. (2020). Iron and manganese
oxo complexes, oxo wall and beyond. Nat. Rev.
Chem..

[65] Hohenberger J., Ray K., Meyer K. (2012). The biology and chemistry
of high-valent iron–oxo
and iron–nitrido complexes. Nat. Commun..

[66] Bhat G. A., Darensbourg D. J. (2023). Coordination
complexes as catalysts for the coupling
reactions of oxiranes and carbon dioxide. Coord.
Chem. Rev..

[67] Yang G.-W., Xie R., Zhang Y.-Y., Xu C.-K., Wu G.-P. (2024). Evolution of Copolymers
of Epoxides and CO2: Catalysts, Monomers, Architectures, and Applications. Chem. Rev..

[68] Lidston C. A. L., Severson S. M., Abel B. A., Coates G. W. (2022). Multifunctional
Catalysts for Ring-Opening Copolymerizations. ACS Catal..

[69] Hillmyer M. A., Tolman W. B. (2014). Aliphatic Polyester Block Polymers:
Renewable, Degradable,
and Sustainable. Acc. Chem. Res..

[70] Zhang X., Fevre M., Jones G. O., Waymouth R. M. (2018). Catalysis as an
Enabling Science for Sustainable Polymers. Chem.
Rev..

[71] Yang G.-W., Wu G.-P. (2019). High-Efficiency Construction of CO_2_-Based Healable Thermoplastic
Elastomers via a Tandem Synthetic Strategy. ACS Sustainable Chem. Eng..

[72] Langanke J., Wolf A., Hofmann J., Böhm K., Subhani M. A., Müller T. E., Leitner W., Gürtler C. (2014). Carbon dioxide
(CO2) as sustainable feedstock for polyurethane production. Green Chem..

[73] Lee S. H., Cyriac A., Jeon J. Y., Lee B. Y. (2012). Preparation of thermoplastic
polyurethanes using in situ generated poly­(propylene carbonate)-diols. Polym. Chem..

[74] Rabnawaz M., Wyman I., Auras R., Cheng S. (2017). A roadmap towards green
packaging: the current status and future outlook for polyesters in
the packaging industry. Green Chem..

[75] Decode the Future of Proylene Oxide. https://www.chemanalyst.com/industry-report/propylene-oxide-po-market-755 (accessed 08/01/2025).

[76] Alagi P., Ghorpade R., Choi Y. J., Patil U., Kim I., Baik J. H., Hong S. C. (2017). Carbon
Dioxide-Based Polyols as Sustainable
Feedstock of Thermoplastic Polyurethane for Corrosion-Resistant Metal
Coating. ACS Sustainable Chem. Eng..

[77] Wang J., Zhang H., Miao Y., Qiao L., Wang X., Wang F. (2016). Waterborne polyurethanes
from CO_2_ based polyols with comprehensive
hydrolysis/oxidation resistance. Green Chem..

[78] Garden J.
A., Saini P. K., Williams C. K. (2015). Greater than the Sum of Its Parts:
A Heterodinuclear Polymerization Catalyst. J.
Am. Chem. Soc..

[79] Reis N. V., Deacy A. C., Rosetto G., Durr C. B., Williams C. K. (2022). Heterodinuclear
Mg­(II)­M­(II) (M = Cr, Mn, Fe Co, Ni, Cu and Zn) Complexes for the Ring
Opening Copolymerization of Carbon Dioxide/Epoxide and Anhydride/Epoxide. Chem. - Eur. J..

[80] Deacy A. C., Kilpatrick A. F. R., Regoutz A., Williams C. K. (2020). Understanding metal
synergy in heterodinuclear catalysts for the copolymerization of CO_2_ and epoxides. Nat. Chem..

[81] Gaston A. J., Greindl Z., Morrison C. A., Garden J. A. (2021). Cooperative Heterometallic
Catalysts for Lactide Ring-Opening Polymerization: Combining Aluminum
with Divalent Metals. Inorg. Chem..

[82] Yolsal U., Shaw P. J., Lowy P. A., Chambenahalli R., Garden J. A. (2024). Exploiting Multimetallic Cooperativity in the Ring-Opening
Polymerization of Cyclic Esters and Ethers. ACS Catal..

[83] Aida T., Maekawa Y., Asano S., Inoue S. (1988). Immortal polymerization:
polymerization of epoxide and.beta.-lactone with aluminum porphyrin
in the presence of protic compound. Macromolecules.

[84] Inoue S. (2000). Immortal polymerization:
The outset, development, and application. J.
Polym. Sci., Part A: Polym. Chem..

[85] Deacy A. C., Phanopoulos A., Lindeboom W., Buchard A., Williams C. K. (2022). Insights
into the Mechanism of Carbon Dioxide and Propylene Oxide Ring-Opening
Copolymerization Using a Co­(III)/K­(I) Heterodinuclear Catalyst. J. Am. Chem. Soc..

[86] Deacy A. C., Moreby E., Phanopoulos A., Williams C. K. (2020). Co­(III)/Alkali-Metal­(I)
Heterodinuclear Catalysts for the Ring-Opening Copolymerization of
CO2 and Propylene Oxide. J. Am. Chem. Soc..

[87] Eisenhardt K. H. S., Fiorentini F., Lindeboom W., Williams C. K. (2024). Quantifying CO2
Insertion Equilibria for Low-Pressure Propene Oxide and Carbon Dioxide
Ring Opening Copolymerization Catalysts. J.
Am. Chem. Soc..

[88] Shannon R. D. (1976). Revised
effective ionic radii and systematic studies of interatomic distances
in halides and chalcogenides. Acta Crystallogr.,
Sect. A.

[89] Butler F., Fiorentini F., Eisenhardt K. H. S., Williams C. K. (2025). Structure-Activity
Relationships for s-Block Metal/Co­(III) Heterodinuclear Catalysts
in Cyclohexene Oxide Ring-Opening Copolymerizations. Angew. Chem., Int. Ed..

[90] Singer F. N., Deacy A. C., McGuire T. M., Williams C. K., Buchard A. (2022). Chemical Recycling
of Poly­(Cyclohexene Carbonate) Using a Di-MgII Catalyst. Angew. Chem., Int. Ed..

[91] Smith M. L., McGuire T. M., Buchard A., Williams C. K. (2023). Evaluating Heterodinuclear
Mg­(II)­M­(II) (M = Mn, Fe, Ni, Cu, and Zn) Catalysts for the Chemical
Recycling of Poly­(cyclohexene carbonate). ACS
Catal..

[92] Rosetto G., Vidal F., McGuire T. M., Kerr R. W. F., Williams C. K. (2024). High Molar
Mass Polycarbonates as Closed-Loop Recyclable Thermoplastics. J. Am. Chem. Soc..

[93] Eisenhardt K. H. S., Fiorentini F., Williams C. K. (2024). Understanding the Effect of M­(III)
Choice in Heterodinuclear Polymerization Catalysts. Inorg. Chem..

[94] Fiorentini F., Eisenhardt K. H. S., Deacy A. C., Williams C. K. (2024). Synergic Catalysis:
the Importance of Intermetallic Separation in Co­(III)­K­(I) Catalysts
for Ring Opening Copolymerizations. J. Am. Chem.
Soc..

[95] Diment W. T., Gregory G. L., Kerr R. W. F., Phanopoulos A., Buchard A., Williams C. K. (2021). Catalytic Synergy Using Al­(III) and
Group 1 Metals to Accelerate Epoxide and Anhydride Ring-Opening Copolymerizations. ACS Catal..

[96] Diment W. T., Rosetto G., Ezaz-Nikpay N., Kerr R. W. F., Williams C. K. (2023). A highly
active, thermally robust iron­(III)/potassium­(I) heterodinuclear catalyst
for bio-derived epoxide/anhydride ring-opening copolymerizations. Green Chem..

[97] Shellard E. J. K., Diment W. T., Resendiz-Lara D. A., Fiorentini F., Gregory G. L., Williams C. K. (2024). Al­(III)/K­(I) Heterodinuclear
Polymerization
Catalysts Showing Fast Rates and High Selectivity for Polyester Polyols. ACS Catal..

[98] Manjunatha B. R., Stühler M. R., Quick L., Plajer A. J. (2024). Improved access
to polythioesters by heterobimetallic aluminium catalysis. Chem. Commun..

[99] Asaba H., Iwasaki T., Hatazawa M., Deng J., Nagae H., Mashima K., Nozaki K. (2020). Alternating
Copolymerization of CO2
and Cyclohexene Oxide Catalyzed by Cobalt-Lanthanide Mixed Multinuclear
Complexes. Inorg. Chem..

[100] Nagae H., Matsushiro S., Okuda J., Mashima K. (2023). Cationic tetranuclear
macrocyclic CaCo_3_ complexes as highly active catalysts
for alternating copolymerization of propylene oxide and carbon dioxide. Chem. Sci..

[101] Carrow B. P., Nozaki K. (2014). Transition-Metal-Catalyzed
Functional
Polyolefin Synthesis: Effecting Control through Chelating Ancillary
Ligand Design and Mechanistic Insights. Macromolecules.

[102] Suo H., Solan G. A., Ma Y., Sun W.-H. (2018). Developments in
compartmentalized bimetallic transition metal ethylene polymerization
catalysts. Coord. Chem. Rev..

[103] McInnis J. P., Delferro M., Marks T. J. (2014). Multinuclear
Group
4 Catalysis: Olefin Polymerization Pathways Modified by Strong Metal-Metal
Cooperative Effects. Acc. Chem. Res..

[104] Sampson J., Choi G., Akhtar M. N., Jaseer E. A., Theravalappil R., Al-Muallem H. A., Agapie T. (2017). Olefin Polymerization
by Dinuclear Zirconium Catalysts Based on Rigid Teraryl Frameworks:
Effects on Tacticity and Copolymerization Behavior. Organometallics.

[105] Na S. J., Joe D. J., Sujith S., Han W.-S., Kang S. O., Lee B. Y. (2006). Bimetallic nickel
complexes of macrocyclic
tetraiminodiphenols and their ethylene polymerization. J. Organomet. Chem..

[106] Mu H., Zhou G., Hu X., Jian Z. (2021). Recent advances in
nickel mediated copolymerization of olefin with polar monomers. Coord. Chem. Rev..

[107] Cai Z., Xiao D., Do L. H. (2015). Fine-Tuning Nickel
Phenoxyimine Olefin
Polymerization Catalysts: Performance Boosting by Alkali Cations. J. Am. Chem. Soc..

[108] Annamalai V., DiMauro E. F., Carroll P. J., Kozlowski M. C. (2003). Catalysis
of the Michael Addition Reaction by Late Transition Metal Complexes
of BINOL-Derived Salens. J. Org. Chem..

[109] Handa S., Gnanadesikan V., Matsunaga S., Shibasaki M. (2010). Heterobimetallic Transition Metal/Rare
Earth Metal
Bifunctional Catalysis: A Cu/Sm/Schiff Base Complex for Syn-Selective
Catalytic Asymmetric Nitro-Mannich Reaction. J. Am. Chem. Soc..

[110] Xu Y., Kaneko K., Kanai M., Shibasaki M., Matsunaga S. (2014). Regiodivergent Kinetic Resolution
of Terminal and Internal
rac-Aziridines with Malonates under Dinuclear Schiff Base Catalysis. J. Am. Chem. Soc..

[111] Najafian A., Cundari T. R. (2019). Effect of Appended
S-Block Metal
Ion Crown Ethers on Redox Properties and Catalytic Activity of Mn–Nitride
Schiff Base Complexes: Methane Activation. Inorg.
Chem..

[112] Taut J., Chambron J.-C., Kersting B. (2023). Fifty Years of Inorganic
Biomimetic Chemistry: From the Complexation of Single Metal Cations
to Polynuclear Metal Complexes by Multidentate Thiolate Ligands. Eur. J. Inorg. Chem..

[113] Chakraborty T., Sarkar A., Adhikary A., Chakiroy N., Das D. (2019). Synthesis of Structurally Diverse
Ferrimagnetically and Antiferromagnetically
Coupled M^II^–Mn^II^ (M = Cu, Ni) Heterometallic
Schiff Base Compounds with a Dicyanamide Spacer and Study of Biomimetic
Catalytic Activity. Cryst. Growth Des..

