# Supplementary material for: General, Quantified Structure-Performance Correlations for Synergistic Heteronuclear Electro‑, Polymerization, and Asymmetric Catalysts
Source: ACS Catal. 2025 Jul 16;15(15):12959–83. doi: 10.1021/acscatal.5c02224 (PMC12322920; doi:10.1021/acscatal.5c02224)
Supplement: Supplementary file 1 [file cs5c02224_si_001.pdf]

# Supplementary Information

## General, Quantified Structure-Performance Correlations for Synergic Heteronuclear Electro-, Polymerization, and Asymmetric Catalysts

Katharina H. S. Eisenhardt‡, Francesca Fiorentini‡, Frederica Butler, Rosie Thorogood, Charlotte K. Williams*

‡K.H.S.E and F.F contributed equally and are listed as co-first authors in alphabetical order.

Dept. Chemistry, 12 Mansfield Road, University of Oxford, Oxford, U.K., OX1 3TA.

Figure S1. $L^H$ ligand structures discussed in the review. ........................................................5

Figure S2. $L^A$ ligand structures discussed in the review. ........................................................5

Figure S3. $L^M$ ligand structures discussed in the review. ........................................................6

Figure S4. Structure of heterodinuclear $L^{A1}$Ni(II)M(I-III) catalysts and correlation between the Ni(II/I) redox couple and M(I-III) Lewis acidity (measured by aqua complex p$K_a$).[1] Reproduced from [1]. Copyright 2024 American Chemical Society. ........................................................6

Figure S5. Cyclic voltammetry and UV-vis spectroscopy data for a series of monometallic and heterodinuclear Ni(II) complexes with s-block metals.[2] Reproduced from ref [2]. Available under a CC-BY 3.0 license. Copyright 2019 Kang et al. ........................................................7

Figure S6. Correlations of Ni(II/I) redox potentials and d→d transitions with changing M(I-III) Lewis acidity in $L^{M1}$Ni(II)M(I-III) as reported by Blakemore and co-workers. When p$K_a$ is converted to the same units as $E_{1/2}$, according to $2.303 \cdot RT \cdot \log(K_a) = 59.1$ mV, the gradient is close to unity (- 69 mV / 59 mV = - 1.17).[3] Reproduced from ref [3]. Copyright 2024 American Chemical Society. ......................7

Figure S7. Structure of heterodinuclear $L^{M1}$[VO](II)M(I/II) complexes and correlations between isotropic hyperfine coupling constant ($A_{iso}$), determined by EPR.[4] Reproduced from ref [4]. Copyright 2023 American Chemical Society. ........................................................8

Figure S8. Mechanism for electrocatalytic Fe-catalyzed aerobic oxidation of C-H bonds in cyclohexene to form cyclohexanone and cyclohexanol (bold), showing the formation of the inactive diiron μ-oxo species via oxidation of the Fe(II) species.[5-7] Mechanism reproduced from [8]. Available under a CC-BY 3.0 license. Copyright 2018 Chantarojsiri et al ........................................................8

Figure S9. Equation for dinitrogen formation from Mn(V) nitrido complexes; single electron oxidation of the Mn(V) nitrido complex to Mn(IV) is followed by bimolecular coupling to form $N_2$ and a Mn(III) complex (S = solvent).[9, 10] Reproduced from [9] Copyright 2022 American Chemical Society. ........................................................8

Figure S10. Correlation between Mn(VI/V) redox potential with varying s-block metal charge (K(I) or Ba(I); chosen due to their similar ionic radii) showing a gradient of 189 mV per charge.[10] Adapted with permission from [10]. Copyright 2019 John Wiley and Sons ........................................................9

Figure S11. Graph showing little change in the $L^{M2}$[Mn(V)**N-H**]M(I-III) bond dissociation free energy (BDFE) with changing M(I-III).[9] Reproduced from [9] Copyright 2022 American Chemical Society.....9

Figure S12. Lack of correlations with s-block metal ionic radius and ligand binding affinity (using the binding affinity to 18-crown-6 as an approximation), using activity for PO/$CO_2$ and PO/PA ROCOP using $L^{M2}$Co(III)M(I/II) catalysts as examples.[11-13] Reproduced from [11]. Available under a CC-BY 4.0 license. Copyright 2023 Fiorentini et al. ........................................................10

Figure S13. Linear free energy relationships (LFERs; i.e. $\log_{10}(k_{obs})$ plotted against aqua M(I/II) complex p$K_a$ i.e. $-\log_{10}(K_a)$) for PO/$CO_2$ and PO/PA ROCOP using a series of $L^{M2}$Co(III)M(I/II) catalysts.[11] Reproduced from [11]. Available under a CC-BY 4.0 license. Copyright 2023 Fiorentini et al. ........................................................11

Figure S14. Proposed mechanism for the ROCOP of CHO and $CO_2$ catalyzed by $L^{M2}$Co(III)M(I/II) with the carbonate and alkoxide intermediates highlighted in blue and yellow respectively.[14] ............12

Figure S15. Linear free energy relationships (LFERs; i.e. $\log_{10}(k_{obs})$ plotted against aqua M(I/II) complex p$K_a$ i.e. $-\log_{10}(K_a)$) for CHO/PA and CHO/$CO_2$ (20 bar) ROCOP using a series of

$L^{M2}$Co(III)M(I/II) catalysts.[15] Reproduced from [15]. Available under a CC-BY 4.0 license. Copyright 2025 Butler et al. 13

Figure S16. Top: Structure of $L^{A3}$M(III)K(I) catalysts for the ring-opening copolymerizations of cyclohexene oxide with phthalic anhydride and of cyclohexene oxide with $CO_2$, data from a report by Williams and co-workers. Bottom: Comparison of catalyst activities and selectivities, and measured characterization parameters of the catalysts.[16] Reproduced from [16]. Copyright 2024 American Chemical Society. 14

Figure S17. Series of Co(III)K(I) catalysts with varying intermetallic separations for the ring-opening copolymerization of propene oxide with $CO_2$, and propene oxide with phthalic anhydride, data taken from a report by Williams and co-workers. Reaction conditions: PO/PA ROCOP: 1:20:400:1000 ([a]1:20:400:4000) $[catalyst]_0:[BDM]_0:[PA]_0:[PO]_0$, neat, and 60 °C; PO/$CO_2$ ROCOP: 1:20:4000 ([b]1:20:1000) $[catalyst]_0:[CHD]_0:[PO]_0$, 50 °C, and 20 bar $CO_2$.[17] Reproduced from [17]. Copyright 2024 American Chemical Society. 15

Figure S18. $L^{A4}$Al(III)M(I) catalysts for the ring-opening copolymerization of cyclohexene oxide with phthalic anhydride and their activities, data from a report by Williams and co-workers.[18] Reproduced from [18]. Copyright 2021 American Chemical Society. 16

Figure S19. $L^{A5}$Al(III)M(I-III) catalyst structures and activities for the ROCOP of phthalic thioanhydride (PTA) with cyclohexene oxide (CHO).[19] Reproduced from ref [19]. Available under a CC-BY 3.0 license. Copyright 2024 Manjunatha et al. 16

Figure S20. $L^{A}$Al(III)K(I) catalysts for the ring-opening copolymerization of cyclohexene oxide with phthalic anhydride, with changing imine linkers to change the steric and electronic environment of the Al(III) centre.[20] Reproduced from [20]. Copyright 2024 American Chemical Society. 17

Figure S21. Heterotetranuclear $L^{M7}Co(II)_3Ca(II)$ catalyst reported for the ROCOP of PO and $CO_2$.[21] Reproduced from ref [21]. Available under a CC-BY 3.0 license. Copyright 2023, Nagae et al. 17

Figure S22. Plot showing correlation between s-block metal association constants (LHS y-axis, diamonds) and the activity of the corresponding heterodinuclear catalyst for ethylene polymerization (RHS y-axis, bars).[22] Reproduced from [22]. Copyright 2015 American Chemical Society. 18

Figure S23. Proposed mechanism for Michael addition of benzyl malonate to cyclohexanone; Ni(II) is proposed to bind and activate the cyclohexanone (the electrophile) while the Cs(I) is proposed to i) enhance the basicity of the naphthoxide (which is proposed to deprotonate the malonate) and ii) may enhance the nucleophilicity of the malonate anion.[23] Reproduced from [23]. Copyright 2003 American Chemical Society. 18

Figure S24. Changing activity and enantioselectivity with changing M(II) ionic radius and Lewis acidity (as measured by aqua complex $pK_a$).[24-28] Reproduced from [24]. Copyright 2001 American Chemical Society. 19

Figure S25. Diastereoselectivity of $L^{A7}$Cu(III)M(III) catalysts with changing M(III) for the *syn*-selective asymmetric nitro-Mannich reaction.[25-29] Reproduced from [29]. Copyright 2007 American Chemical Society. 19

Figure S26. Plot showing correlation between enantioselectivity and M(III) Lewis acidity for the *syn*-selective asymmetric nitro-Mannich reaction catalyzed by $L^{A7}$Cu(II)M(III).[29] Reproduced from [29]. Copyright 2007 American Chemical Society. 20

Figure S27. Plot showing correlation between diastereoselectivity and M(III) Lewis acidity for the *anti*-selective asymmetric nitro-aldol coupling catalyzed by $L^{A7}$Pd(II)M(III).[29] Reproduced from [29]. Copyright 2007 American Chemical Society. ........................................................................20

Figure S28. Proposed active catalytic species and mechanism for the *syn*-selective asymmetric nitro-Mannich reaction for the synthesis of β-nitroamines.[29] Reproduced from [29]. Copyright 2007 American Chemical Society. ........................................................................21

Figure S29. Enantioselectivity for *anti*-selective nitro-aldol coupling catalysed by $L^{A7}$Cu(II)M(III).[29] Reproduced from [29]. Copyright 2007 American Chemical Society. ........................................................................21

Figure S30. Effect of changing M(II) on catalyst performance for $L^{A6}$M(II)La(III) catalysts for *anti*-selective asymmetric nitro-aldol reaction.[25-30] Adapted with permission from. Copyright 2008 John Wiley and Sons. ........................................................................22

Figure S31. Plots showing changing activity (LHS) and enantioselectivity (RHS) with changing $M_1$(III) (LHS) and $M_2$(III) (RHS) Lewis acidity for the α-addition of isocyanides to aldehydes catalyzed by $L^{A8}M_1$(III)$M_2$(III).[31] Reproduced from [31]. Copyright 2009 American Chemical Society. ........................................................................23

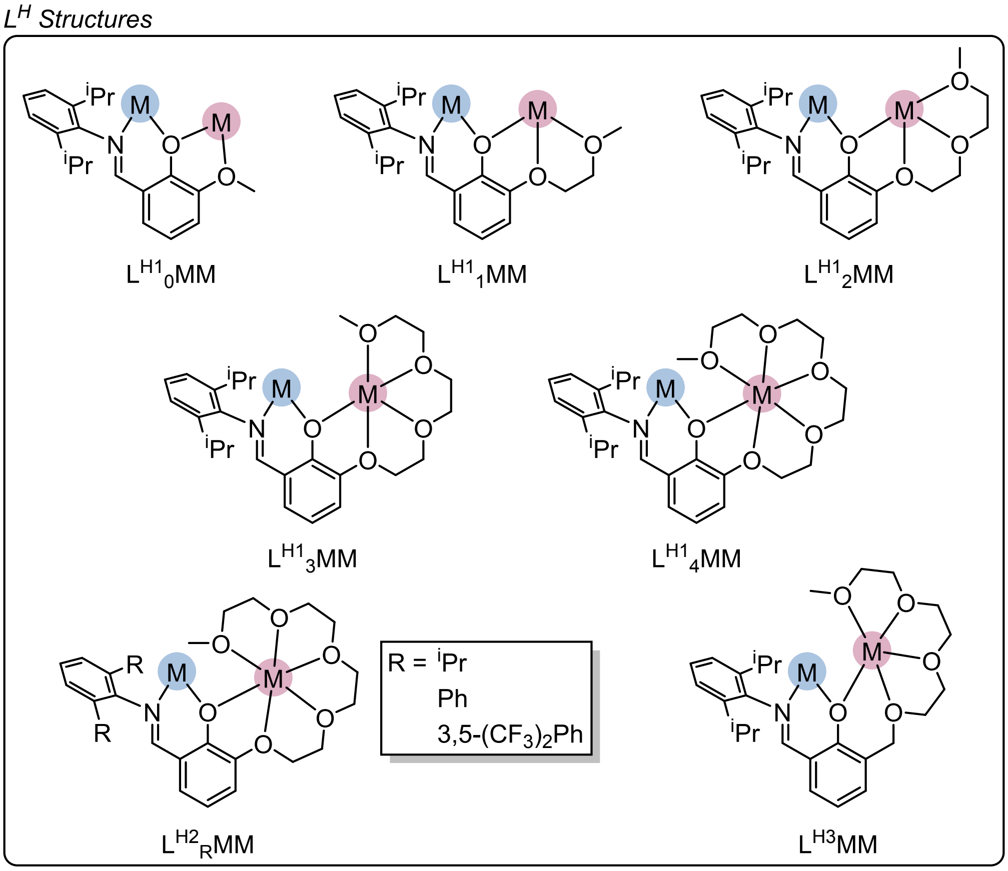


Figure S1. $L^H$ ligand structures discussed in the review.

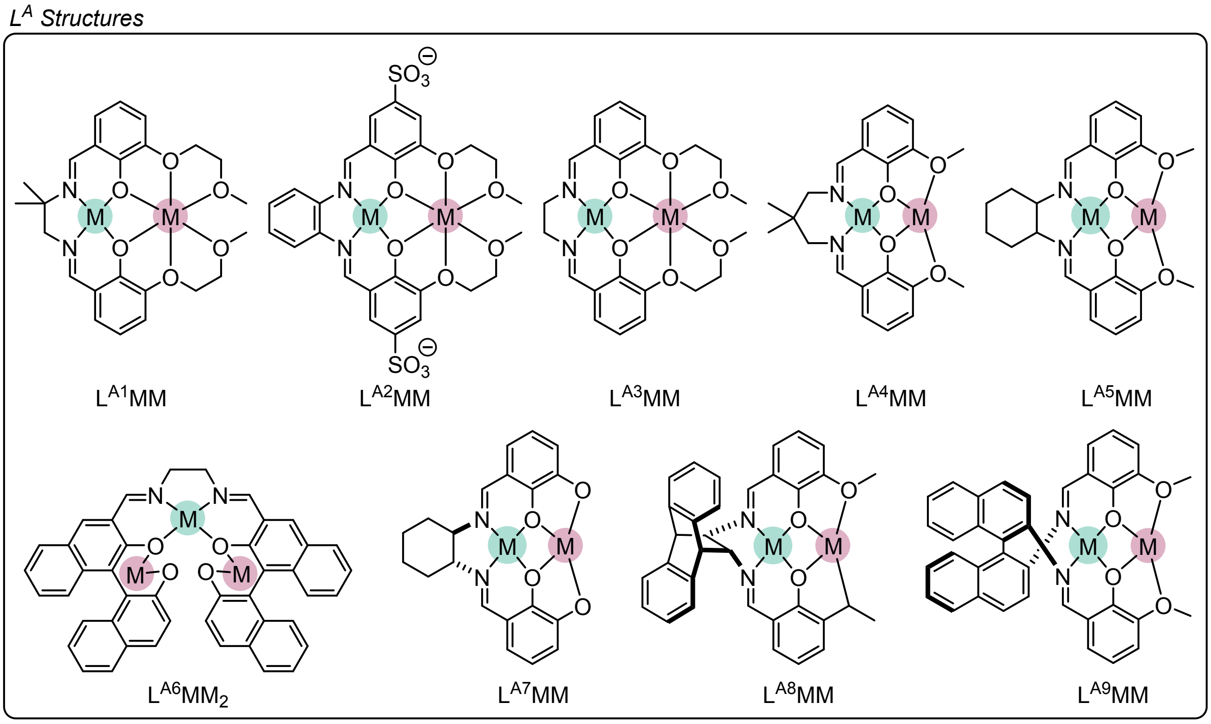


Figure S2. $L^A$ ligand structures discussed in the review.

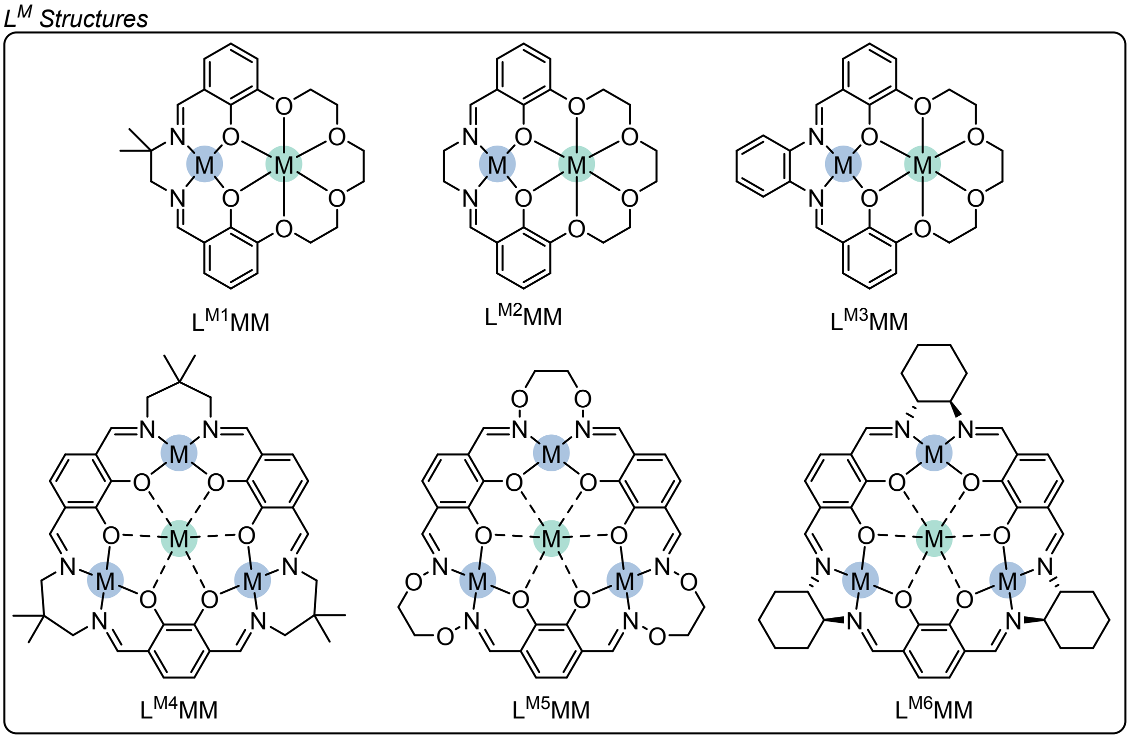


Figure S3. $L^{M}$ ligand structures discussed in the review.

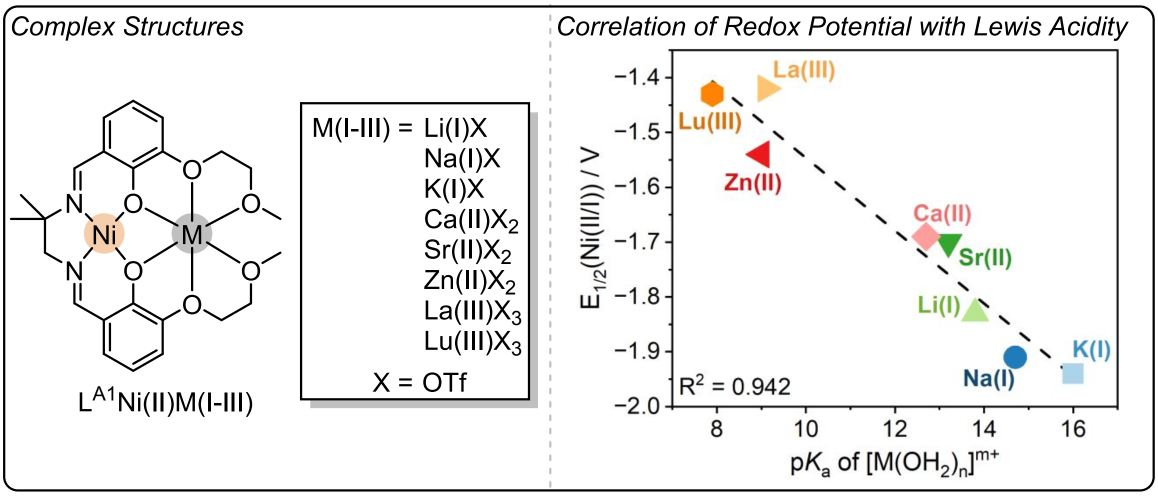


Figure S4. Structure of heterodinuclear $L^{A1}$Ni(II)M(I-III) catalysts and correlation between the Ni(II/I) redox couple and M(I-III) Lewis acidity (measured by aqua complex p$K_a$).[1] Reproduced from ref.[1]. Copyright 2024 American Chemical Society.

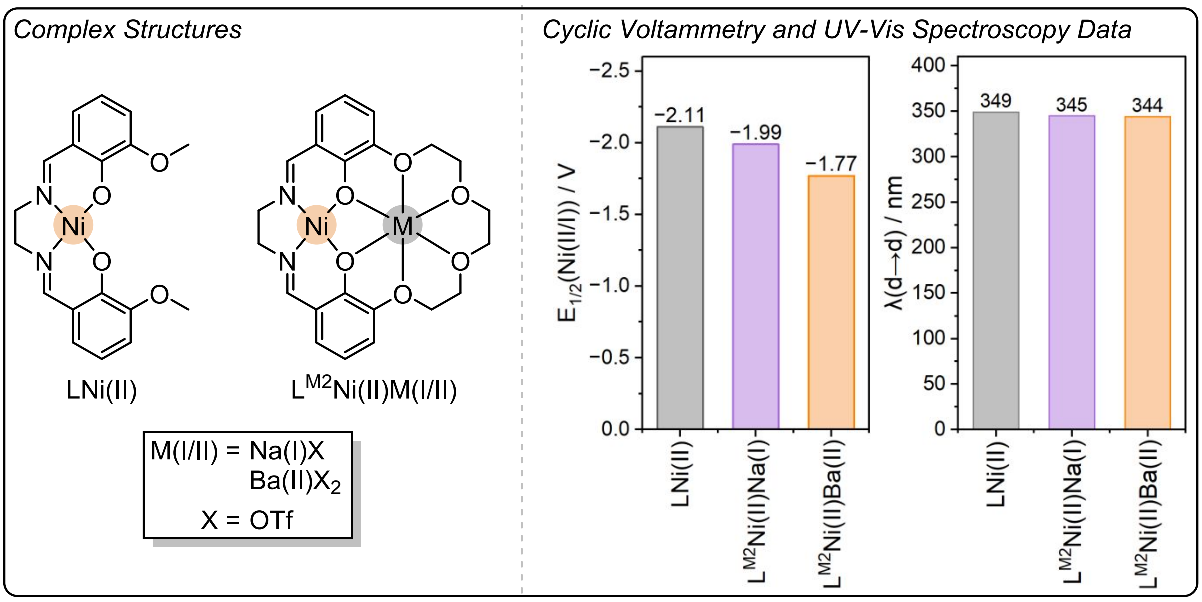


Figure S5. Cyclic voltammetry and UV-vis spectroscopy data for a series of monometallic and heterodinuclear Ni(II) complexes with s-block metals.[2] Reproduced from ref. [2]. Available under a CC-BY 3.0 license. Copyright 2019 Kang et al.

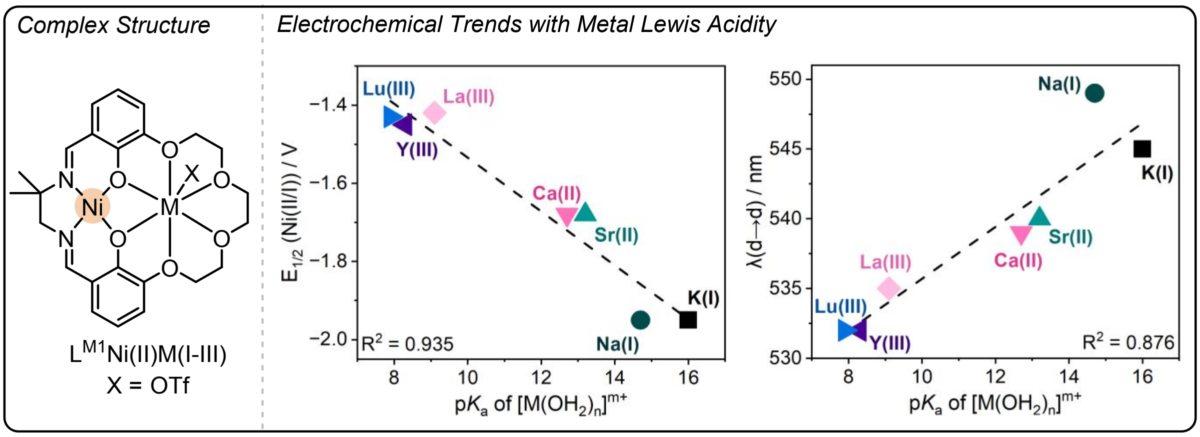


Figure S6. Correlations of Ni(II/I) redox potentials and d→d transitions with changing M(I-III) Lewis acidity in $L^{M1}$Ni(II)M(I-III) as reported by Blakemore and co-workers. When p$K_a$ is converted to the same units as $E_{1/2}$, according to $2.303 \cdot RT \cdot \log(K_a) = 59.1$ mV, the gradient is close to unity (- 69 mV / 59 mV = - 1.17).[3] Reproduced from ref [3]. Copyright 2024 American Chemical Society.

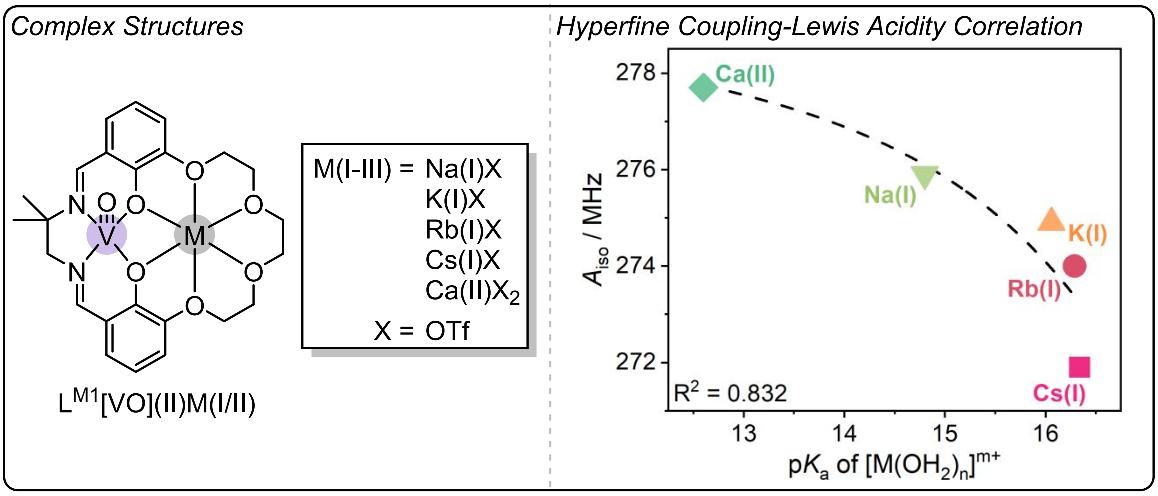


Figure S7. Structure of heterodinuclear $L^{M1}$[VO](II)M(I/II) complexes and correlations between isotropic hyperfine coupling constant ($A_{iso}$), determined by EPR.[4] Reproduced from ref [4]. Copyright 2023 American Chemical Society.

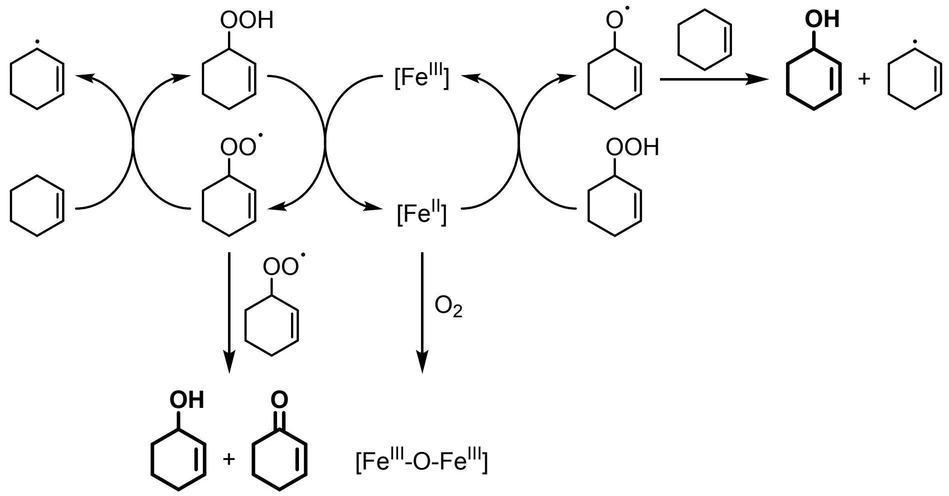


Figure S8. Mechanism for electrocatalytic Fe-catalyzed aerobic oxidation of C-H bonds in cyclohexene to form cyclohexanone and cyclohexanol (bold), showing the formation of the inactive diiron μ-oxo species via oxidation of the Fe(II) species.[5-7] Mechanism reproduced from ref. [8]. Available under a CC-BY 3.0 license. Copyright 2018 Chantarojsiri et al

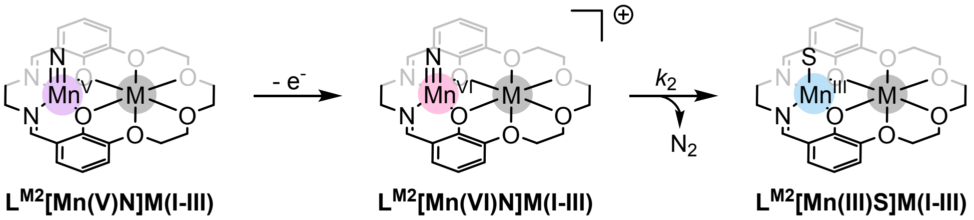


Figure S9. Equation for dinitrogen formation from Mn(V) nitrido complexes; single electron oxidation of the Mn(V) nitrido complex to Mn(IV) is followed by bimolecular coupling to form $N_2$ and a Mn(III) complex (S = solvent).[9, 10] Reproduced from ref.[9] Copyright 2022 American Chemical Society.

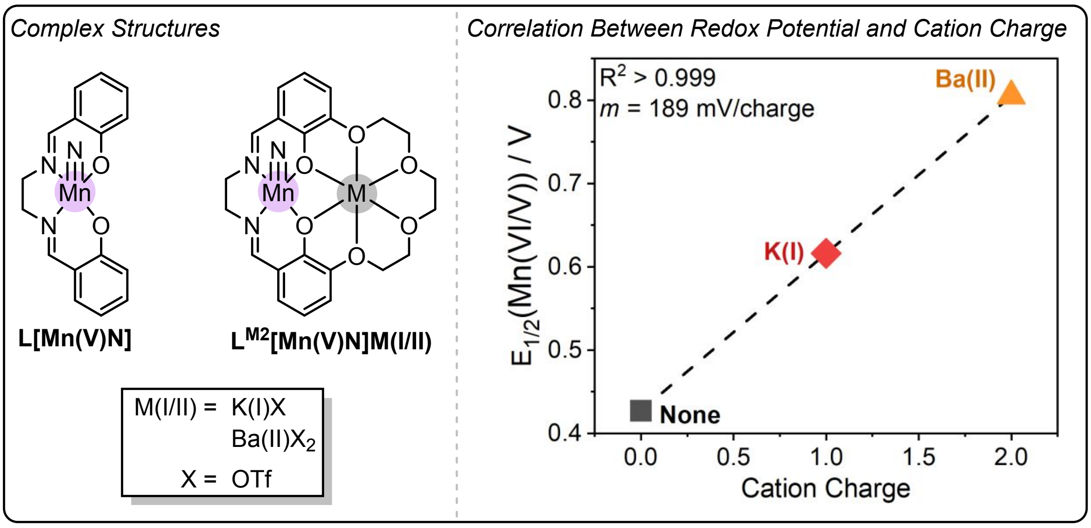


Figure S10. Correlation between Mn(VI/V) redox potential with varying s-block metal charge (K(I) or Ba(I); chosen due to their similar ionic radii) showing a gradient of 189 mV per charge.[10] Adapted with permission from ref.[10]. Copyright 2019 John Wiley and Sons

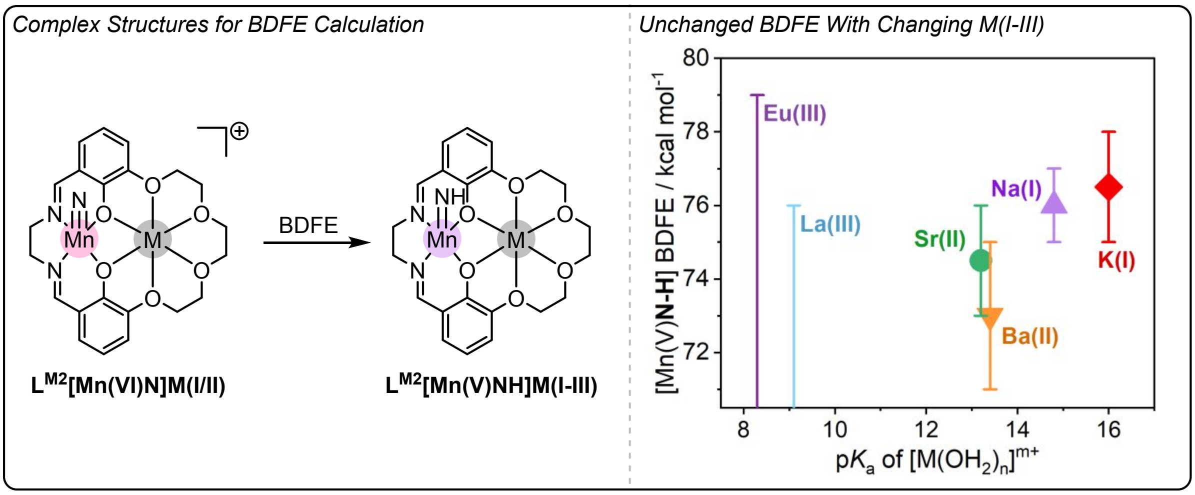


Figure S11. Graph showing little change in the L$^{M2}$[Mn(V)**N-H**]M(I-III) bond dissociation free energy (BDFE) with changing M(I-III).[9] Reproduced from ref.[9] Copyright 2022 American Chemical Society.

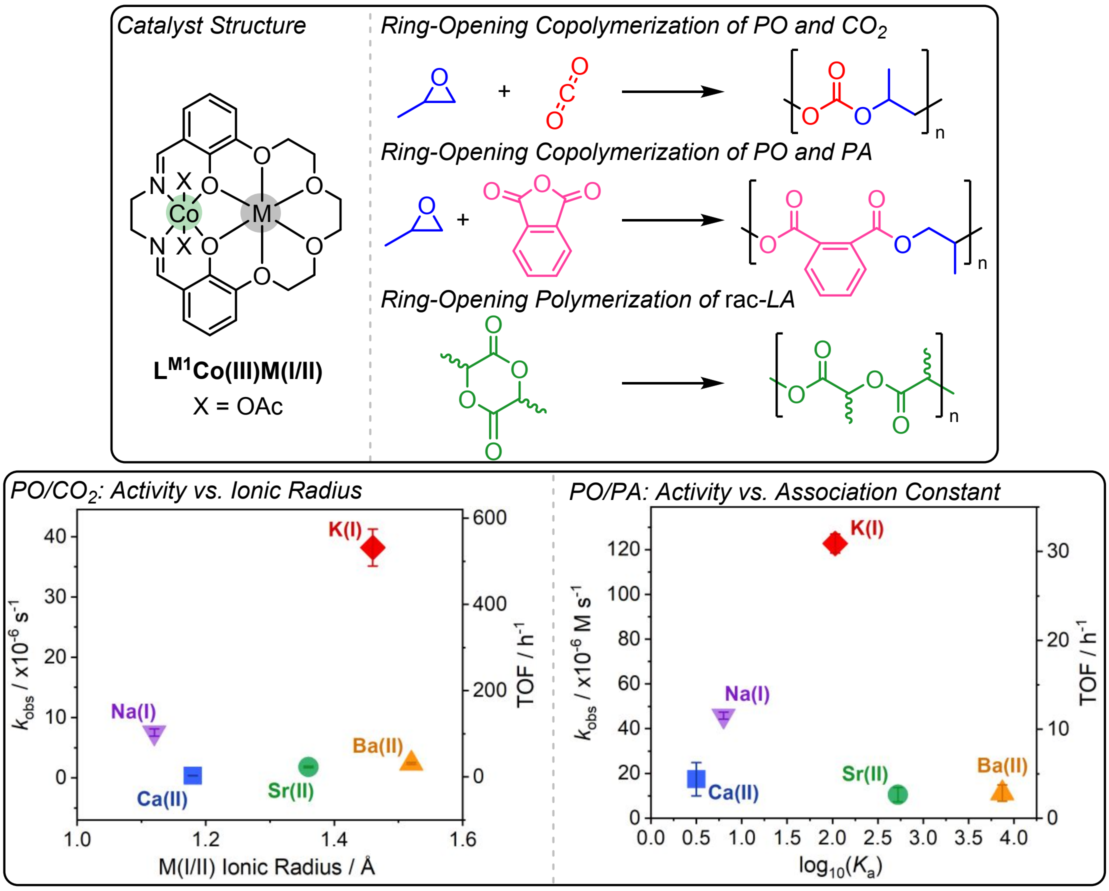


Figure S12. Lack of correlations with s-block metal ionic radius and ligand binding affinity (using the binding affinity to 18-crown-6 as an approximation), using activity for $PO/CO_2$ and PO/PA ROCOP using $L^{M2}$Co(III)M(I/II) catalysts as examples.[11-13] Reproduced from ref.[11]. Available under a CC-BY 4.0 license. Copyright 2023 Fiorentini et al.

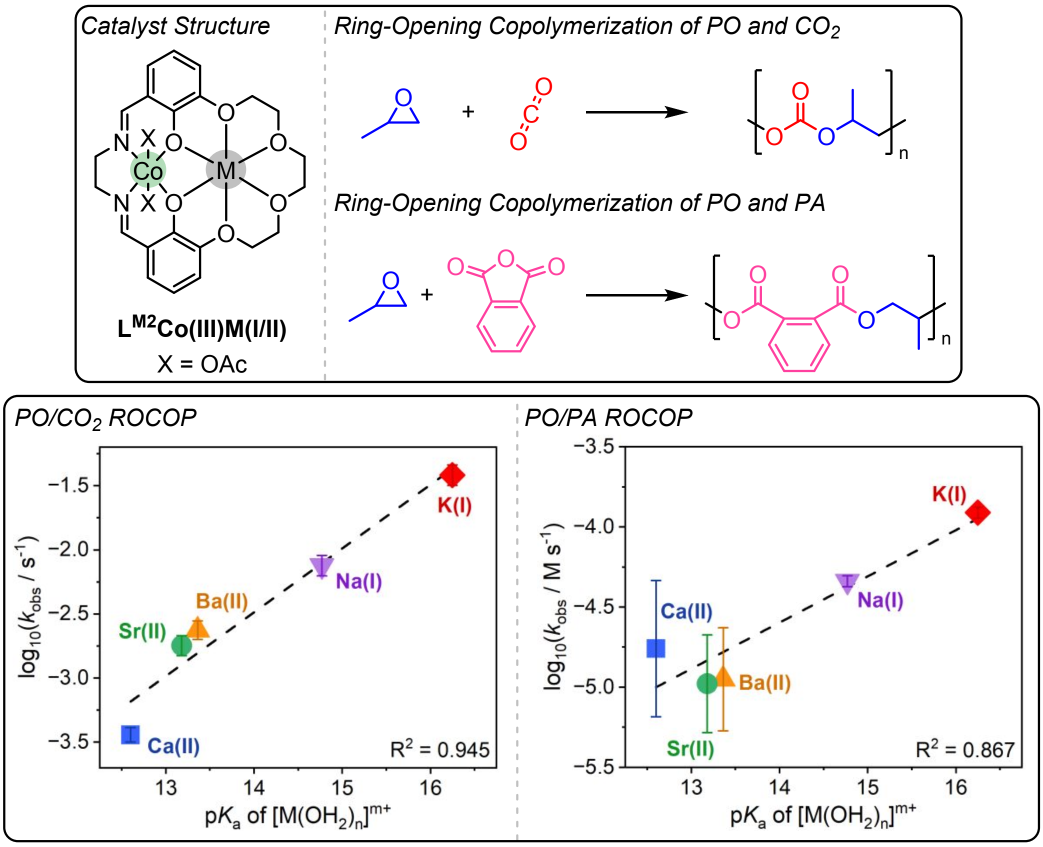


Figure S13. Linear free energy relationships (LFERs; i.e. $\log_{10}(k_{obs})$ plotted against aqua M(I/II) complex p$K_a$ i.e. $-\log_{10}(K_a)$) for PO/$CO_2$ and PO/PA ROCOP using a series of $L^{M2}$Co(III)M(I/II) catalysts.[11] Reproduced from ref.[11]. Available under a CC-BY 4.0 license. Copyright 2023 Fiorentini et al.

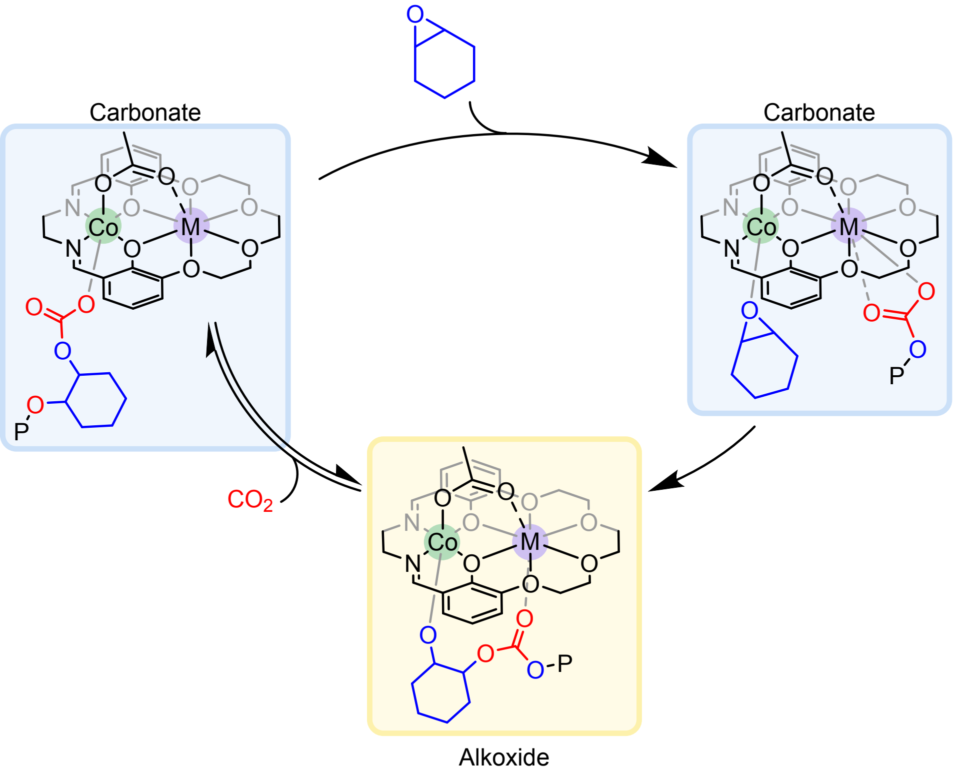


Figure S14. Proposed mechanism for the ROCOP of CHO and $CO_2$ catalyzed by $L^{M2}$Co(III)M(I/II) with the carbonate and alkoxide intermediates highlighted in blue and yellow respectively.[14]

When the $CO_2$ pressure is high, the equilibrium between the alkoxide and carbonate is proposed to lie far towards the carbonate species. At reduced $CO_2$ pressures, the equilibrium is proposed to shift towards the alkoxide intermediate. This change in the equilibrium position is proposed to result in differences in the relative rates across the catalyst series such that the correlation is linear rather than exponential'; at high $CO_2$ pressures, the concentration of the alkoxide intermediate is proposed to be negligible. As such, the predominant species is expected to be the carbonate, which may be able to coordinate in a bidentate manner, perhaps resulting in the observed exponential correlation. When the $CO_2$ pressure is reduced, the concentration of the monodentate alkoxide intermediate is proposed to increase, which may lead to the observed linear correlation

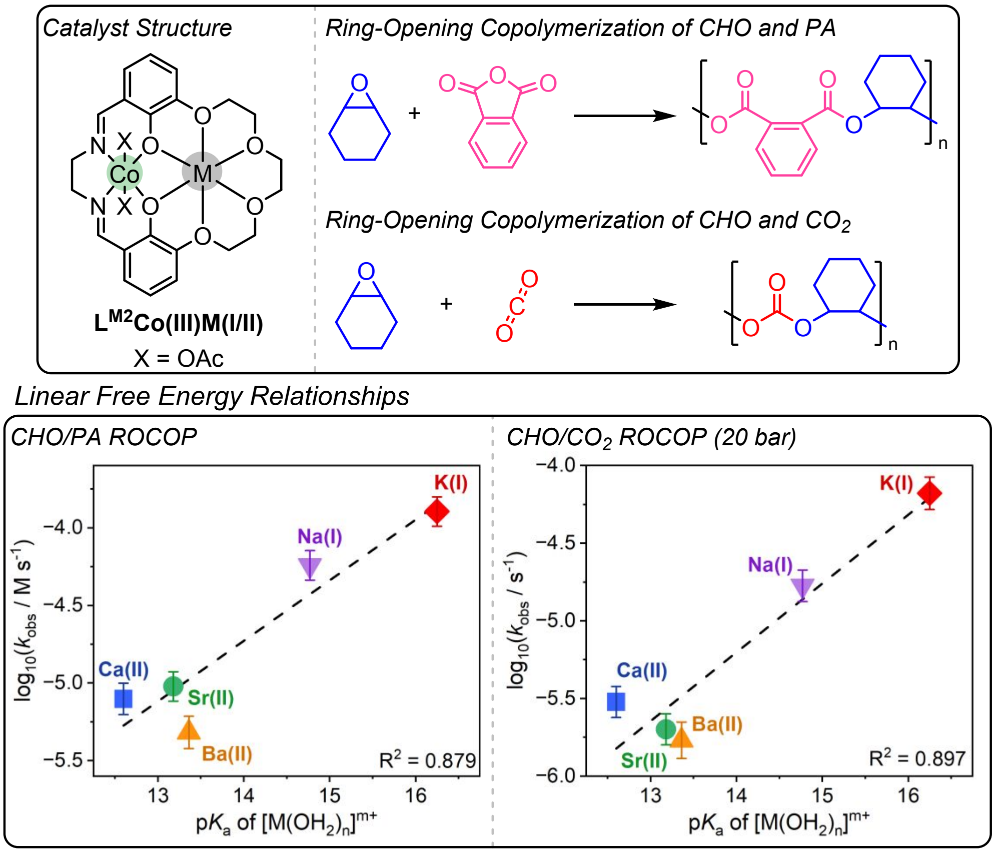


Figure S15. Linear free energy relationships (LFERs; i.e. $\log_{10}(k_{obs})$ plotted against aqua M(I/II) complex p$K_a$ i.e. $-\log_{10}(K_a)$) for CHO/PA and CHO/$CO_2$ (20 bar) ROCOP using a series of $L^{M2}$Co(III)M(I/II) catalysts.[15] Reproduced from ref.[15]. Available under a CC-BY 4.0 license. Copyright 2025 Butler et al.

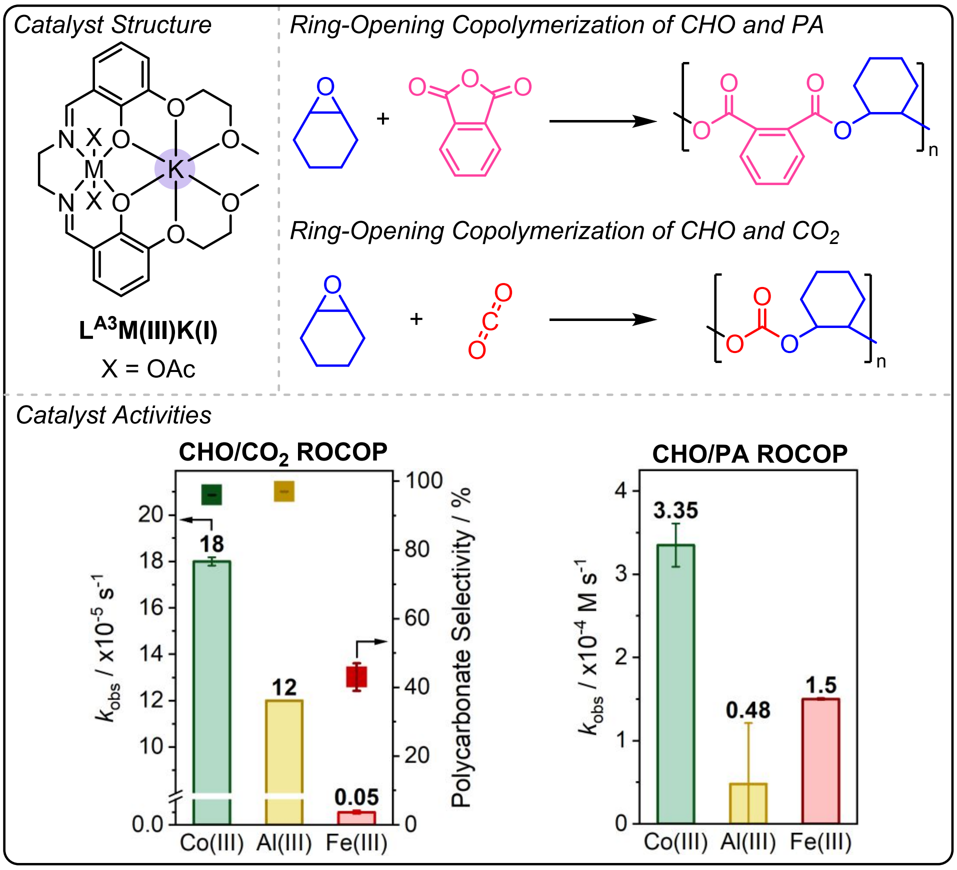


Figure S16. Top: Structure of L[A3]M(III)K(I) catalysts for the ring-opening copolymerizations of cyclohexene oxide with phthalic anhydride and of cyclohexene oxide with $CO_2$, data from a report by Williams and co-workers. Bottom: Comparison of catalyst activities and selectivities, and measured characterization parameters of the catalysts.[16] Reproduced from ref.[16]. Copyright 2024 American Chemical Society.

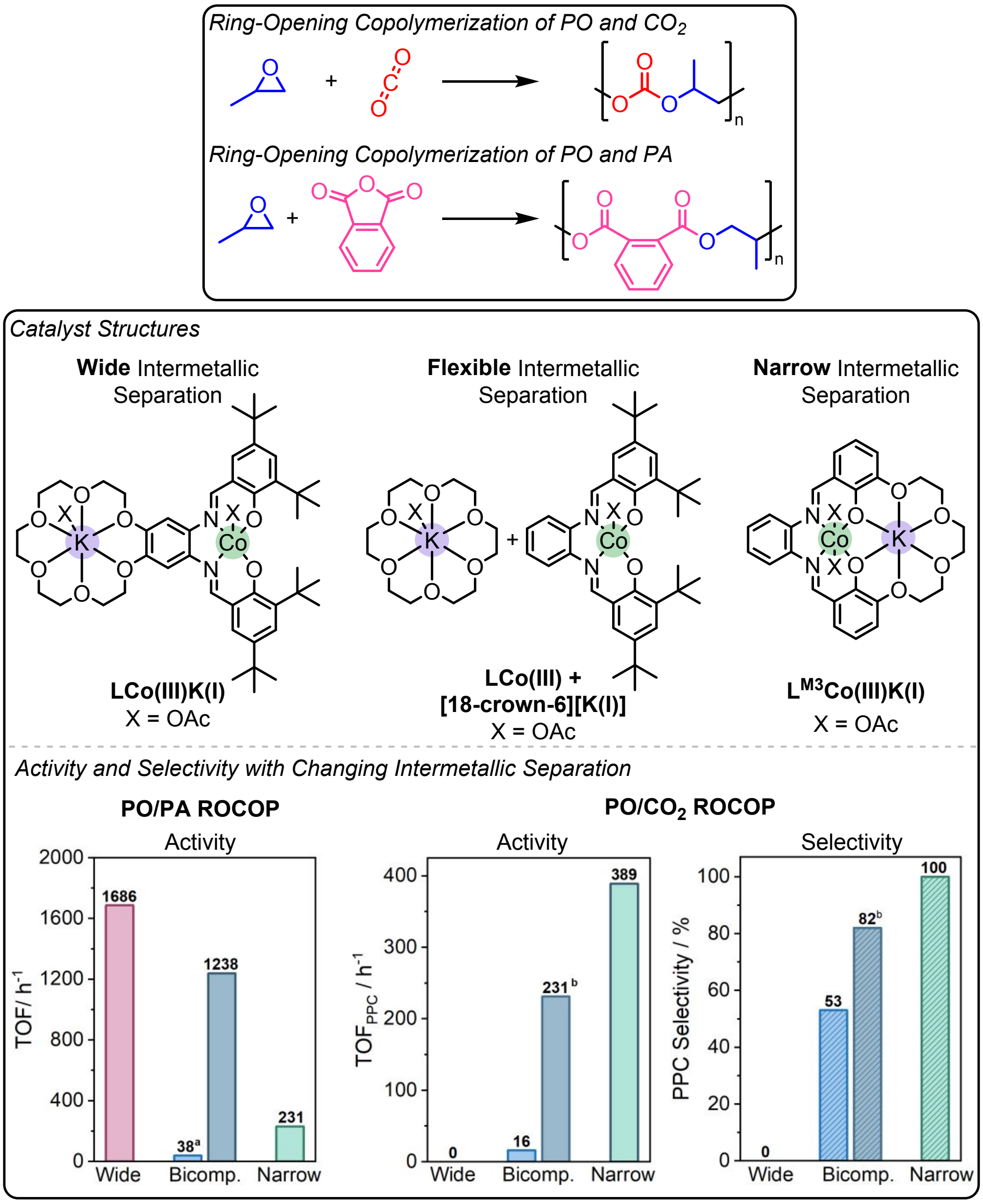


Figure S17. Series of Co(III)K(I) catalysts with varying intermetallic separations for the ring-opening copolymerization of propene oxide with $CO_2$, and propene oxide with phthalic anhydride, data taken from a report by Williams and co-workers. Reaction conditions: PO/PA ROCOP: 1:20:400:1000 ([a]1:20:400:4000) $[catalyst]_0:[BDM]_0:[PA]_0:[PO]_0$, neat, and 60 °C; PO/$CO_2$ ROCOP: 1:20:4000 ([b]1:20:1000) $[catalyst]_0:[CHD]_0:[PO]_0$, 50 °C, and 20 bar $CO_2$.[17] Reproduced from ref.[17]. Copyright 2024 American Chemical Society.

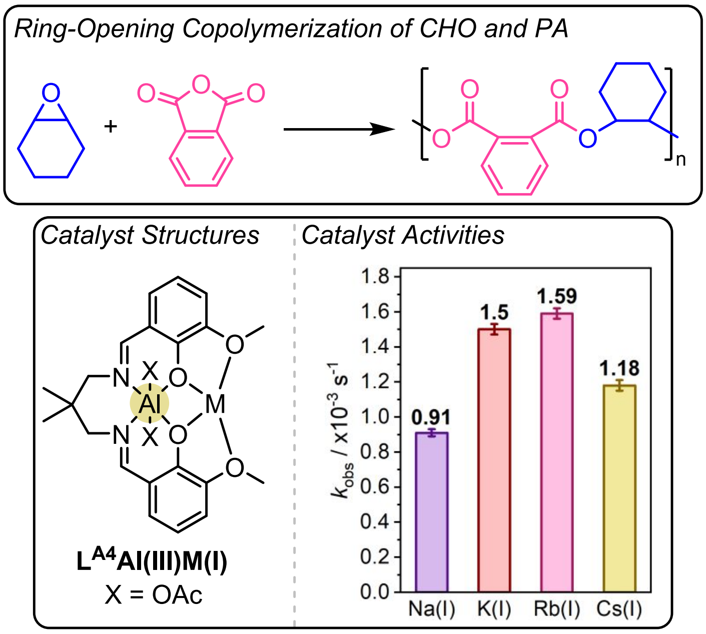


Figure S18. L[A4]Al(III)M(I) catalysts for the ring-opening copolymerization of cyclohexene oxide with phthalic anhydride and their activities, data from a report by Williams and co-workers.[18] Reproduced from [18]. Copyright 2021 American Chemical Society.

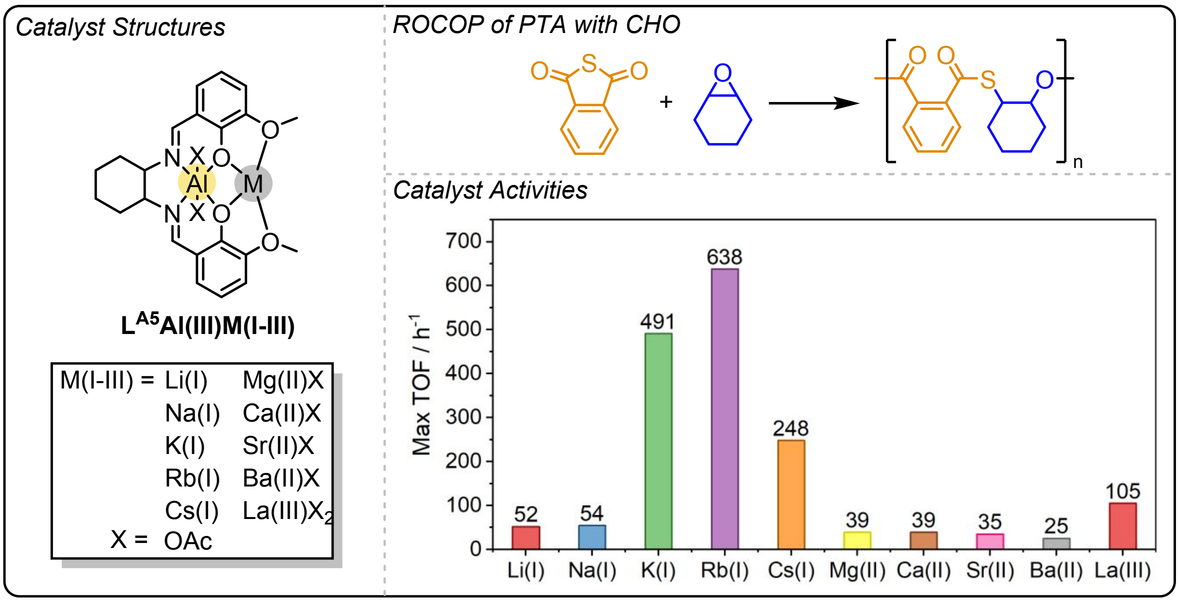


Figure S19. L[A5]Al(III)M(I-III) catalyst structures and activities for the ROCOP of phthalic thioanhydride (PTA) with cyclohexene oxide (CHO).[19] Reproduced from ref [19]. Available under a CC-BY 3.0 license. Copyright 2024 Manjunatha et al.

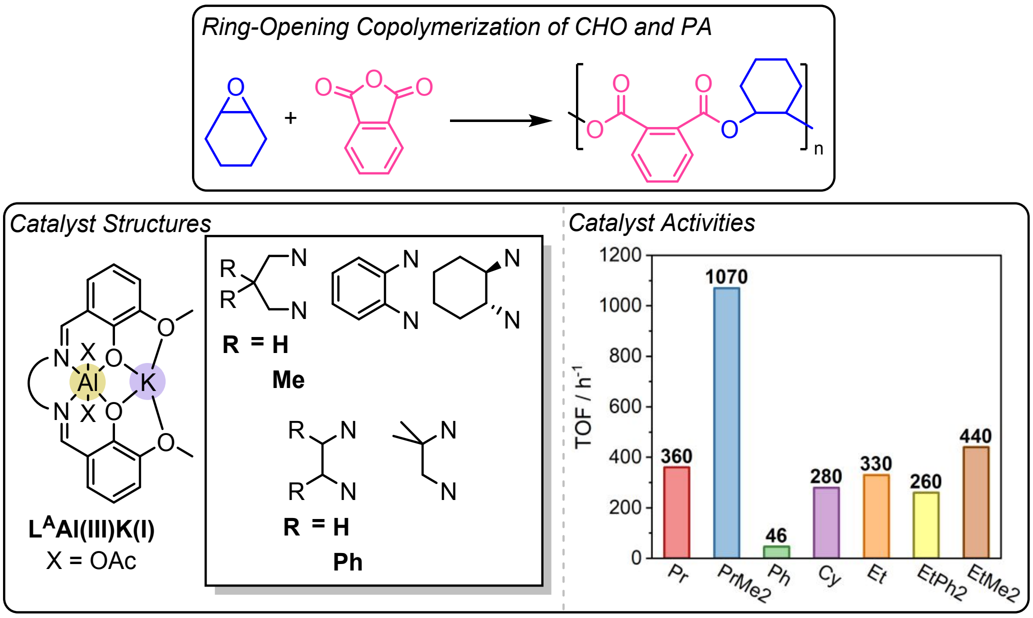


Figure S20. $L^{A}$Al(III)K(I) catalysts for the ring-opening copolymerization of cyclohexene oxide with phthalic anhydride, with changing imine linkers to change the steric and electronic environment of the Al(III) centre.[20] Reproduced from ref.[20]. Copyright 2024 American Chemical Society.

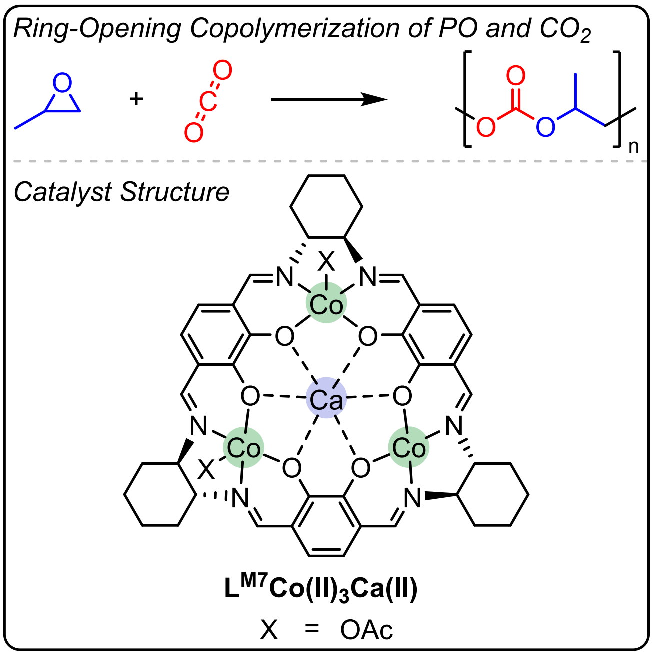


Figure S21. Heterotetranuclear $L^{M7}$Co(II)$_3$Ca(II) catalyst reported for the ROCOP of PO and $CO_2$.[21] Reproduced from ref [21]. Available under a CC-BY 3.0 license. Copyright 2023, Nagae et al.

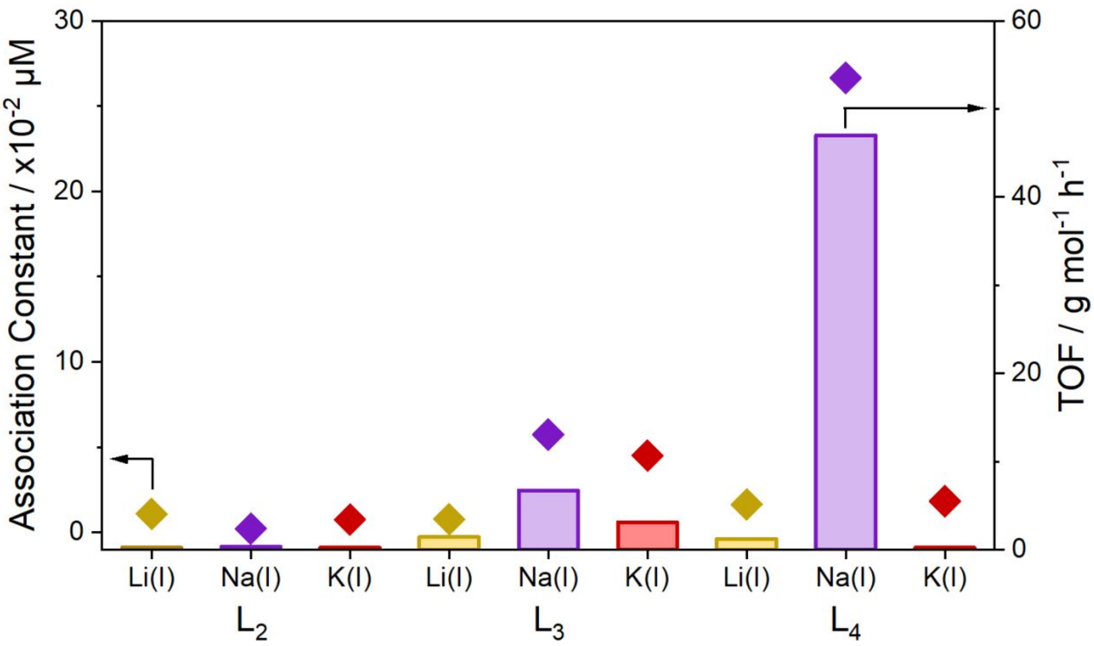


Figure S22. Plot showing correlation between s-block metal association constants (LHS y-axis, diamonds) and the activity of the corresponding heterodinuclear catalyst for ethylene polymerization (RHS y-axis, bars).[22] Reproduced from ref.[22]. Copyright 2015 American Chemical Society.

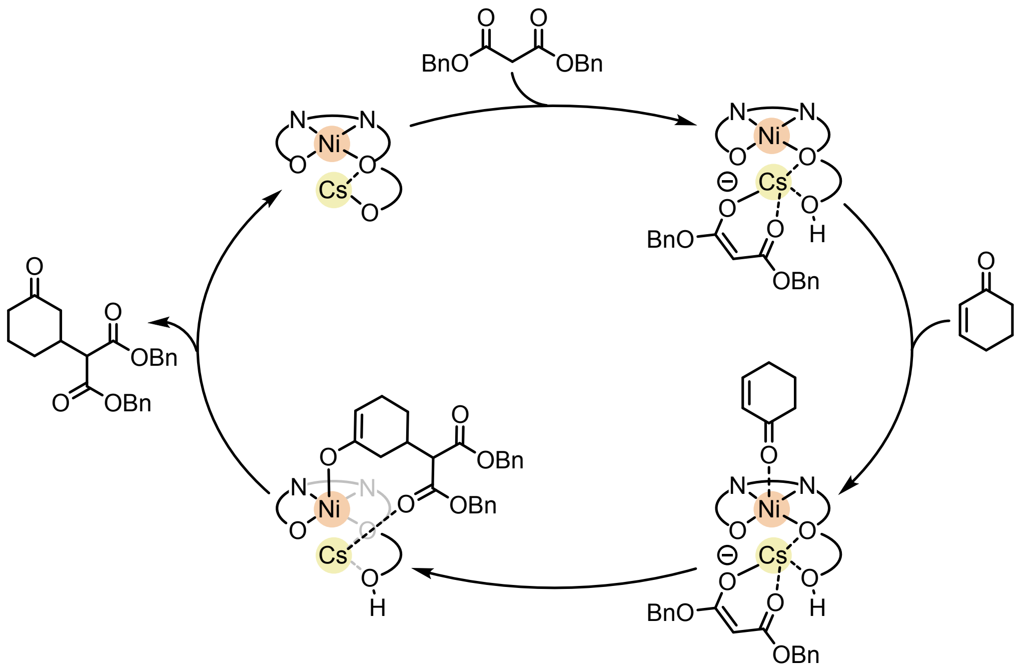

Figure S23. Proposed mechanism for Michael addition of benzyl malonate to cyclohexanone; Ni(II) is proposed to bind and activate the cyclohexanone (the electrophile) while the Cs(I) is proposed to i) enhance the basicity of the naphthoxide (which is proposed to deprotonate the malonate) and ii) may enhance the nucleophilicity of the malonate anion.[23] Reproduced from ref.[23]. Copyright 2003 American Chemical Society.

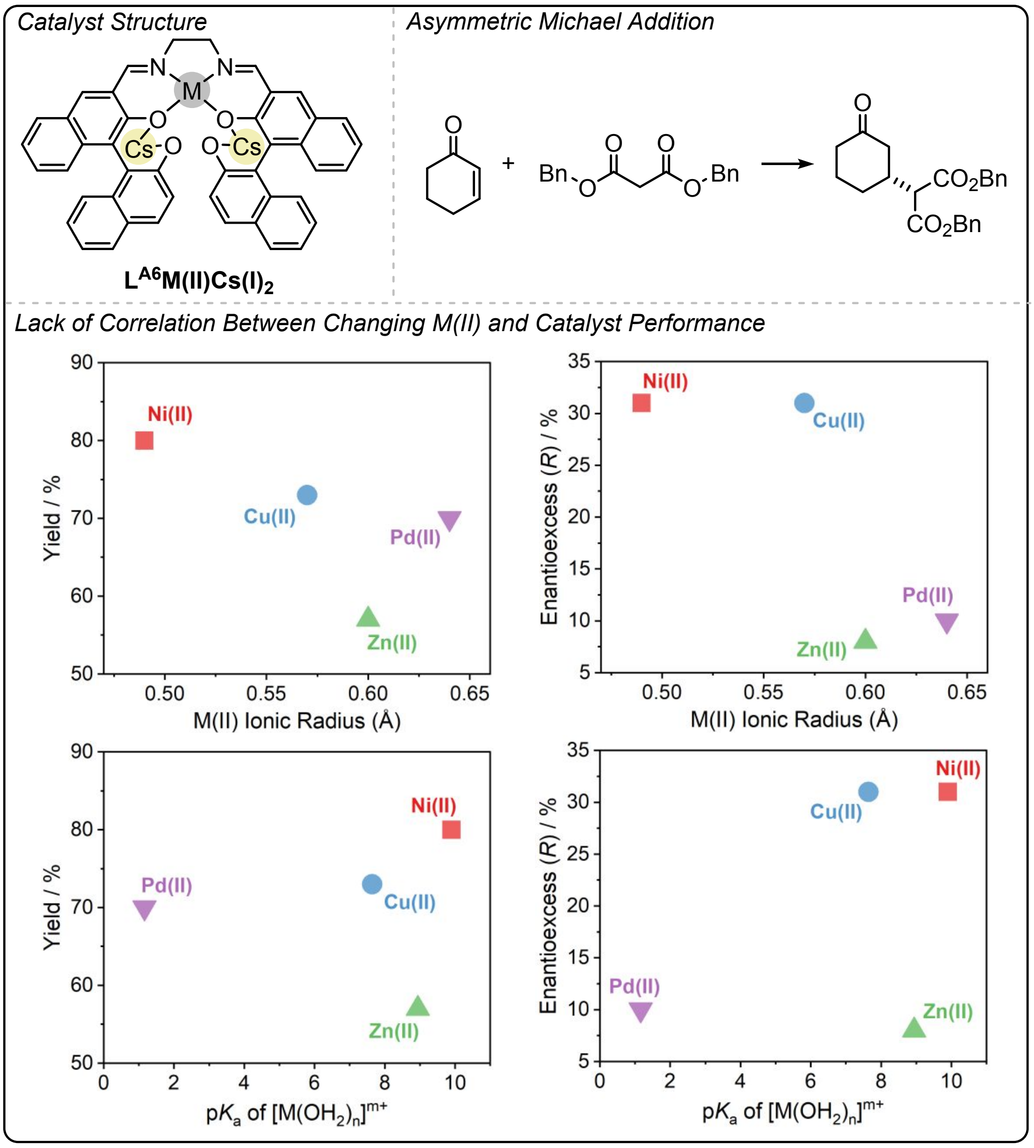


Figure S24. Changing activity and enantioselectivity with changing M(II) ionic radius and Lewis acidity (as measured by aqua complex p$K_a$).[24-28] Reproduced from ref.[24]. Copyright 2001 American Chemical Society.

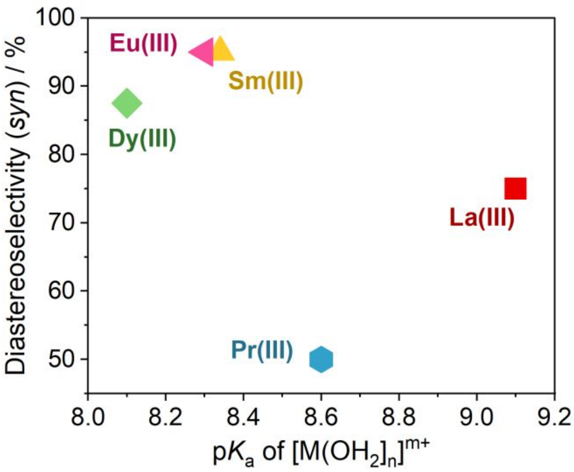


Figure S25. Diastereoselectivity of L$^{A7}$Cu(III)M(III) catalysts with changing M(III) for the *syn*-selective asymmetric nitro-Mannich reaction.[25-29] Reproduced from ref.[29]. Copyright 2007 American Chemical Society.

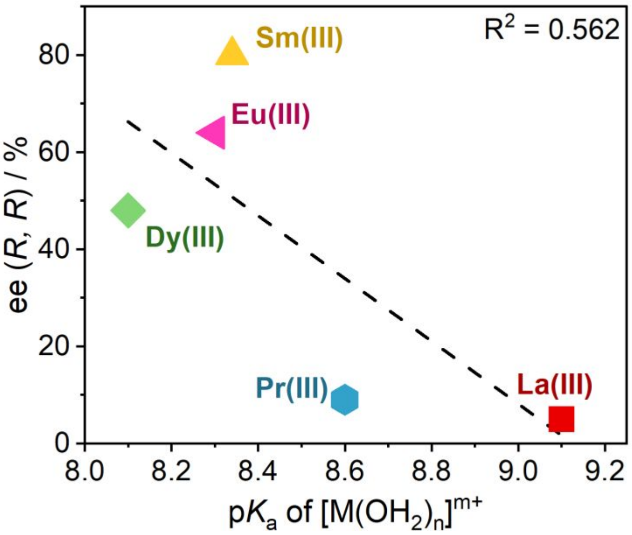


Figure S26. Plot showing correlation between enantioselectivity and M(III) Lewis acidity for the *syn*-selective asymmetric nitro-Mannich reaction catalyzed by $L^{A7}$Cu(II)M(III).[29] Reproduced from ref.[29]. Copyright 2007 American Chemical Society.

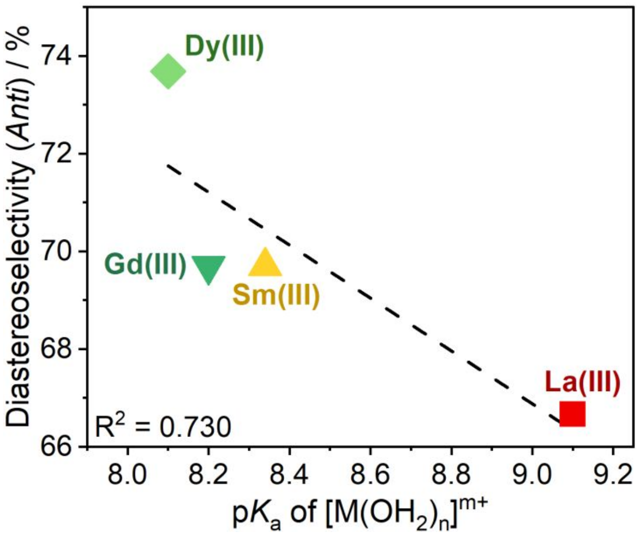


Figure S27. Plot showing correlation between diastereoselectivity and M(III) Lewis acidity for the *anti*-selective asymmetric nitro-aldol coupling catalyzed by $L^{A7}$Pd(II)M(III).[29] Reproduced from ref.[29]. Copyright 2007 American Chemical Society.

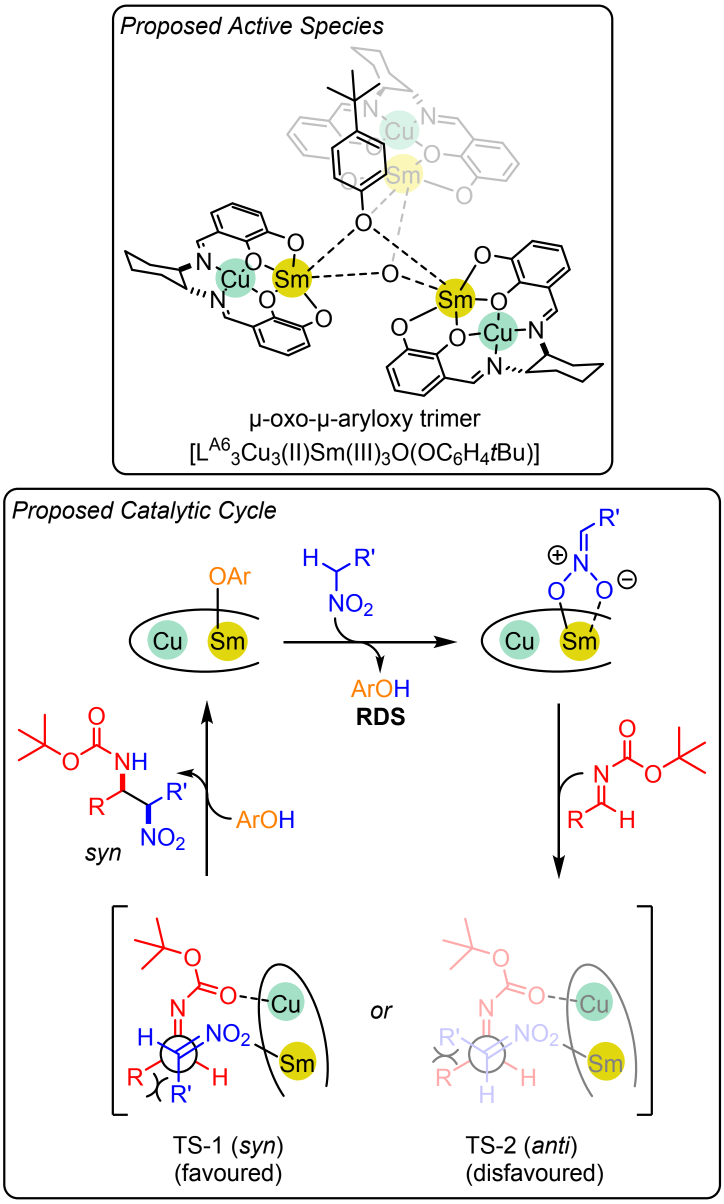


Figure S28. Proposed active catalytic species and mechanism for the *syn*-selective asymmetric nitro-Mannich reaction for the synthesis of β-nitroamines.[29] Reproduced from ref.[29]. Copyright 2007 American Chemical Society.

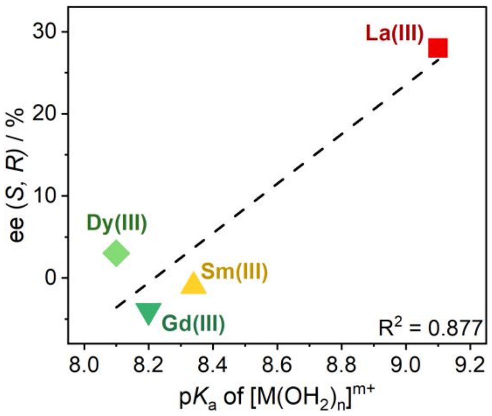


Figure S29. Enantioselectivity for *anti*-selective nitro-aldol coupling catalysed by $L^{A7}$Cu(II)M(III).[29] Reproduced from ref.[29]. Copyright 2007 American Chemical Society.

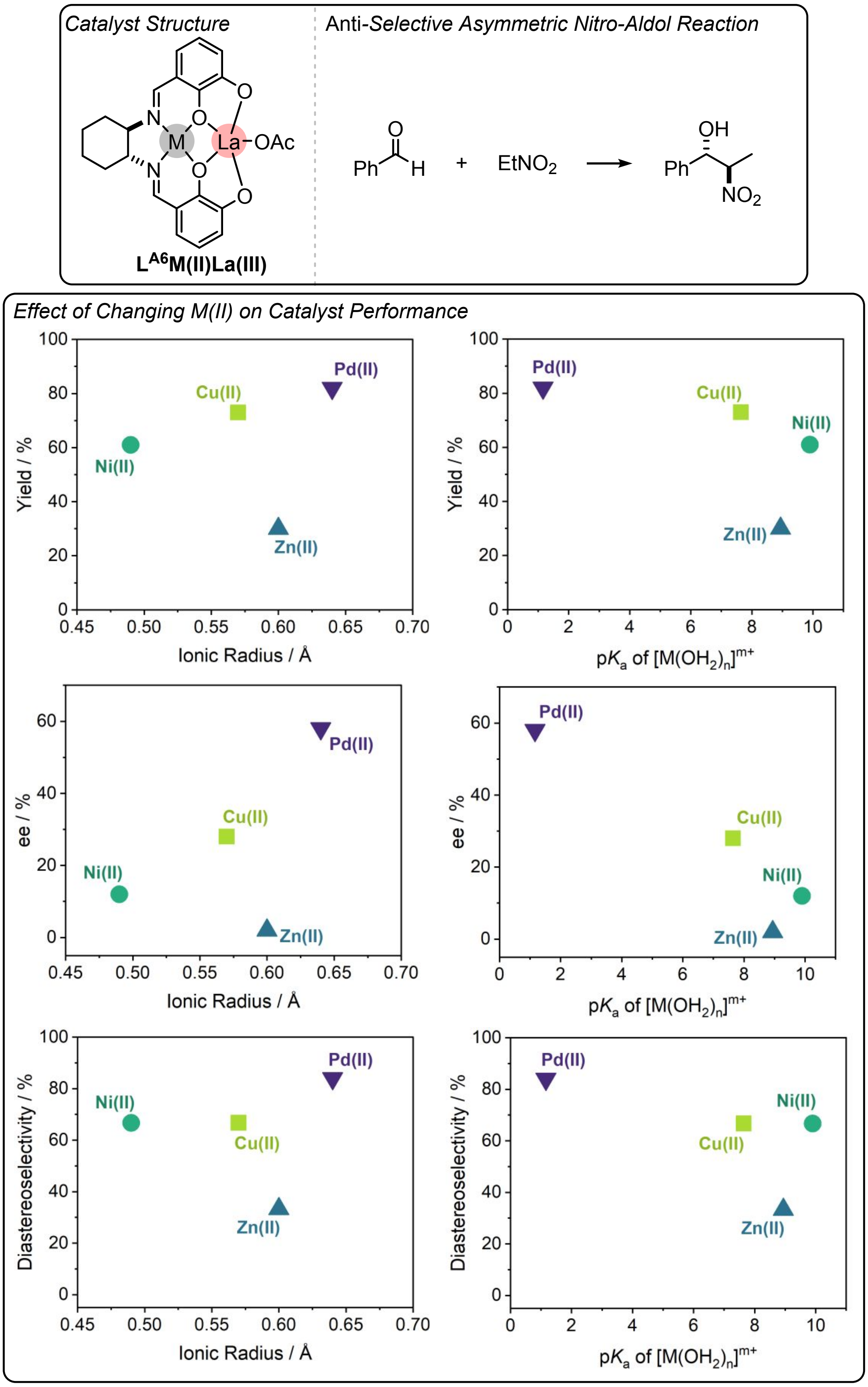


Figure S30. Effect of changing M(II) on catalyst performance for $L^{A6}$M(II)La(III) catalysts for *anti*-selective asymmetric nitro-aldol reaction.[25-28, 30] Adapted with permission from ref.[30]. Copyright 2008 John Wiley and Sons.

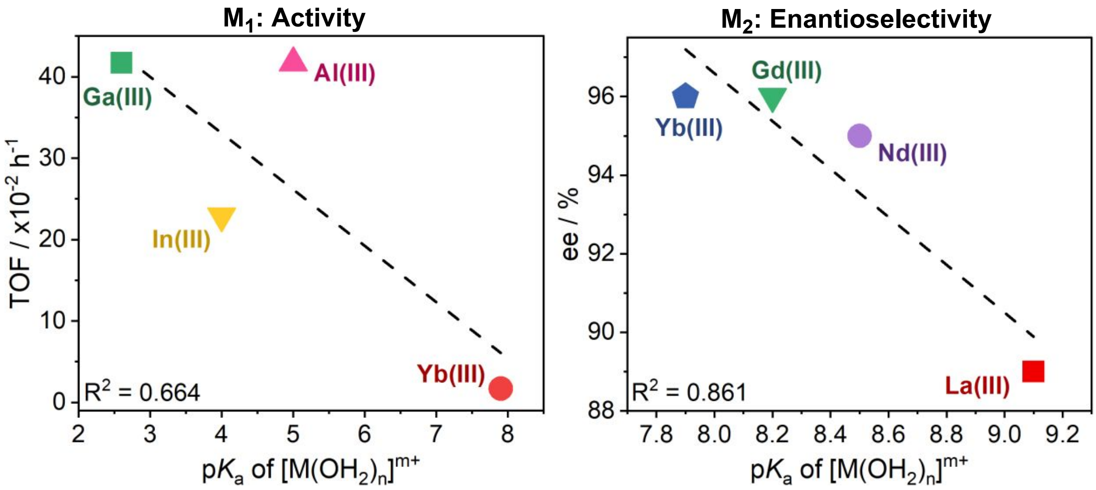


Figure S31. Plots showing changing activity (LHS) and enantioselectivity (RHS) with changing $M_1$(III) (LHS) and $M_2$(III) (RHS) Lewis acidity for the α-addition of isocyanides to aldehydes catalyzed by $L^{A8}M_1$(III)$M_2$(III).[31] Reproduced from ref.[31]. Copyright 2009 American Chemical Society.

## References

(1) Karnes, J. P.; Lind, N. M.; Oliver, A. G.; Day, C. S.; Day, V. W.; Blakemore, J. D. Tunability in Heterobimetallic Complexes Featuring an Acyclic "Tiara" Polyether Motif. *Inorg. Chem.* **2024**, *64* (1), 571-593. DOI: 10.1021/acs.inorgchem.4c03352.

(2) Kang, K.; Fuller, J.; Reath, A. H.; Ziller, J. W.; Alexandrova, A. N.; Yang, J. Y. Installation of internal electric fields by non-redox active cations in transition metal complexes. *Chem. Sci.* **2019**, *10* (43), 10135-10142. DOI: 10.1039/C9SC02870F.

(3) Karnes, J. P.; Kumar, A.; Hopkins Leseberg, J. A.; Day, V. W.; Blakemore, J. D. Trivalent Cations Slow Electron Transfer to Macrocyclic Heterobimetallic Complexes. *Inorg. Chem.* **2024**, *63* (19), 8710-8729. DOI: 10.1021/acs.inorgchem.4c00230.

(4) Dopp, C. M.; Golwankar, R. R.; Kelsey, S. R.; Douglas, J. T.; Erickson, A. N.; Oliver, A. G.; Day, C. S.; Day, V. W.; Blakemore, J. D. Vanadyl as a Spectroscopic Probe of Tunable Ligand Donor Strength in Bimetallic Complexes. *Inorg. Chem.* **2023**, *62* (25), 9827-9843. DOI: 10.1021/acs.inorgchem.3c00724.

(5) Böttcher, A.; Birnbaum, E. R.; Day, M. W.; Gray, H. B.; Grinstaff, M. W.; Labinger, J. A. How do electronegative substituents make metal complexes better catalysts for the oxidation of hydrocarbons by dioxygen? *J. Mol. Catal. A Chem.* **1997**, *117* (1), 229-242. DOI: 10.1016/S1381-1169(96)00299-3.

(6) Grinstaff, M. W.; Hill, M. G.; Labinger, J. A.; Gray, H. B. Mechanism of Catalytic Oxygenation of Alkanes by Halogenated Iron Porphyrins. *Science* **1994**, *264* (5163), 1311-1313. DOI: 10.1126/science.8191283.

(7) Labinger, J. A. A simplified model for catalyzed isobutane autoxidation: implications for the mechanism of catalysis by halogenated porphyrin complexes. *Catal. Lett.* **1994**, *26* (1), 95-99. DOI: 10.1007/BF00824035.

(8) Chantarojsiri, T.; Ziller, J. W.; Yang, J. Y. Incorporation of redox-inactive cations promotes iron catalyzed aerobic C–H oxidation at mild potentials. *Chem. Sci.* **2018**, *9* (9), 2567-2574. DOI: 10.1039/C7SC04486K.

(9) Léonard, N. G.; Chantarojsiri, T.; Ziller, J. W.; Yang, J. Y. Cationic Effects on the Net Hydrogen Atom Bond Dissociation Free Energy of High-Valent Manganese Imido Complexes. *J. Am. Chem. Soc.* **2022**, *144* (4), 1503-1508. DOI: 10.1021/jacs.1c09583.

(10) Chantarojsiri, T.; Reath, A. H.; Yang, J. Y. Cationic Charges Leading to an Inverse Free-Energy Relationship for N−N Bond Formation by MnVI Nitrides. *Angew. Chem., Int. Ed.* **2018**, *57* (43), 14037-14042. DOI: 10.1002/anie.201805832.

(11) Fiorentini, F.; Diment, W. T.; Deacy, A. C.; Kerr, R. W. F.; Faulkner, S.; Williams, C. K. Understanding catalytic synergy in dinuclear polymerization catalysts for sustainable polymers. *Nat. Commun.* **2023**, *14* (1), 4783. DOI: 10.1038/s41467-023-40284-z.

(12) Izatt, R. M.; Terry, R. E.; Haymore, B. L.; Hansen, L. D.; Dalley, N. K.; Avondet, A. G.; Christensen, J. J. Calorimetric titration study of the interaction of several uni- and bivalent cations with 15-crown-5, 18-crown-6, and two isomers of dicyclohexo-18-crown-6 in aqueous solution at 25.degree.C and .mu. = 0.1. *J. Am. Chem. Soc.* **1976**, *98* (24), 7620-7626. DOI: 10.1021/ja00440a028.

(13) Shannon, R. D. *Acta Cryst A.* **1976**, *32* (5), 751-767. DOI: 10.1107/S0567739476001551.

(14) Eisenhardt, K. H. S.; Fiorentini, F.; Lindeboom, W.; Williams, C. K. Quantifying $CO_2$ Insertion Equilibria for Low-Pressure Propene Oxide and Carbon Dioxide Ring Opening Copolymerization Catalysts. *J. Am. Chem. Soc.* **2024**, *146* (15), 10451-10464. DOI: 10.1021/jacs.3c13959.

(15) Butler, F.; Fiorentini, F.; Eisenhardt, K. H. S.; Williams, C. K. Structure-Activity Relationships for s-Block Metal/Co(III) Heterodinuclear Catalysts in Cyclohexene Oxide Ring-Opening Copolymerizations. *Angew. Chem., Int. Ed.* **2025**, *64* (12), e202422497. DOI: 10.1002/anie.202422497.

(16) Eisenhardt, K. H. S.; Fiorentini, F.; Williams, C. K. Understanding the Effect of M(III) Choice in Heterodinuclear Polymerization Catalysts. *Inorg. Chem.* **2024**, *63* (49), 23438-23449. DOI: 10.1021/acs.inorgchem.4c04430.

(17) Fiorentini, F.; Eisenhardt, K. H. S.; Deacy, A. C.; Williams, C. K. Synergic Catalysis: the Importance of Intermetallic Separation in Co(III)K(I) Catalysts for Ring Opening Copolymerizations. *J. Am. Chem. Soc.* **2024**, *146* (33), 23517-23528. DOI: 10.1021/jacs.4c07405.

(18) Diment, W. T.; Gregory, G. L.; Kerr, R. W. F.; Phanopoulos, A.; Buchard, A.; Williams, C. K. Catalytic Synergy Using Al(III) and Group 1 Metals to Accelerate Epoxide and Anhydride Ring-Opening Copolymerizations. *ACS Catal.* **2021**, *11* (20), 12532-12542. DOI: 10.1021/acscatal.1c04020.

(19) Manjunatha, B. R.; Stühler, M. R.; Quick, L.; Plajer, A. J. Improved access to polythioesters by heterobimetallic aluminium catalysis. *Chem. Comm.* **2024**, *60* (34), 4541-4544. DOI: 10.1039/D4CC00811A.

(20) Shellard, E. J. K.; Diment, W. T.; Resendiz-Lara, D. A.; Fiorentini, F.; Gregory, G. L.; Williams, C. K. Al(III)/K(I) Heterodinuclear Polymerization Catalysts Showing Fast Rates and High Selectivity for Polyester Polyols. *ACS Catal.* **2024**, *14* (3), 1363-1374. DOI: 10.1021/acscatal.3c05712.

(21) Nagae, H.; Matsushiro, S.; Okuda, J.; Mashima, K. Cationic tetranuclear macrocyclic $CaCo_3$ complexes as highly active catalysts for alternating copolymerization of propylene oxide and carbon dioxide. *Chem. Sci.* **2023**, *14* (31), 8262-8268. DOI: 10.1039/D3SC00974B.

(22) Cai, Z.; Xiao, D.; Do, L. H. Fine-Tuning Nickel Phenoxyimine Olefin Polymerization Catalysts: Performance Boosting by Alkali Cations. *J. Am. Chem. Soc.* **2015**, *137* (49), 15501-15510. DOI: 10.1021/jacs.5b10351.

(23) Annamalai, V.; DiMauro, E. F.; Carroll, P. J.; Kozlowski, M. C. Catalysis of the Michael Addition Reaction by Late Transition Metal Complexes of BINOL-Derived Salens. *J. Org. Chem.* **2003**, *68* (5), 1973-1981. DOI: 10.1021/jo025993t.

(24) DiMauro, E. F.; Kozlowski, M. C. BINOL−Salen Metal Catalysts Incorporating a Bifunctional Design. *Org. Lett.* **2001**, *3* (11), 1641-1644. DOI: 10.1021/ol0158213.

(25) Kumar, A.; Blakemore, J. D. On the Use of Aqueous Metal-Aqua pKa Values as a Descriptor of Lewis Acidity. *Inorg. Chem.* **2021**, *60* (2), 1107-1115. DOI: 10.1021/acs.inorgchem.0c03239.

(26) First Transition Series Metals. In *Hydrolysis of Metal Ions*, Brown, P. L., Ekberg, C. Eds.; 2016; pp 499-716.

(27) Second and Third Series Transition Metals. In *Hydrolysis of Metal Ions*, 2016; pp 717-756.

(28) Perrin, D. D. *Ionisation Constants of Inorganic Acids and Bases in Aqueous Solution*; Elsevier, 1982. DOI: 10.1016/B978-0-08-029214-4.50008-8.

(29) Handa, S.; Gnanadesikan, V.; Matsunaga, S.; Shibasaki, M. syn-Selective Catalytic Asymmetric Nitro-Mannich Reactions Using a Heterobimetallic Cu−Sm−Schiff Base Complex. *J. Am. Chem. Soc.* **2007**, *129* (16), 4900-4901. DOI: 10.1021/ja0701560.

(30) Handa, S.; Nagawa, K.; Sohtome, Y.; Matsunaga, S.; Shibasaki, M. A Heterobimetallic Pd/La/Schiff Base Complex for anti-Selective Catalytic Asymmetric Nitroaldol Reactions and Applications to Short Syntheses of β-Adrenoceptor Agonists. *Angew. Chem., Int. Ed.* **2008**, *47* (17), 3230-3233. DOI: 10.1002/anie.200705617.

(31) Mihara, H.; Xu, Y.; Shepherd, N. E.; Matsunaga, S.; Shibasaki, M. A Heterobimetallic Ga/Yb-Schiff Base Complex for Catalytic Asymmetric α-Addition of Isocyanides to Aldehydes. *J. Am. Chem. Soc.* **2009**, *131* (24), 8384-8385. DOI: 10.1021/ja903158x.
